# Target-oriented design of helical nanotube molecules for rolled incommensurate bilayers

**DOI:** 10.1038/s42004-022-00777-2

**Published:** 2022-11-19

**Authors:** Hiroyuki Isobe, Yuki Kotani, Taisuke Matsuno, Toshiya M. Fukunaga, Koki Ikemoto

**Affiliations:** Department of Chemistry, The University of Tokyo, Hongo 7-3-1, Bunkyo-ku, Tokyo, 113-0033 Japan

**Keywords:** Stereochemistry, Carbon nanotubes and fullerenes, Supramolecular chemistry

## Abstract

Incommensurate double-wall carbon nanotubes give rise to unique stereochemistry originating from twisted stacks of hexagon arrays. However, atomic-level studies on such unique systems have rarely been performed, even though syntheses of molecular segments of carbon nanotubes have been extensively explored. The design of cylindrical molecules with chirality, particularly, in pairs provides synthetic challenges, because relationships between diameters specified with chiral indices and structures of arylene panels have not been investigated in a systematic manner. Here we show that a molecular version of incommensurate double-wall carbon nanotubes can be designed through the development of an atlas for the top-down design of cylindrical molecules. A large-bore cylindrical molecule with a diameter of 1.77 nm was synthesized using a readily available pigment and encapsulated a small-bore cylindrical molecule with a diameter of 1.04 nm. The large- and small-bore molecules possessed helicity in atomic arrangements, and their coaxial assembly proceeded in nonstereoselective manner to give both heterohelical and homohelical combinations.

## Abstract (web summary)

Incommensurate double-wall carbon nanotubes give rise to unique stereochemistry originating from twisted stacks of hexagon arrays, but atomic-level studies of molecular analogues are hindered by the challenges in designing and synthesizing pairs of chiral cylindrical molecules. Here, a molecular version of incommensurate double-wall carbon nanotubes is designed by development of a roadmap of synthetically accessible chiral indices.

## Introduction

**Fig. 1:**
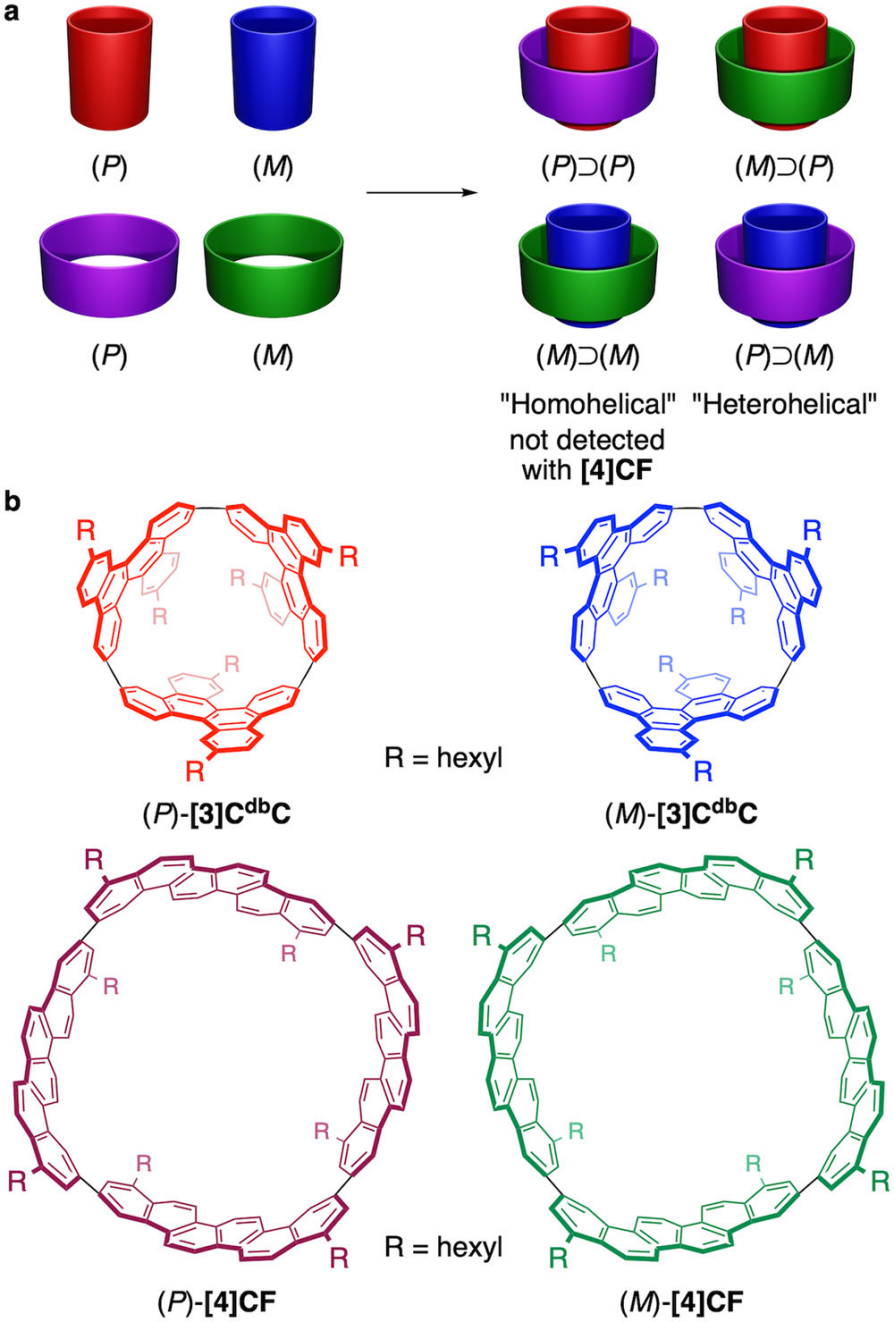
Incommensurate pairs of nanotube molecules. **a** Mixing a racemic mixture of small-bore (*P*)/(*M*)-nanotubes with a racemic mixture of large-bore (*P*)/(*M*)-nanotubes affords four diastereomeric i-DWNT complexes. **b** Finite nanotube molecules that afforded i-DWNT complexes in the previous study^8^. Heterohelical combinations of (*P*)⊃(*M*) and (*M*)⊃(*P*) were selectively obtained.

Graphitic networks of sp^2^-carbon atoms continuously attract much attention in materials science. Modulability of electronic characters of graphitic network, for instance, by periodicity controls and/or heteroatom doping is one of the most intriguing features of the graphitic networks. Incommensurate pairs of two-dimensional atomic layers have attracted considerable attention in particular^1,2^ because bilayers with twisted orientations give rise to unique periodicity that can alter the electronic characteristics of layered materials. The incommensurate bilayer systems can be also found in a pair of helical carbon nanotubes as incommensurate double-wall carbon nanotubes (i-DWNTs), which further highlights the uniqueness of periodicity as well as stereoisomerism of the incommensurate pairs^3,4^. Thus, for a helical nanotube specified with a unique (*n*,*m*) chiral index (*n* ≠ *m*)^5^, there exist (*P*)- and (*M*)-enantiomers^6^, and in i-DWNT combining small-bore and large-bore (*n*,*m*)-nanotubes, four stereoisomers of two diastereomers with two enantiomer pairs emerge (Fig. 1). Although the i-DWNT systems can dramatically diversify the periodicity and stereoisomerism of nanotubes, structural information with atomic precision cannot be defined with infinite carbon nanotubes due to the highly heterogeneous structures of nanotube mixtures. Recently, we introduced a molecular version of i-DWNTs by adopting (9,6)-[3]cyclo-3,11-dibenzochrysenylene (**[3]C**^**db**^**C**)^7^ as an inner tube and (20,4)-[4]cyclo-3,11-fulminenylene (**[4]CF**) as an outer tube (Fig. 1)^8^. Stereoisomeric pairs of (*P*)- and (*M*)-isomers for inner and outer tubes were examined for the first time as a molecular version of i-DWNT complexes, which revealed that heterohelical combinations of (*P*):(*M*)-pairs [(*P*)⊃(*M*) and (*M*)⊃(*P*)] were preferred over homohelical combinations of (*P*):(*P*)- and (*M*):(*M*)-pairs [(*P*)⊃(*P*) and (*M*)⊃(*M*)]. Although the spontaneous assembly of i-DWNT complexes proceeded in a stereoselective manner to suppress structural diversity in this first instance, further structural investigations of nanotube structures are necessary to deepen our understanding of the structural chemistry of i-DWNTs. In addition, as elaborated below, the i-DWNT complex of this first instance relied on a fortuitous finding of matched DWNT pairs, which, in turn, showed the presence of synthetic challenges of rational, chemical design of i-DWNT complexes. In this study, we designed a large-bore nanotube molecule for the outer tube of the i-DWNT complex by exploring a target-oriented strategy. The i-DWNT assembly of helical nanotube molecules was successfully obtained, which showed that the handedness of nanotubes was not a sole factor that determined the stereoselectivity of bilayer formation.

## Results and discussion

### Molecular design

In our previous study on molecular i-DWNT complexes, we obtained the incommensurate pair of (*P*)-**[4]CF**⊃(*M*)-**[3]C**^**db**^**C** and (*M*)-**[4]CF**⊃(*P*)-**[3]C**^**db**^**C** by focusing on [*n*]phenacenes as a parent arylene panel for belt-persistent cycloarylenes (Fig. 1)^8,9^. It was merely fortuitous that we found **[4]CF** as a large-bore outer tube to encapsulate a small-bore tube of **[3]C**^**db**^**C**, and the design was neither strategic nor rational. For this study, we first scrutinized cycloarylene structures to enumerate synthetically accessible helical nanotube molecules in full, which will be also instructive for further target-oriented syntheses of nanotube molecules in the future^10–14^.

**Fig. 2:**
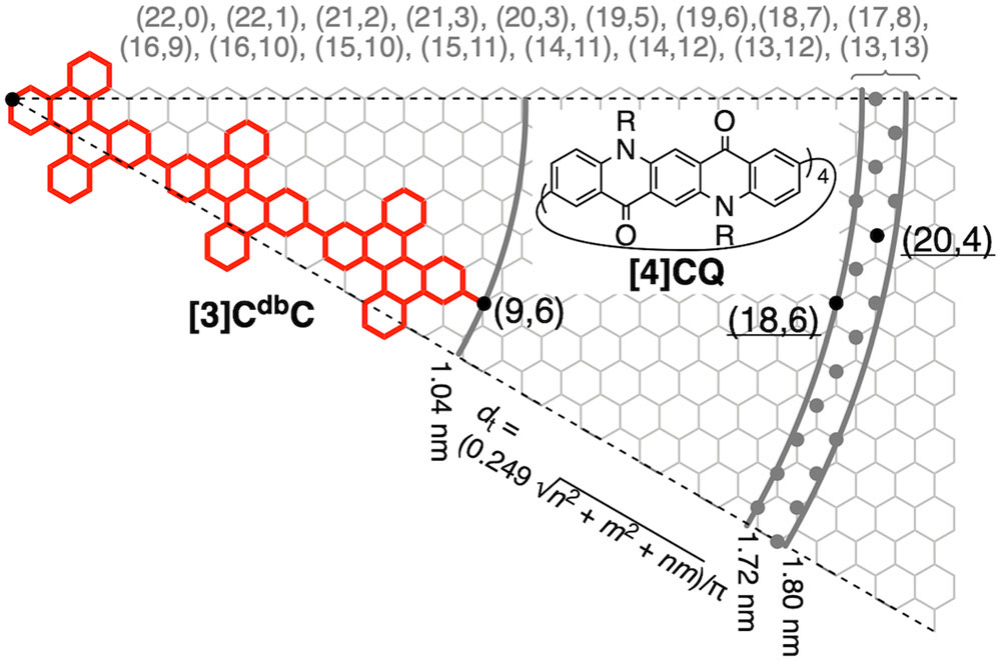
Design of large-bore nanotube molecules to encapsulate [3]C^db^C with a chiral index of (9,6). In a range of 1.72-1.80 nm, there were 19 candidates, and by enumerating arylene panels for the synthesis (Supplementary Figs. 1–4), two chiral indices, i.e., (20,4) and (18,6), remained synthetically accessible candidates. By examining the chemical structures of these chiral indices, we found **[4]CQ** to be an ideal target for large-bore nanotubes.

First, as the inner tube, we decided to adopt an identical molecule, **[3]C**^**db**^**C**, with a chiral index of (9,6) due to its small diameter (*d*_t_) of 1.04 nm (Fig. 2)^7^. Considering the van der Waals radius of carbon atoms, we should then set the diameters of the outer target in the range of 1.72–1.80 nm^8^. In this range, as shown in the map in Fig. 2, 19 chiral indices were found to be chiral vectors of appropriate nanotube molecules with ideal diameters. In this study, an atlas of synthetically accessible nanotubes via tetrameric macrocyclization of arylenes was completed by expanding a previous list of *D*_4_-symmetric molecules^15^, which allowed us to find suitable synthetic targets. Thus, with the 19 chiral indices of candidates in hand, we inspected the atlas shown in Supplementary Figs. 2–4 and found two chiral indices, i.e., (20,4) and (18,6), in the maps of accessible cycloarylenes with *D*_4_ and *C*_2_ symmetry. Examining the structural details of these cycloarylenes, we found [4]cyclo-2,9-pentacenylene (“AAAA” with panel ID = 17, Supplementary Fig. 2) to be a feasible target with a minimum number of hexagons on the panel. Furthermore, we decided to avoid intact [4]cyclo-2,9-pentacenylene as the target because such large acenes are prone to oxidative degradation, particularly in the presence of structural constraints of curved structures^15^. Consequently, we decided to adopt preoxidized pentacene skeletons as an alternative panel and were delighted to find quinacridone as a doped congener of pentacene (Fig. 2). The quinacridone panel possessed two types of heteroatoms, i.e., N and O, installed in the pentacene skeleton, which should be stabilized against possible oxidation. More importantly, quinacridone is abundantly available as a pigment known as Pigment Violet 19 and can be readily converted to a 2,9-dibrominated congener^16^. Thus, a cyclic tetramer of quinacridone, i.e., [4]cyclo-2,9-quinacridonylene (**[4]CQ**), has become as a synthetic target as a large-bore nanotube molecule in this study.

### Synthesis of [4]CQ

**Fig. 3:**
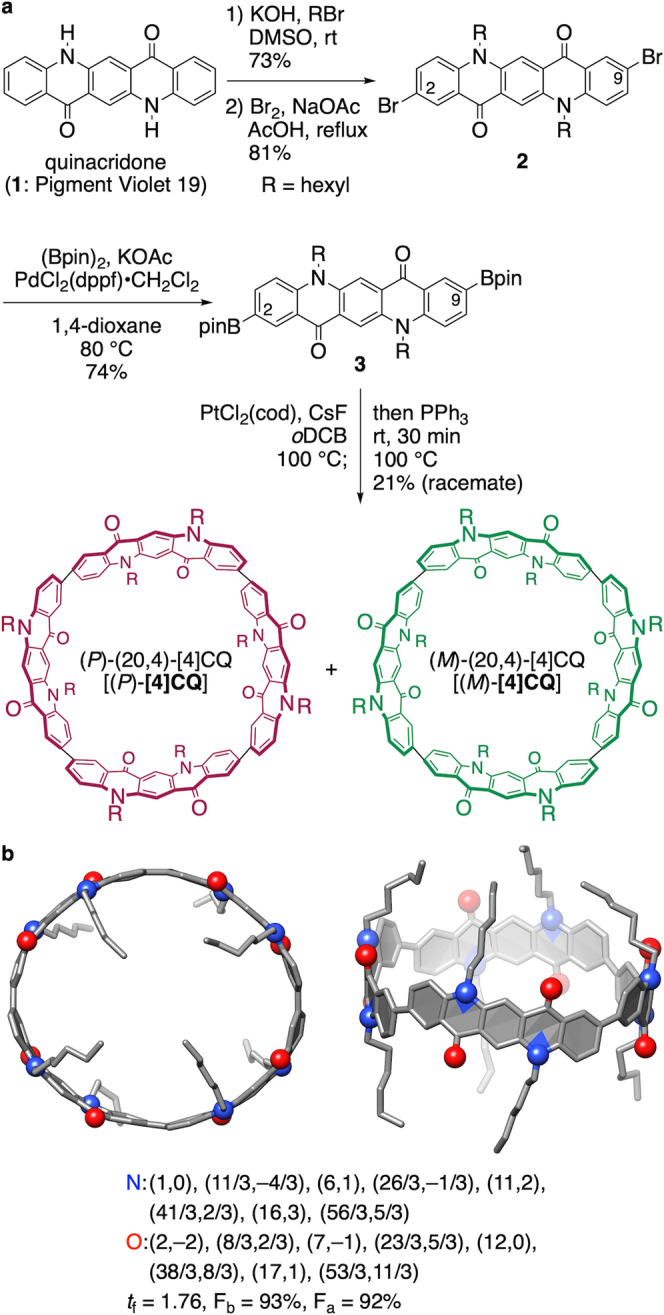
N,O-Doped cylindrical cycloarylene, [4]CQ. **a** Synthesis. **b** Crystal structure. Among two similar yet independent structures, a representative structure is shown. Solvent molecules and hydrogen atoms were omitted for clarity. See Supplementary Fig. 5 for details.

We devised a robust synthetic route to **[4]CQ** from quinacridone (**1**; Pigment Violet 19) and synthesized belt-persistent cylindrical cycloarylenes with a chiral index of (20,4). Thus, boryl linkers at the 2,9-positions in quinacridone were introduced by multistep transformations via N-alkylation, 2,9-selective bromination and Miyaura borylation (Fig. 3). The final cyclization was performed by adopting Yamago’s method with our boryl-linker modification for the Pt-mediated tetrameric macrocyclization. Owing to panel orientations in the cycloarylene structures, four diastereomers were possible for this macrocyclization^9,10,17^, and we were delighted to obtain desired (20,4)-isomers (hereafter denoted as **[4]CQ**) in a selective manner as a single diastereomer with AAAA/BBBB panel orientations. The (20,4)-structure of **[4]CQ** was fully established by single-crystal X-ray diffraction analyses. Thus, the homogeneous panel orientations of AAAA/BBBB suggested by spectroscopic analyses were confirmed by the crystal structure. As suggested by the intrinsic *D*_4_-symmetry of **[4]CQ**, a pair of enantiomers was present with **[4]CQ** in the racemic crystal, and a representative (*P*)-isomer is shown in Fig. 3b. The molecular structure was deformed in an oval shape with major and minor diameters of 1.9 and 1.7 nm, respectively. Heteroatoms of nitrogen and oxygen were periodically located on the cylindrical structure of the (20,4)-nanotube, and their atomically precise positions on the graphitic sheet were also specified using vectorial nomenclature, as shown in Fig. 3b^18^, (https://physorg.chem.s.u-tokyo.ac.jp/applet/defect/).

### Chiral resolution

**Fig. 4:**
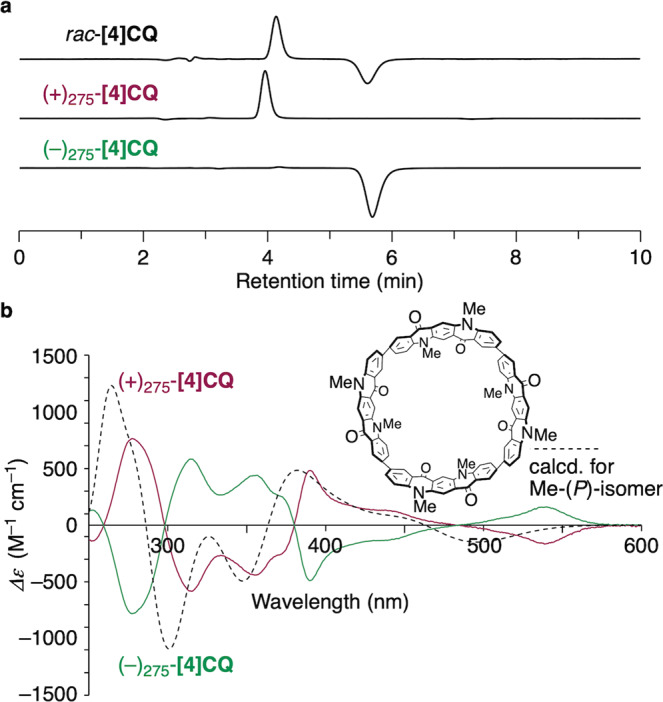
Chiral resolution of [4]CQ. **a** HPLC chromatograms before and after being resolved. HPLC conditions: eluent = 50% CH_3_OH/CH_2_Cl_2_, temperature = 40 °C, flow rate = 1 mL/min. **b** CD spectra of (+)_275_-**[4]CQ** (CH_2_Cl_2_, 298 K, 3.02 × 10^–6^ M) and (–)_275_-**[4]CQ** (CH_2_Cl_2_, 298 K, 3.97 × 10^–6^ M). The theoretical CD spectrum was obtained by TD DFT calculations (B3LYP/6-31 G(d,p)) for the (*P*)-isomer of methyl-substituted **[4]CQ** as the model.

The large-bore **[4]CQ** molecules were segments of (20,4)-helical nanotubes and possessed chirality originating from the helicity of atomic networks in the cylindrical conjugated systems. We were delighted to find that a method developed for chiral resolution of hydrocarbon cylindrical molecules was applicable to chiral resolution of this N,O-doped cylindrical molecules. Thus, when a racemate of **[4]CQ** was injected on columns of cholesterol-loaded silica gels (COSMOSIL Cholester) under high-performance liquid chromatography (HPLC) conditions with 50%-methanol/dichloromethane eluent, two enantiomers were separated: one isomer, (+)_275_-**[4]CQ**, appeared at a retention time of 4.1 min, and its enantiomer, (–)_275_-**[4]CQ**, appeared at 5.6 min (Fig. 4a). With the separated enantiomers in hand, we recorded CD spectra of these isomers to find mirror-image spectra, as shown in Fig. 4b. We then performed time-dependent (TD) DFT calculations for the spectral simulation using (*P*)-**[4]CQ** with methyl substituents as a model^6^. The CD spectrum of the (*P*)-isomer matched (+)_275_-**[4]CQ**, showing that (+)_275_-**[4]CQ** should be assigned as (*P*) for the helicity of atomic arrangements in cylindrical conjugated systems.

### Assembly of i-DWNT

**Fig. 5:**
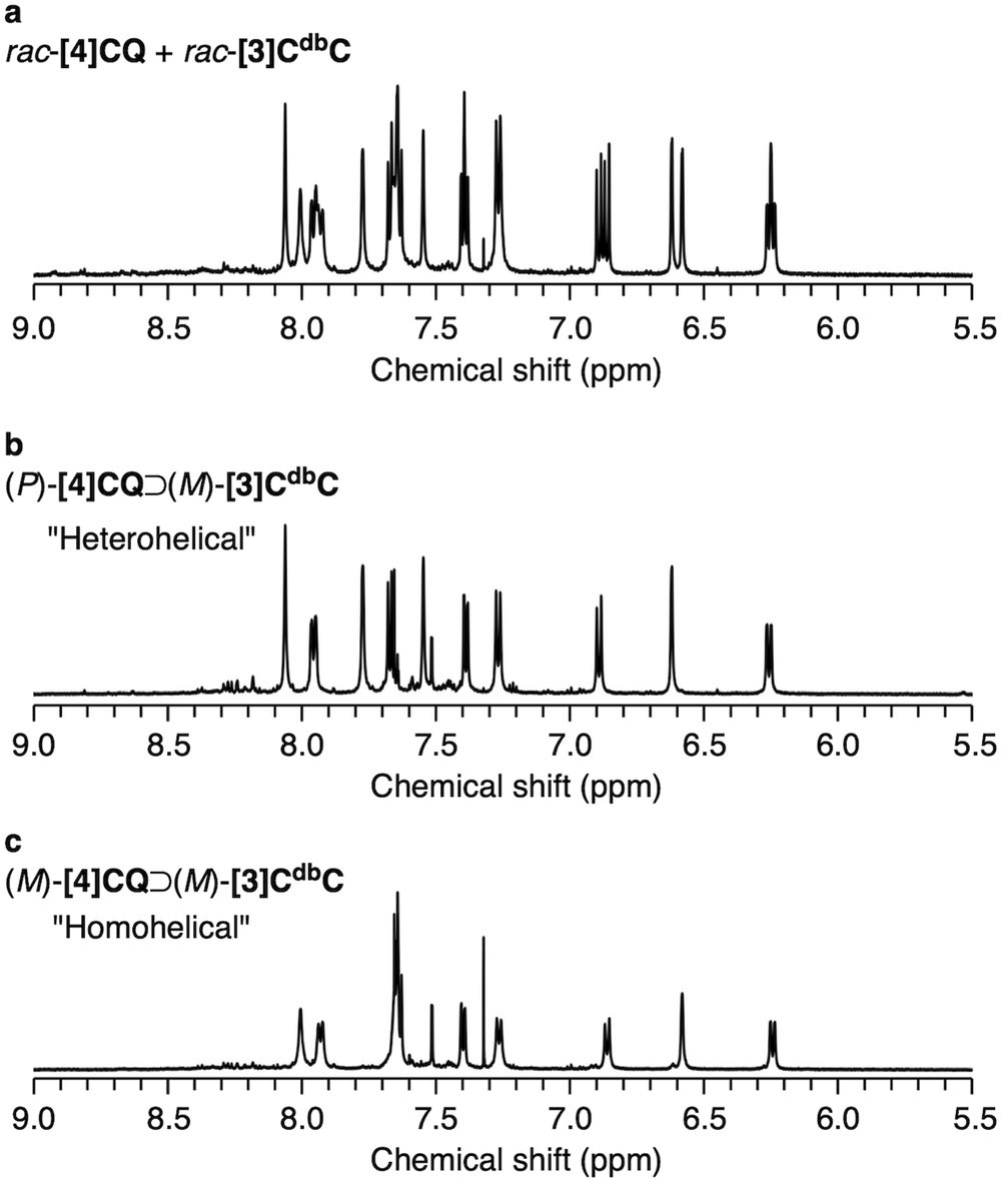
^1^H NMR spectra of i-DWNT complexes between [4]CQ and [3]C^db^C. Spectra were recorded in CD_2_Cl_2_ (1.0 mM, 600 MHz, 298 K). **a** ^1^H NMR spectrum of *rac*-**[4]CQ** + *rac*-**[3]C**^**db**^**C**. **b** Heterohelical combination of (*P*):(*M*)-nanotubes. **c** Homohelical combination of (*M*):(*M*)-nanotubes.

The formation of the i-DWNT complexes between **[4]CQ** and **[3]C**^**db**^**C** was then confirmed by ^1^H NMR spectra. An unexpected spectrum was first obtained, when we analysed a mixture of *rac*-**[4]CQ** and *rac*-**[3]C**^**db**^**C**. In a previous study, when **[4]CF** with 6 aromatic ^1^H resonances was mixed with **[3]C**^**db**^**C** with 6 aromatic ^1^H resonances, we observed 12 aromatic ^1^H resonances (i.e., 12 = 6 + 6), which resulted from the selective formation of a single diastereomer of **[4]CF**⊃**[3]C**^**db**^**C** complexes from (*P*):(*M*)- and (*M*):(*P*)-heterohelical combinations^8^. In this study, however, using **[4]CQ** with 4 aromatic ^1^H resonances, 20 aromatic ^1^H resonances appeared upon mixing with **[3]C**^**db**^**C** (i.e., 20 > 6 + 4) (Fig. 5a). When the ratio of inner and outer nanotubes deviated from an equimolar amount, we separately observed complexed and uncomplexed species as two independent species (Supplementary Figs. 7 and 8). The observation showed that the 20-resonance spectrum corresponded to the i-DWNT complexes due to the slow in-and-out exchange equilibrium^8^. Among a few possible scenarios to explain the observation of increased aromatic resonances, nonstereoselective formation of i-DWNT complexes was most plausible: by mixing *rac*-**[4]CQ** and *rac*-**[3]C**^**db**^**C**, the i-DWNT formation proceeded in a nonselective manner to afford four diastereomers from heterohelical [(*P*)⊃(*M*) and (*M*)⊃(*P*)] and homohelical [(*P*)⊃(*P*) and (*M*)⊃(*M*)]combinations (see also Fig. 1).

The formation of i-DWNT from the nonstereoselective complexation was confirmed by the preparation of two diastereomeric complexes in separate experiments. Thus, when we recorded the ^1^H NMR spectrum by mixing the heterohelical combination of (*P*)-**[4]CQ** and (*M*)-**[3]C**^**db**^**C**, a spectrum with 10 aromatic resonances was obtained, as shown in Fig. 5b. Likewise, a homohelical mixture of (*M*)-**[4]CQ** and (*M*)-**[3]C**^**db**^**C** gave another spectrum with 10 aromatic resonances (Fig. 5c). By comparing these ^1^H NMR spectra shown in Fig. 5, the spectrum obtained from the racemate mixture should be best interpreted as a sum of the spectra of (*P*)-**[4]CQ**⊃(*M*)-**[3]C**^**db**^**C** and (*M*)-**[4]CQ**⊃(*M*)-**[3]C**^**db**^**C**. The obtained result confirmed the nonstereoselective formation of i-DWNT complexes of **[4]CQ**⊃**[3]C**^**db**^**C** from the racemates.

### Association constants of i-DWNT assembly

**Fig. 6:**
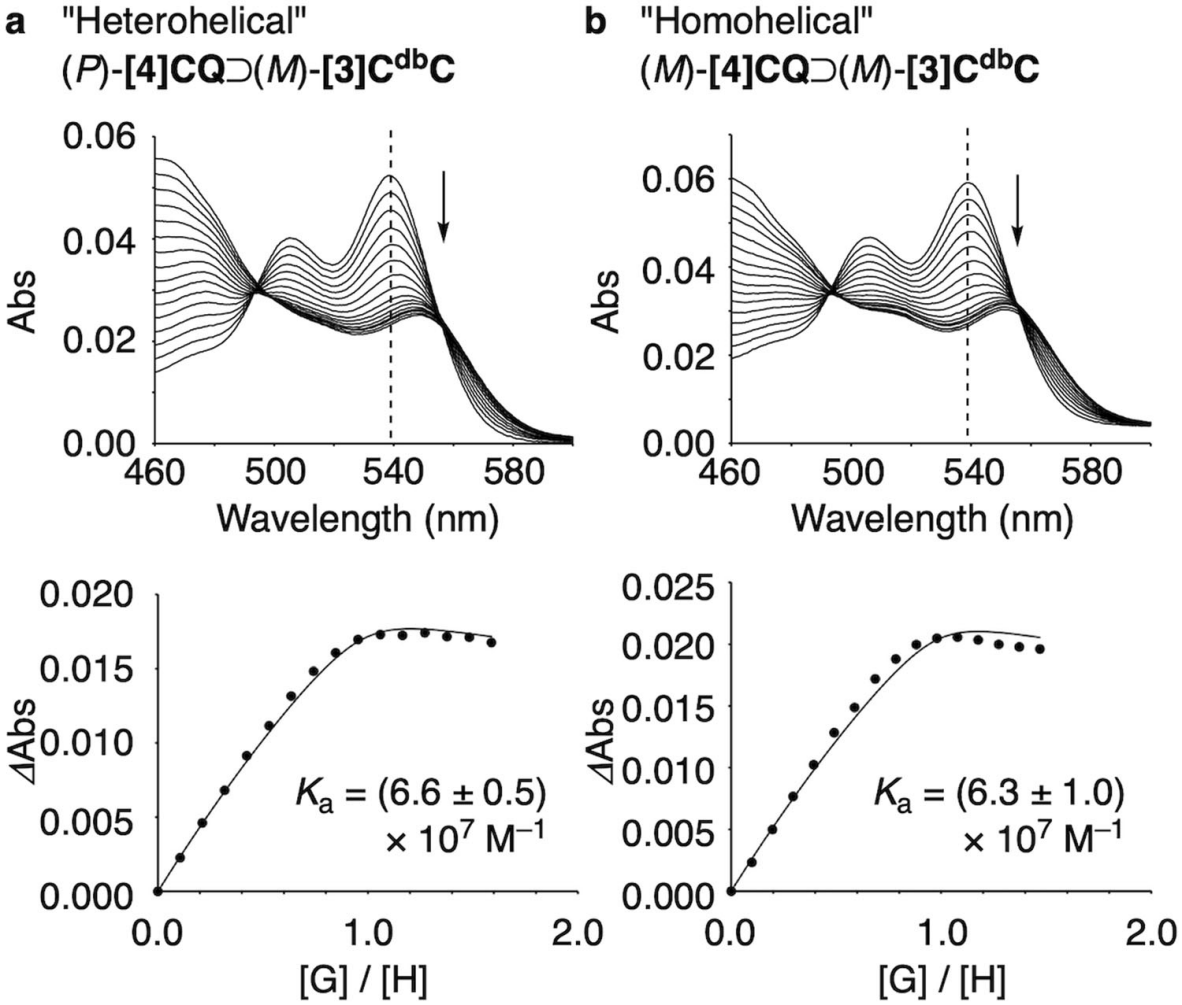
Titration experiments to determine the association constants of i-DWNT complexes. Experiments were performed in triplicate, and representative data are shown. **a** UV–vis data. **b** Fitting curves of the absorbance changes (*Δ*Abs) at 539 nm for *K*_a_ determination. See “Methods” and Supplementary Data 4 for details.

By determining the association constants for the formation of heterohelical and homohelical i-DWNT complexes, we revealed the fundamental parameters of the association thermodynamics. As shown in Fig. 6, upon addition of small-bore **[3]C**^**db**^**C**, we observed redshifts with absorption of **[4]CQ**, and using characteristic absorbance of **[4]CQ** at 539 nm, we determined the association constants (see “Methods” for details)^19^. Thus, the association constant (*K*_a_) of the heterohelical (*P*)-**[4]CQ**⊃(*M*)-**[3]C**^**db**^**C** complex was determined to be (6.6 ± 0.5) × 10^7 ^M^–1^, whereas that of the homohelical (*M*)-**[4]CQ**⊃(*M*)-**[3]C**^**db**^**C** complex was elucidated to be (6.3 ± 1.0) × 10^7 ^M^–1^. The Gibbs free energy gains (*ΔG*) for the association were thus determined as –10.7 ± 0.0 kcal mol^–1^ for (*P*)-**[4]CQ**⊃(*M*)-**[3]C**^**db**^**C** and –10.6 ± 0.1 kcal mol^–1^ for (*M*)-**[4]CQ**⊃(*M*)-**[3]C**^**db**^**C** (298 K). The *K*_a_ values for both complexes were comparable and were as high as ~10^7 ^M^–1^, which clarified the origin of the observations of both forms from a mixture of racemates (*cf*. Fig. 5a). Interestingly, the association constants with this large-bore **[4]CQ** nanotubes were much higher than those of previous large-bore **[4]CF** nanotubes with an identical chiral index of (20,4) to record two to three orders of magnitude larger *K*_a_ values^8^.

### Crystal structures of i-DWNT

**Fig. 7:**
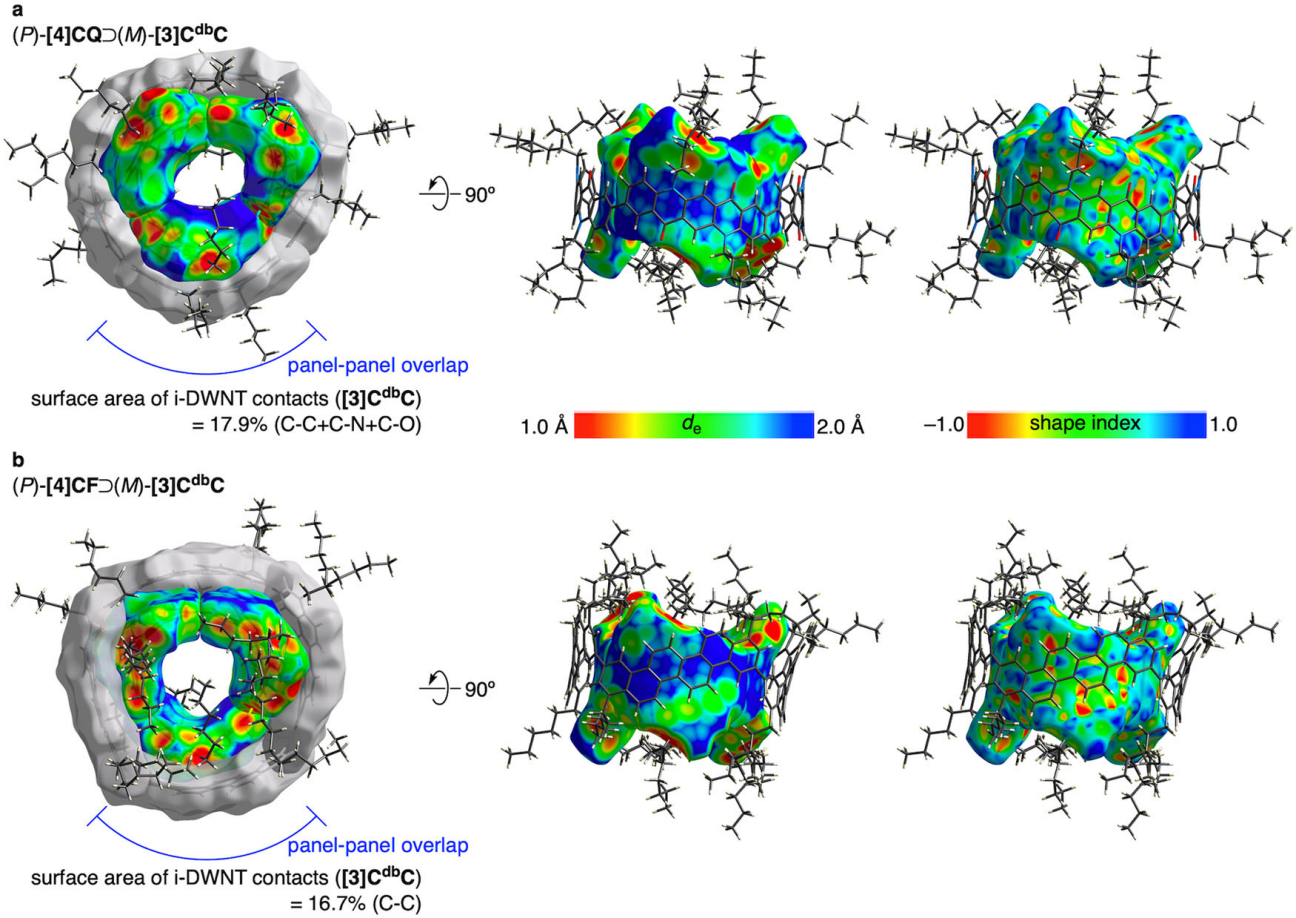
Crystal structures of i-DWNT complexes. The Hirshfeld surfaces were determined for atoms involved in the π-conjugate systems and were divided into arylenes to clarify the locations of the panels. See Supplementary Fig. 10 for details of panel-panel overlaps. Solvent molecules were omitted for clarity. **a** (*P*)-**[4]CQ**⊃(*M*)-**[3]C**^**db**^**C**. **b** (*P*)-**[4]CF**⊃(*M*)-**[3]C**^**db**^**C**. The CIF data were taken from the previous work (CCDC 2034189)^8^.

Detailed structural comparisons of i-DWNT complexes with **[4]CQ** and **[4]CF** became feasible because we obtained a single crystal of the heterohelical (*P*)-**[4]CQ**⊃(*M*)-**[3]C**^**db**^**C** complex. Thus, as shown in Fig. 7a, the molecular structure of the (*P*)-**[4]CQ**⊃(*M*)-**[3]C**^**db**^**C** complex possessed an ideal i-DWNT structure with coaxial alignment, which was similar to that of the previous (*P*)-**[4]CF**⊃(*M*)-**[3]C**^**db**^**C** complex (Fig. 7b). The small-bore nanotube at the inner site was identical [i.e., (*M*)-**[3]C**^**db**^**C**] and served as a probe for detailed structural comparisons. As highlighted in Fig. 7, one of the arylene panels of the outer tube covered one dibenzochrysenylene panel of the inner tube for both structures, which provided an ideal segment for the structural comparisons. The Hirshfeld surfaces of **[3]C**^**db**^**C** were then mapped with intermolecular contacts for structural analyses^20^. The *d*_e_ mapping shows distances from the surface to the external atoms of the outer tubes. Although the chiral index and the geometric diameter (*d*_t_) were identical for **[4]CQ** and **[4]CF**, the skeletal network of the arylene panels was different and resulted in different mapping of contact areas. The shape index that revealed the convex and concave areas of the surface also differed between the two i-DWNT complexes. However, the surface areas for π-contacts between inner and outer tubes were similar in these two i-DWNT complexes. Thus, when we measured the surface areas of **[3]C**^**db**^**C** for π-contacts in (*P*)-**[4]CQ**⊃(*M*)-**[3]C**^**db**^**C**, a value of 17.9% was obtained. When we measured the π-contact areas of **[3]C**^**db**^**C** in (*P*)-**[4]CF**⊃(*M*)-**[3]C**^**db**^**C**, a value of 16.7% was obtained. The obtained results showed that the overlapping areas were similar in the two complexes despite different arrangements of the atomic networks.

### Thermodynamics of i-DWNT assembly

**Fig. 8:**
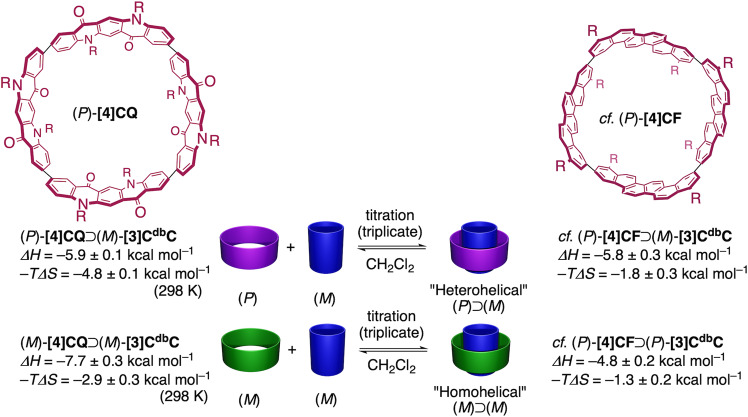
Thermodynamics of i-DWNT assembly. Data with (*P*)-**[4]CF** were taken from the previous work^8^.

Finally, isothermal titration calorimetry (ITC) allowed us to reveal the in-depth thermodynamics of i-DWNT formation^21^. Because the associations of this i-DWNT complex were extremely exothermic processes with *K*_a_ values of >10^7 ^M^–1^, the ITC curve were inappropriate for fitting analyses to derive *K*_a_ determinations (see Supplementary Data 4)^22,23^. Nonetheless, the titration allowed us to determine enthalpy gains (*ΔH*) for the association via measurement of the exchanging heat. Using the *ΔG* values from UV-vis titration experiments, we can complete the thermodynamic parameters including the entropy changes (Δ*S*) for the associations. Thus, the thermodynamic parameters for the association of the heterohelical (*P*)-**[4]CQ**⊃(*M*)-**[3]C**^**db**^**C** complex were *ΔH* = –5.9 ± 0.1 kcal mol^–1^ and –*T*Δ*S* = –4.8 ± 0.1 kcal mol^–1^, and those for the homohelical (*M*)-**[4]CQ**⊃(*M*)-**[3]C**^**db**^**C** complex were Δ*H* = –7.7 ± 0.3 kcal mol^–1^ and –*TΔS* = –2.9 ± 0.3 kcal mol^–1^ (Fig. 8). Although the *K*_a_ values for the heterohelical complexes with **[4]CQ** and **[4]CF** differed considerably (see above), the difference in the Δ*H* values for these complexes was small (*ΔΔH* ~ 0.1 kcal mol^–1^). The obtained result is qualitatively consistent with the similar surface area of π-contacts determined by the Hirshfeld analyses for these complexes (17.9% and 16.7%; see above). The DFT calculations of i-DWNT complexes reproduced the preference of stereoisomeric combinations (homohelical > heterohelical for **[4]CQ**; heterohelical > homohelical for **[4]CF**) (Supplementary Data 5). Although the calculated energies for association matched finely with Δ*H* values^24,25^, differences between **[4]CQ** systems and **[4]CF** systems were so subtle (~1 kcal mol^–1^) that should be difficult to be reproduced by theoretical calculations. The present system should also be interesting subjects to be investigated by state-of-the-art theoretical and physical investigations, because of the presence of rapid dynamic rotational motions (Supplementary Fig. 9) on top of due considerations of appropriate theoretical models^26,27^. The *ΔG* difference between these complexes was largely ascribed to the entropy (–*T*Δ*S*). Similarly, favourable entropy contributions for the association (–*T*Δ*S* < 0) were observed with complexes with large curved π-systems in preceding studies of nanocarbon molecules^22,28–31^, which was most likely due to desolvation of solvent molecules from the curved surface. Therefore, we believe that the large favourable entropy gain with **[4]CQ** (–*T*Δ*S* ~ 4 kcal mol^–1^) should be best explained by changes in the solvation in the presence of N,O-heteroatoms. In contrast, although the *K*_a_ values were similar between diastereomeric complexes of (*P*)-**[4]CQ**⊃(*M*)-**[3]C**^**db**^**C** and (*M*)-**[4]CQ**⊃(*M*)-**[3]C**^**db**^**C** (~10^7 ^M^–1^), the contributing factors showed subtle differences of ΔΔ*H* ~ 1.8 kcal mol^–1^ and ΔΔ*S* ~ 6 cal mol^–1^ K^–1^. Presently, we do not understand the origins of these differences that show enthalpy-entropy compensation trends^32–35^. Compensation can involve dynamic behaviours including the solvation and rolling dynamics of i-DWNT^36^, which should be further investigated in the future.

## Conclusion

Target-oriented design of cylindrical cycloarylenes as segmental models of helical carbon nanotubes was devised, and a large-bore cylindrical molecule (**[4]CQ**) was synthesized from an abundantly available pigment. The large-bore molecule was obtained in a stereoselective manner as a racemate, and owing to the heteroatoms embedded in the arylene panels, the molecule should serve as a segmental model of N,O-doped helical carbon nanotubes with a chiral index of (20,4). The large-bore cylinder encapsulated a small-bore cylindrical molecule (**[3]C**^**db**^**C**) to afford a molecular version of i-DWNTs. When compared with a preceding hydrocarbon outer tube (**[4]CF**), the outer tube in this study increased the affinity to the small-bore molecule to result in a larger association constant of 10^7 ^M^–1^. The assembly of i-DWNT complexes proceeded in a nonstereoselective manner, which was also in contrast to the previous case with **[4]CF** that had unfavourable i-DWNT complexes. These observations show that further studies of other structural variants are necessary to clarify the mechanism and origins of the stereoselectivity of i-DWNT assembly. Crystallographic analyses and thermodynamic studies revealed favourable enthalpy contributions via π-contacts for the i-DWNT assembly and important roles of subtle changes in the favourable entropy contributions that tweaked the affinity between inner and outer tubes. We hope that this study will stimulate further studies on i-DWNT complexes as well as heteroatom-doped nanotube molecules in the future^37–40^.

## Methods

### Physical techniques

Flash silica gel column chromatography was performed on silica gel 60 N (spherical and neutral gel, 40–50 μm, Kanto). Gel permeation chromatography (GPC) was performed on an LC-5060 with JAIGEL 2HR-40 and 2.5 HR-40 polystyrene column (Japan Analytical Industry) with chloroform as the eluent. The purity of the target molecule was confirmed by high-performance liquid chromatography (HPLC) with two different columns (COSMOSIL πNAP, 4.6ϕ × 250 mm and COSMOSIL BuckyPrep, 4.6ϕ ×  250 mm, Nacalai Tesque). Chromatographic chiral resolution was performed with chiral columns (COSMOSIL Cholester, Nacalai Tesque) using a 4.6ϕ × 250 mm column for analytical scales and a 20ϕ × 250 mm column for preparative scales. The analytical HPLC was performed at 40 °C in a column oven (CO2060PLUS, JASCO) under detection of UV–vis absorption (MD2018PLUS, JASCO) and CD (CD2095PLUS, JASCO) with a flow rate of 1.0 mL/min. The preparative HPLC was performed at ambient temperature under UV-vis detection (UV2075PLUS, JASCO) with a flow rate of 18 mL/min. ITC analyses were performed on a microcalorimeter (MicroCal iTC200, Malvern). High resolution mass spectra (HRMS) were recorded on an autoflex speed device (Bruker Daltonics) using the matrix-assisted laser desorption ionization (MALDI) method with pyrene or dithranol as a matrix under reflector positive mode. UV–vis and CD spectra were recorded on a V-670 and J-1500 instruments (JASCO) with a temperature controller to adjust the temperature to 25 °C. Nuclear magnetic resonance (NMR) spectra were recorded on RESONANCE JNM-ECA 600 II equipped with the UltraCOOL UC5AT probe (JEOL). Chemical shift values were given with respect to internal CHCl_3_ (7.26) and CHDCl_2_ (5.32) for ^1^H NMR spectra and CDCl_3_ (77.16) and for ^13^C NMR spectra. Methyl (CH_3_), methylene (CH_2_) and methine (CH) signals in ^13^C NMR spectra were assigned by DEPT spectra.

### Materials

Anhydrous THF (stabilizer-free) was purified by a solvent purification system (GlassContour) equipped with columns of activated alumina and supported copper catalyst (Q-5)^41^, and *o*DCB was dried with 4 A molecular sieves before use. Small-bore tubes, (*P*)- and (*M*)-**[3]C**^**db**^**C**, were prepared and purified by methods reported in the literature^7^. For the experiments with **[3]C**^**db**^**C**, the quantity of the compound was determined using the molar absorption coefficients (*ε*_412_ = 1.42 × 10^5 ^M^–1^ cm^–1^) derived from specimens with combustion elemental analysis data. PtCl_2_(cod) was prepared according to the reported procedure^42^. All other chemicals were of reagent grade and used without any further purification. The reactions were performed under N_2_ atmosphere, unless otherwise noted.

### Atlas of synthetically accessible chiral indices

An atlas of synthetically accessible chiral indices was made in this study. For arylene panels with ≤7 hexagon arrays, cylindrical cycloarylenes accessible via tetrameric macrocyclization were enumerated and listed in maps. See Supplementary Methods and Supplementary Figs. 1–4 for details.

### Synthesis of [4]CQ

In a 100-mL three-necked round bottom flask, CsF (3.03 g, 19.9 mmol), **3** (487 mg, 0.665 mmol) PtCl_2_(cod) (249 mg, 0.665 mmol) and *o*DCB (33 mL) were mixed. The mixture was stirred at 100 °C in the dark for 4 h. After the mixture was cooled to ambient temperature, PPh_3_ (1.75 g, 6.68 mmol) was added. The mixture was stirred in the dark for 30 min at ambient temperature and for 24 h at 100 °C. Methanol (250 mL) was added to the mixture, and the precipitate was collected by filtration. The precipitate was dissolved in CHCl_3_ (180 mL), and the insoluble materials were removed by filtration. The crude material was passed through a pad of alumina (eluent: CHCl_3_) and purified by GPC (columns: JAIGEL 2HR-40 and 2.5 HR-40, eluent: CHCl_3_) to afford **[4]CQ** as a racemic mixture of (20,4)-isomers of “AAAA” panel orientations in 21% yield (68.2 mg, 35.6 µmol). The purity was confirmed by analytical HPLC performed with two different columns (Supplementary Data 1). The stereochemistry and chiral index of [4]CQ were determined by a method adopted in a previous study^9^. Thus, NMR spectra assigned the symmetry of either *D*_4_ or *D*_2d_, and the presence of enantiomers (see below) finalized the assignment as *D*_4_-(20,4)-isomers. The assignment was further confirmed by crystallographic analysis (see below). The molar absorption coefficient (*ε*_357_) of *rac*-**[4]CQ** was determined to be 2.02 × 10^5 ^M^–1^ cm^–1^ as follows. A specimen was determined to have a chemical composition of (C_128_H_136_N_8_O_8_)•(CHCl_3_)_0.17_•(H_2_O)_1.74_ using data from combustion elemental analysis of C: 78.42%, H: 7.04%, N: 5.56% and Cl: 0.96%. Using this specimen to prepare three solutions with different concentrations in dichloromethane (0.1032 mg in 50.0 mL = 1.05 × 10^–6^ M, 0.1092 mg in 25.0 mL = 2.22 × 10^–6^ M, 0.1834 mg in 20.0 mL = 4.67 × 10^–6^ M), the UV–vis spectra were recorded to determine the *ε*_357_ value. ^1^H NMR (600 MHz, CDCl_3_): *δ* 8.53 (s, 8H), 8.36 (dd, *J* = 9.4, 2.3 Hz, 8H), 8.32 (d, *J* = 2.3 Hz, 8H), 7.51 (d, *J* = 9.4 Hz, 8H), 4.44 (m, 16H), 2.00-1.80 (m, 16H), 1.56 (m, 16H), 1.44-1.33 (m, 32H), 0.92 (t, *J* = 7.2 Hz, 24H); ^13^C NMR (151 MHz, CDCl_3_): *δ* 179.2, 141.4, 137.4, 131.8, 131.2 (CH), 126.6, 126.4 (CH), 121.2, 115.8 (CH), 113.5 (CH), 47.2 (CH_2_), 31.6 (CH_2_), 26.8 (CH_2_), 26.8 (CH_2_), 22.8 (CH_2_), 14.1 (CH_3_); HRMS (MALDI-TOF) (*m*/*z*): [M + H]^+^ calcd. for C_128_H_137_N_8_O_8_ 1915.0587, found 1915.0570. See Supplementary Data 2 for NMR spectra. UV–vis and CD spectra were recorded in dichloromethane (Supplementary Data 3). See Supplementary Methods for the synthesis of other compounds.

### Crystal structure of *rac*-[4]CQ

See Supplementary Methods, Supplementary Table 1 and Supplementary Data 6.

### Chiral resolution of [4]CQ

See Supplementary Methods and Supplementary Fig. 6.

### Theoretical calculations

See Supplementary Methods and Supplementary Data 5.

### NMR spectra of the i-DWNT assembly

Specimens for i-DWNT spectra were weighed on aluminium pans (6 × 2.5 mm, Lüdi Swiss) using ultramicrobalance (SE2, Sartorius). Each specimen was dissolved in CD_2_Cl_2_ by adding the solvent to the specimen on the pan. The quantities of the specimens and CD_2_Cl_2_ are as follows. Racemate i-DWNT complexes (*rac*-**[4]CQ**⊃*rac*-**[3]C**^**db**^**C**, Fig. 5a): CD_2_Cl_2_ (0.70 mL) was added to a mixture of *rac*-**[4]CQ** (0.6685 mg, 0.3492 µmol), (*P*)-**[3]C**^**db**^**C** (0.2586 mg, 0.1742 µmol) and (*M*)-**[3]C**^**db**^**C** (0.2591 mg, 0.1745 µmol) were added, and the mixture was subjected to NMR analyses (Fig. 5a). Heterohelical i-DWNT complexes [(*P*)-**[4]CQ**⊃(*M*)-**[3]C**^**db**^**C**, Fig. 5b]: A specimen of (*P*)-**[4]CQ** (2.4052 mg, 1.2563 µmol) was dissolved in CD_2_Cl_2_ (1.26 mL), and a specimen of (*M*)-**[3]C**^**db**^**C** (1.8989 mg, 1.2794 µmol) was dissolved in CD_2_Cl_2_ (1.28 mL). Aliquots of 0.30 mL from each solution were mixed, and the mixture was subjected to NMR analyses (Fig. 5b). Homohelical i-DWNT complexes [(*M*)-**[4]CQ**⊃(*M*)-**[3]C**^**db**^**C**, Fig. 5c]: A specimen of (*M*)-**[4]CQ** (1.3602 mg, 0.7105 µmol) was dissolved in CD_2_Cl_2_ (0.71 mL), and a specimen of (*M*)-**[3]C**^**db**^**C** (0.7624 mg, 0.5137 µmol) was dissolved in CD_2_Cl_2_ (0.51 mL). Aliquots of 0.30 mL from each solution were mixed, and the mixture was subjected to NMR analyses (Fig. 5c). See Supplementary Methods and Supplementary Figs. 7–9 for spectral analyses of in-and-exchange processes.

### Titration experiments (UV–vis) for *K*_a_ and *ΔG*

Association constants for i-DWNT **[4]CQ**⊃**[3]C**^**db**^**C** assembly were determined by titration experiments with UV-vis spectra. The concentrations of **[4]CQ** and **[3]C**^**db**^**C** for the titration were determined using the molar absorption coefficients of each compound (**[4]CQ**: *ε*_357_ = 2.02 × 10^5 ^M^–1^ cm^–1^, **[3]C**^**db**^**C**: *ε*_412_ = 1.42 × 10^5 ^M^–1^ cm^–1^). The concentrations of the samples used for the titration were as follows. For the heterohelical (*P*)-**[4]CQ**⊃(*M*)-**[3]C**^**db**^**C** complex: [(*P*)-**[4]CQ**] = 1.49 × 10^–6^ M and [(*M*)-**[3]C**^**db**^**C**]  = 7.87 × 10^–6^ M. For the homohelical (*M*)-**[4]CQ**⊃(*M*)-**[3]C**^**db**^**C** complex: [(*M*)-**[4]CQ**] = 1.64 × 10^–6^ M and [(*M*)-**[3]C**^**db**^**C**] = 8.04 × 10^–6^ M. A typical titration procedure is described for the heterohelical (*P*)-**[4]CQ**⊃(*M*)-**[3]C**^**db**^**C** complex. An aliquot of (*M*)-**[3]C**^**db**^**C** solution in dichloromethane (50 µL) was added to a solution of (*P*)-**[4]CQ** (dichloromethane, 2.50 mL) in a cuvette at the temperature of the spectrometer of 25 °C. After stirring for 1 min, the UV–vis spectrum was recorded. The addition of (*M*)-**[3]C**^**db**^**C** solution was repeated 15 times, and the change in absorbance was recorded (Fig. 6a and Supplementary Data 4). The changes of absorbance at 539 nm (*Δ*Abs) were subjected to fitting analyses to derive the association constant (*K*_a_)^19^. At this wavelength, the molar absorption coefficient of (*M*)-**[3]C**^**db**^**C** was negligible (*ε*_539_ ~ 0 M^–1^ cm^–1^) in contrast to a large value of (*P*)-**[4]CQ** (*ε*_539_ ~ 10^4 ^M^–1^ cm^–1^), which was a precondition for the fitting equation. The titration experiments were performed in triplicate to obtain the mean value with the error in standard deviation. The *K*_a_ values were converted to the Gibbs free energy changes (*ΔG*) by *ΔG* = –*RT* ln *K*_a_. The titration experiments for the homohelical (*M*)-**[4]CQ**⊃(*M*)-**[3]C**^**db**^**C** complex were likewise performed (Fig. 6b and Supplementary Data 4).

### Titration experiments (ITC) for Δ*H*

The enthalpy gains (Δ*H*) for i-DWNT **[4]CQ**⊃**[3]C**^**db**^**C** assembly were determined by isothermal titration calorimetry (ITC) experiments. The concentrations of **[4]CQ** and **[3]C**^**db**^**C** for the titration were determined using the molar absorption coefficients of each compound (**[4]CQ**: *ε*_357_ = 2.02 ×  10^5 ^M^–1^ cm^–1^, **[3]C**^**db**^**C**: *ε*_412_ = 1.42 × 10^5 ^M^–1^ cm^–1^). The concentrations of the samples used for the titration were as follows. For the heterohelical (*P*)-**[4]CQ**⊃(*M*)-**[3]C**^**db**^**C** complex: [(*P*)-**[4]CQ**] = 1.85 × 10^–4^ M and [(*M*)-**[3]C**^**db**^**C**] = 2.50 × 10^–3^ M. For the homohelical (*M*)-**[4]CQ**⊃(*M*)-**[3]C**^**db**^**C** complex: [(*M*)-**[4]CQ**] = 2.16 × 10^–4^ M and [(*M*)-**[3]C**^**db**^**C**] = 2.12 × 10^–3^ M. A typical titration procedure is as follows. A solution of **[3]C**^**db**^**C** was added to a solution of **[4]CQ** in a microcalorimeter cell using the automated titration mode via a syringe. Using the ORIGIN program provided with the instrument, the Δ*H* value was derived. The titration experiments were performed in triplicate to obtain the mean value with the error in standard deviation. By combining the Δ*H* value from ITC and the *ΔG* value from UV–vis, the –Δ*S* value was determined. Based on the principle of the propagation of errors^43^, the error with Δ*S* was calculated by (error Δ*H* ^2^ + error Δ*G*^2^)^1/2^. The titration data are provided in Fig. 8 and Supplementary Data 4).

### Crystal structure of (*P*)-[4]CQ⊃(*M*)-[3]C^db^C

See Supplementary Methods, Supplementary Table 2 and Supplementary Data 7.

### Hirshfeld surface analyses

See Supplementary Methods, Fig. 7 and Supplementary Fig. 10.

## Supplementary information

Supplementary Information

Description of Additional Supplementary Files

Supplementary Data 1

Supplementary Data 2

Supplementary Data 3

Supplementary Data 4

Supplementary Data 5

Supplementary Data 6

Supplementary Data 7

## Data Availability

Supplementary methods and references are available in the Supplementary Information, and Supplementary Data provide additional datasets. Supplementary Data 1: Chromatograms. Supplementary Data 2: NMR spectra. Supplementary Data 3: UV–vis and CD spectra. Supplementary Data 4: Titration data. Supplementary Data 5: Data from DFT calculations. Supplementary Data 6: X-ray crystallographic data of CCDC2204308 in the cif format. Supplementary Data 7: X-ray crystallographic data of CCDC2204309 in the cif format. The X-ray crystallographic coordinates for the structures reported in this study have been deposited at the Cambridge Crystallographic Data Centre (CCDC), under deposition numbers 2204308 and 2204309. These data can be obtained free of charge from The Cambridge Crystallographic Data Centre via www.ccdc.cam.ac.uk/data_request/cif. Other data are available from the corresponding author upon reasonable request.
