# Supplementary material for: Target-oriented design of helical nanotube molecules for rolled incommensurate bilayers
Source: Commun Chem. 2022 Nov 19;5:152. doi: 10.1038/s42004-022-00777-2 (PMC9814558; doi:10.1038/s42004-022-00777-2)
Supplement: Supplementary file 1 — Supplementary Information [file 42004_2022_777_MOESM1_ESM.pdf]

Supplementary Information

Target-oriented design of helical nanotube molecules for rolled incommensurate bilayers

Hiroyuki Isobe,* Yuki Kotani, Taisuke Matsuno, Toshiya M. Fukunaga, Koki Ikemoto

Department of Chemistry, The University of Tokyo, Hongo 7-3-1, Bunkyo-ku, Tokyo 113-0033

Table of Contents

Supplementary Methods 2

Enumeration of nanotube molecules for the target-oriented design 2

Synthesis 7

*Crystal structure of rac-**[4]CQ*** 8

*Chiral resolution of **[4]CQ*** 10

Theoretical calculations 11

NMR spectra of i-DWNT: In-and-out exchange 11

*Crystal structure of (P)-**[4]CQ**⊃(M)-**[3]C^{db}C*** 14

Hirshfeld surface analyses 15

Supplementary References 16

Supplementary Methods

Enumeration of nanotube molecules for the target-oriented design

In a previous study, we enumerated synthetically accessible nanotube molecules under restrictions of the D_4 symmetry (*i.e.*, "AAAA" panel orientations for ≤7-hexagon arylene panels) and listed 163 molecules as candidates.[1] In the present work, a list of synthetically accessible nanotube molecules via four-panel cycloarylenes was expanded, and a list of 652 molecules was completed by considering other panel orientations of "AAAB", "ABAB" and "AABB" orientations. Importantly, corresponding chiral indices of these cylindrical molecules were also enumerated and mapped on a graphene sheet to make concise atlas of accessible nanotube molecules. The accessible indices for "ABAB" and "AABB" were identical and were summarized in one figure. Believing that such atlas should be informative for further explorations of cylindrical molecules including nanobelts, we present the list and maps in Supplementary Figs. 1-4. In short, with the panels shown in Supplementary Fig. 1, synthetically accessible molecules can be found in Supplementary Figs. 2-4. Note that the developed maps shown in Supplementary Figs. 2-4 are created under the conditions of $n \geq m \geq 0$ to be supplemented by *R*/*S*-designations of chirality.[2,3] Note that the atlas covers all the geometrically possible structures without consideration of chemically possible/impossible structures. The chiral indices accessible with cyclo-*para*-phenylenes (CPPs) can be found in a much easier process, because their chiral indices are (n,n) where n stands for the number of phenylene panels involved in the structure. In contrast, cycloarylenes with larger arylene panels can form a diverse range of carbon nanotube molecules shown in the present atlas.

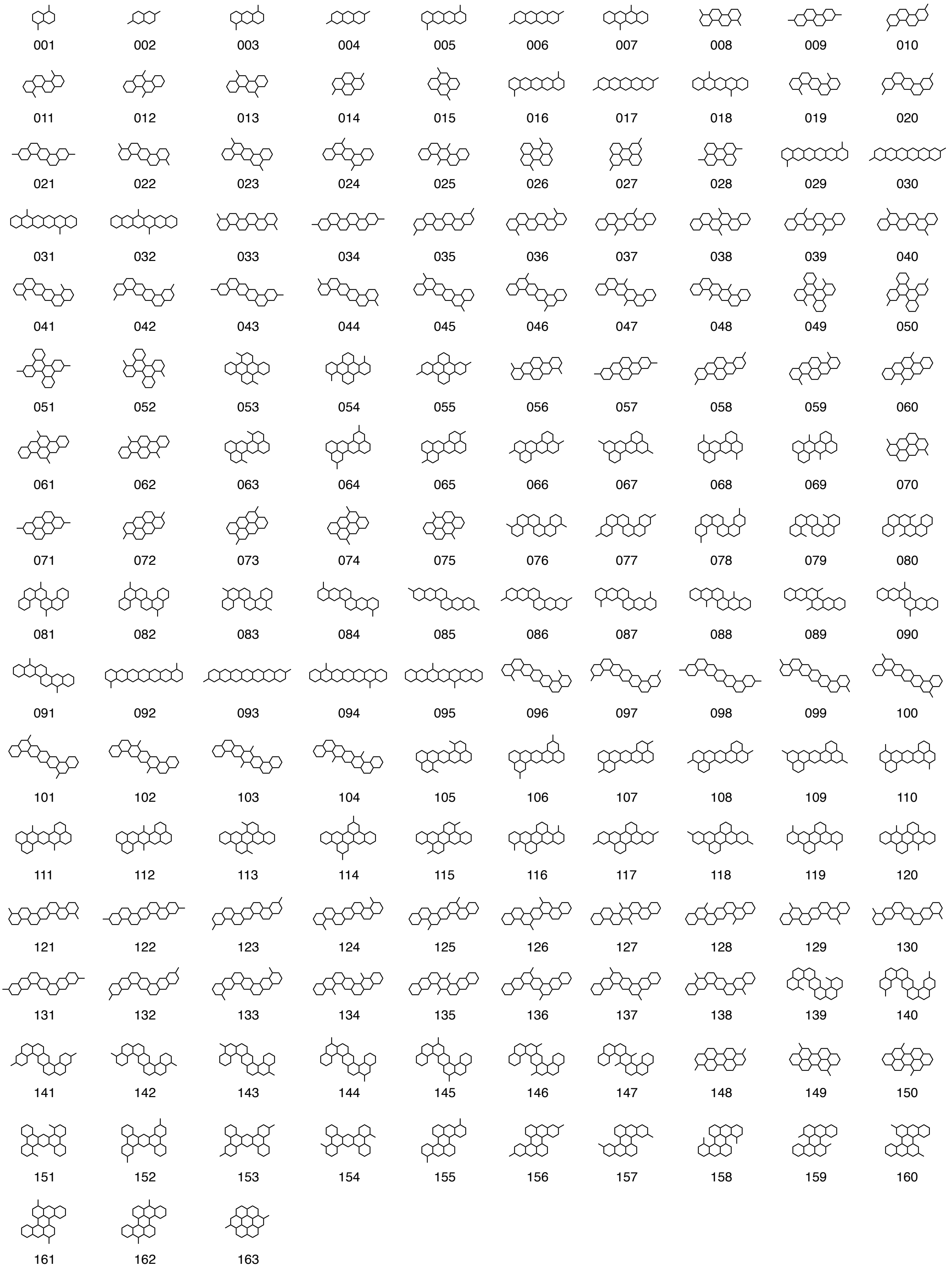

Supplementary Fig. 1. A list of arylene panels (number of hexagons with 2-7) for cylindrical cycloarylenes via tetramerization. See ref. 1 for details.

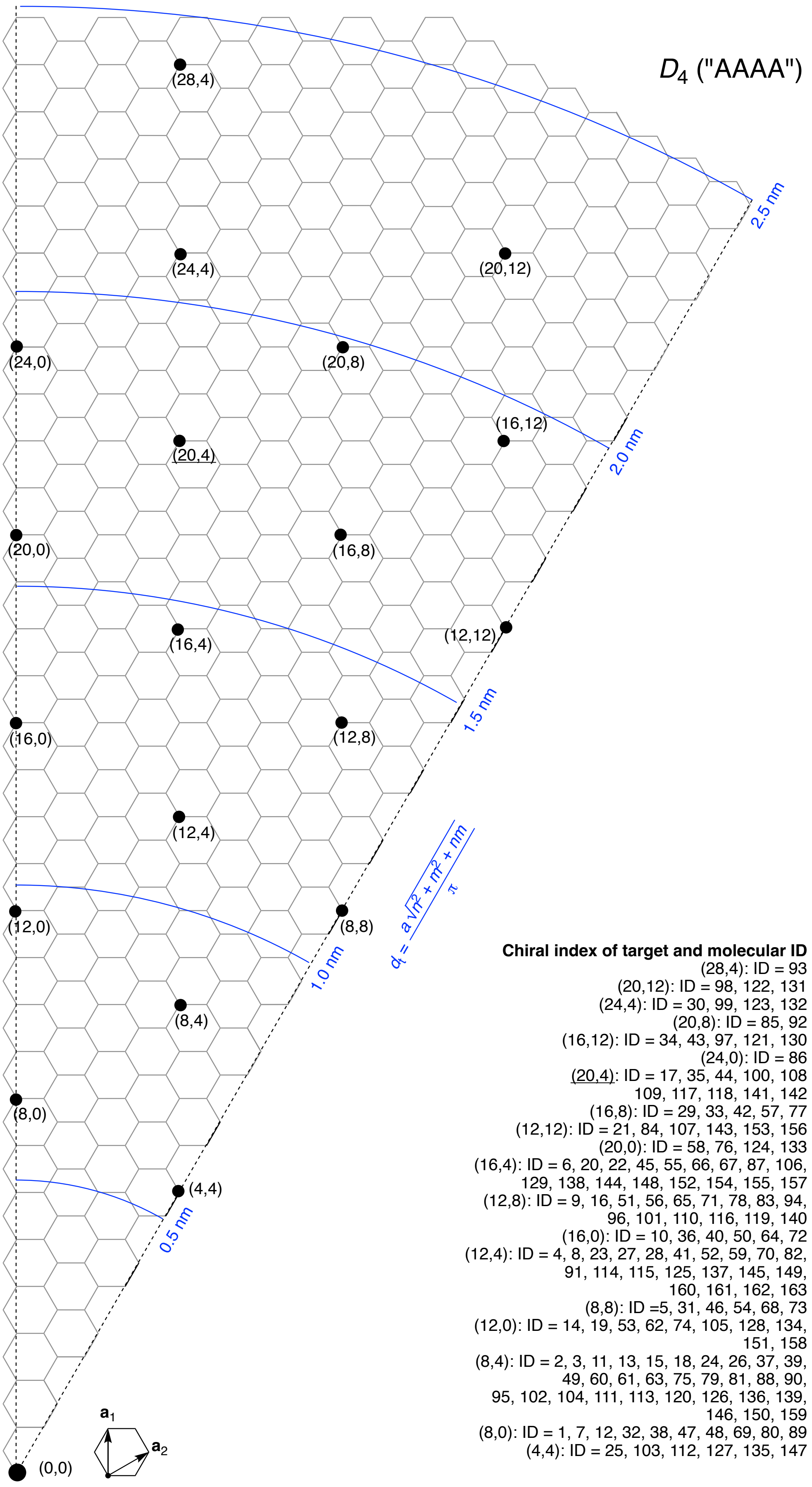

Supplementary Fig. 2. Synthetically accessible nanotube molecules with D_4 symmetry

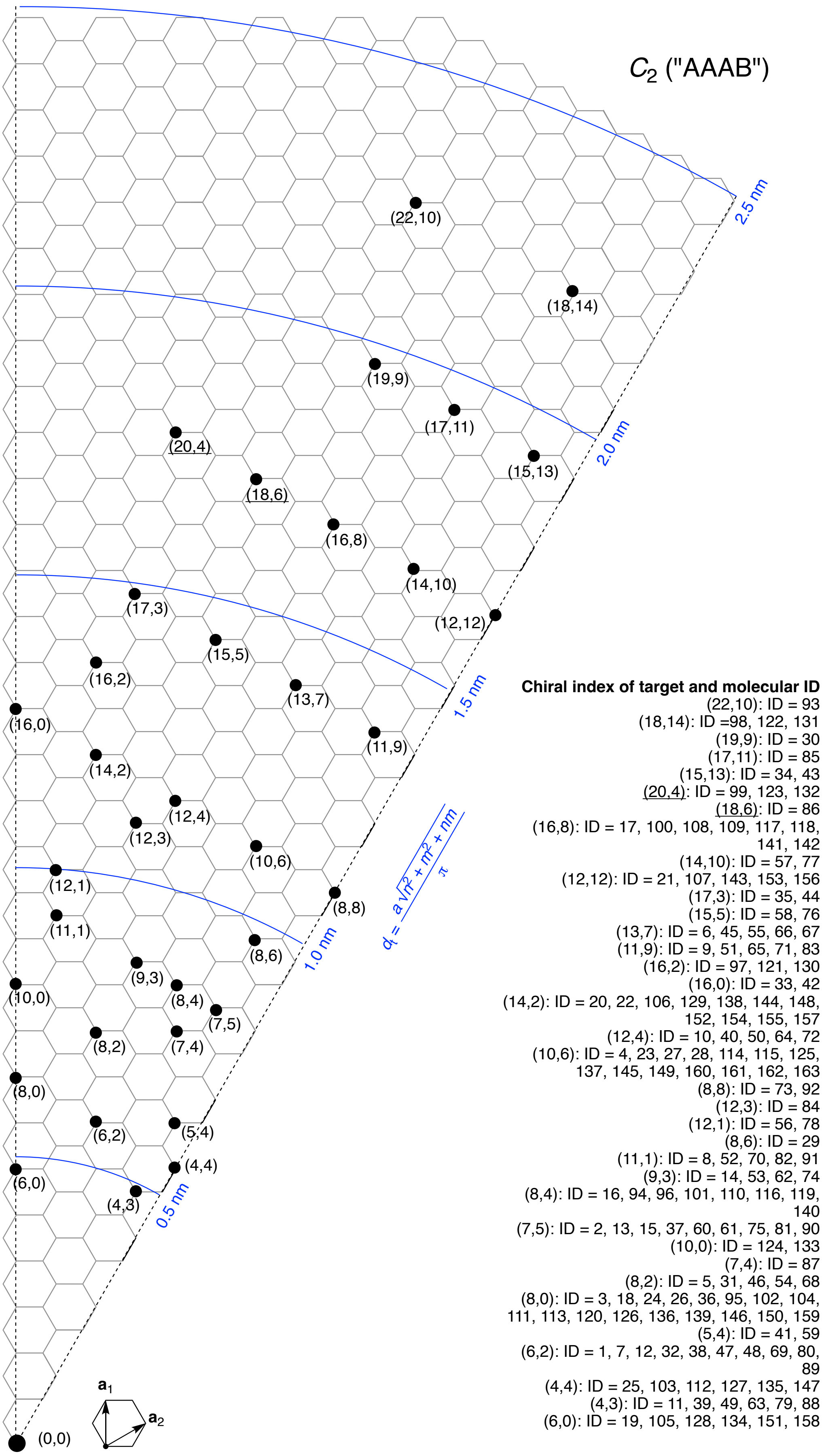

$d_t = \frac{a\sqrt{n^2 + m^2 + nm}}{\pi}$

Chiral index of target and molecular ID

(22,10): ID = 93
(18,14): ID =98, 122, 131
(19,9): ID = 30
(17,11): ID = 85
(15,13): ID = 34, 43
(20,4): ID = 99, 123, 132
(18,6): ID = 86
(16,8): ID = 17, 100, 108, 109, 117, 118, 141, 142
(14,10): ID = 57, 77
(12,12): ID = 21, 107, 143, 153, 156
(17,3): ID = 35, 44
(15,5): ID = 58, 76
(13,7): ID = 6, 45, 55, 66, 67
(11,9): ID = 9, 51, 65, 71, 83
(16,2): ID = 97, 121, 130
(16,0): ID = 33, 42
(14,2): ID = 20, 22, 106, 129, 138, 144, 148, 152, 154, 155, 157
(12,4): ID = 10, 40, 50, 64, 72
(10,6): ID = 4, 23, 27, 28, 114, 115, 125, 137, 145, 149, 160, 161, 162, 163
(8,8): ID = 73, 92
(12,3): ID = 84
(12,1): ID = 56, 78
(8,6): ID = 29
(11,1): ID = 8, 52, 70, 82, 91
(9,3): ID = 14, 53, 62, 74
(8,4): ID = 16, 94, 96, 101, 110, 116, 119, 140
(7,5): ID = 2, 13, 15, 37, 60, 61, 75, 81, 90
(10,0): ID = 124, 133
(7,4): ID = 87
(8,2): ID = 5, 31, 46, 54, 68
(8,0): ID = 3, 18, 24, 26, 36, 95, 102, 104, 111, 113, 120, 126, 136, 139, 146, 150, 159
(5,4): ID = 41, 59
(6,2): ID = 1, 7, 12, 32, 38, 47, 48, 69, 80, 89
(4,4): ID = 25, 103, 112, 127, 135, 147
(4,3): ID = 11, 39, 49, 63, 79, 88
(6,0): ID = 19, 105, 128, 134, 151, 158

Supplementary Fig. 3. Synthetically accessible nanotube molecules with C_2 symmetry

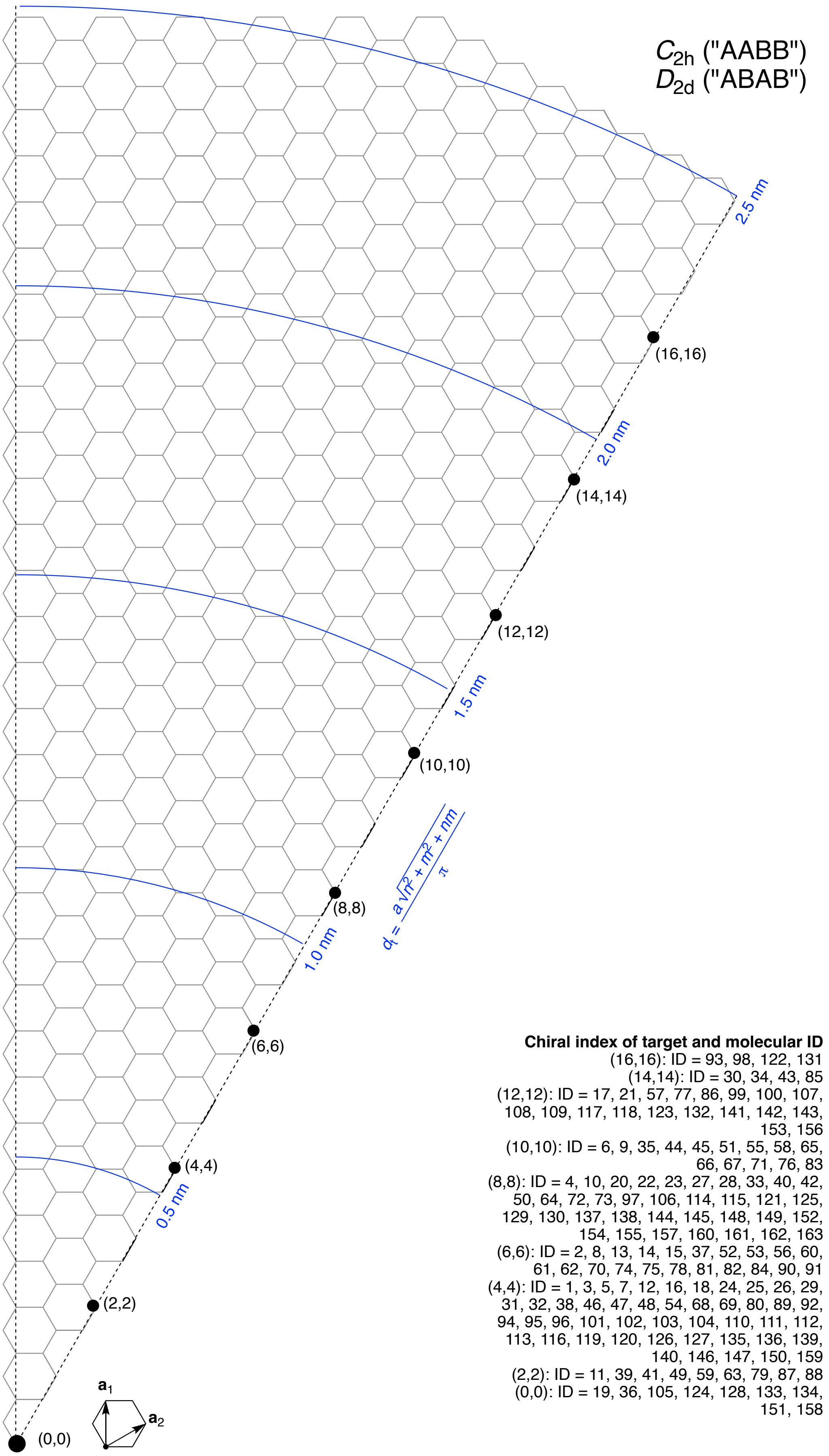

Supplementary Fig. 4. Synthetically accessible nanotube molecules with C_{2h} and D_{2d} symmetry

Synthesis

N,*N*-Dihexylquinacridone (**4**)

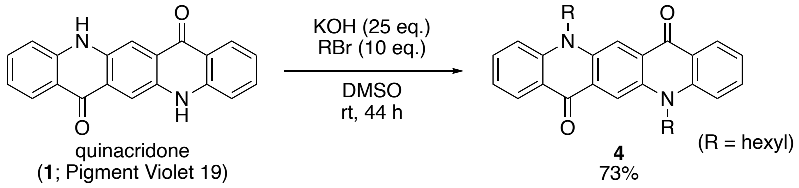

A mixture of quinacridone (**1**) (50.0 g, 160 mmol), DMSO (500 mL) and KOH (225 g, 4.00 mol) was stirred in a 2-L three-necked round bottom flask under atmosphere of air at ambient temperature for 30 min. To the dark blue suspension was added 1-bromohexane (264 g, 1.60 mmol), and the mixture was stirred for 44 h at ambient temperature. After the addition of water (1.0 L), the precipitate was collected by filtration. The precipitate was washed with water (1.0 L), methanol (800 mL), CH_2Cl_2 (200 mL) and $CHCl_3$ (300 mL) to afford **4** as an orange solid (37.5 g, 77.9 mmol). The alkylated compound **4** remained in the solution-phase filtrate was purified by using silica gel column chromatography (eluent: 1% EtOAc/$CHCl_3$) to afford **4** as an orange solid (18.8 g, 39.1 mmol). In total, the compound **4** was obtained in 73% yield (56.3 g, 117 mmol). Spectral data were identical to those reported in the literature.[4]

2,9-Dibromo-*N*,*N*-dihexylquinacridone (**2**)

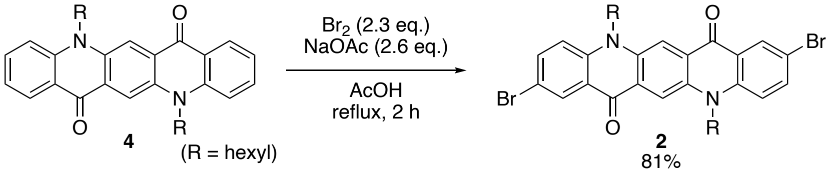

In a 5-L three-necked round-bottom flask were added **4** (50.0 g, 104 mmol), NaOAc (22.2 g, 271 mmol) and AcOH (1.25 L), and the mixture was refluxed. To the mixture was added bromine (38.2 g, 12.3 mL, 239 mmol) in acetic acid (1.0 L) in a dropwise manner. After the addition, the mixture was refluxed for 2 h. The precipitate was collected by filtration and washed with aq. $NaHSO_3$ (800 mL), water (1.5 L) and methanol (800 mL). The resulting solid was purified by recrystallization from toluene to afford **2** as a red solid in 81% yield (53.6 g, 84.0 mmol). Spectral data were identical to those reported in the literature.[4]

2,9-Bis(4,4,5,5-tetramethyl-1,3,2-dioxaborolan-2-yl)-*N*,*N*-dihexylquinacridone (**3**)

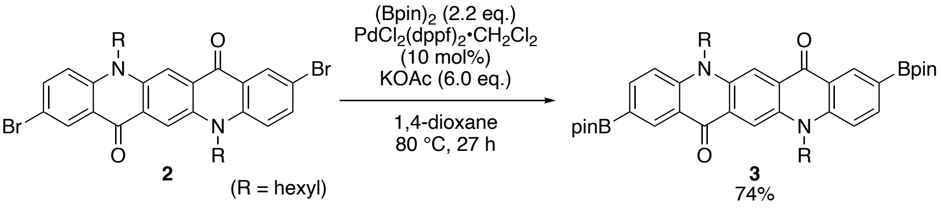

In a 1-L three-necked round-bottom flask were added KOAc (46.1 g, 470 mmol), $(Bpin)_2$ (43.8 g, 172 mmol), $PdCl_2(dppf)•CH_2Cl_2$ (6.40 g, 7.83 mmol), **2** (50.0 g, 78.3 mmol) and 1,4-dioxane (400 mL). The mixture was stirred at 80 °C for 27 h and was poured into water (1.0 L) after cooling to ambient temperature. To the mixuture was added $CHCl_3$ (1.5 L), and the organic layer was collected. The aqueous layer was further extracted with $CHCl_3$ (100 mL × 4). The combined organic layer was washed with brine, dried over Na_2SO_4 and concentrated in vacuo. The crude material was passed through a short pad of silica gel (eluent: 15% EtOAc/$CHCl_3$) and purified by recrystallization from toluene to afford **3** as an orange solid in 74% yield (42.5 g, 58.0 mmol). ^{1}H NMR (600 MHz, $CDCl_3$): δ 9.06 (d, J = 1.6 Hz, 2H), 8.80 (s, 2H), 8.14 (dd, J = 8.8, 1.6 Hz, 2H), 7.50 (d, J = 8.8 Hz, 2H), 4.54 (t, J = 7.8 Hz, 4H), 2.00 (m, 4H), 1.63 (m, 4H), 1.49-1.38 (m, 32H), 0.95 (t, J = 7.3 Hz, 6H); ^{13}C NMR (151 MHz, $CDCl_3$): δ 178.3, 157.0, 144.2, 140.2 (CH), 136.3 (CH), 136.0, 126.9, 120.8, 114.0 (CH), 113.9 (CH), 84.1, 46.4 (CH_2), 31.7 (CH_2), 27.2 (CH_2), 26.8 (CH_2), 25.1 (CH_3), 22.8 (CH_2), 14.2 (CH_3); HRMS (MALDI-TOF) (m/z): $[M]^+$ calcd. for $C_{44}H_{58}B_2N_2O_6$ 732.4490, found 732.4485.

*Crystal structure of rac-**[4]CQ***

A single crystal of *rac*-**[4]CQ** was obtained from a toluene solution in the presence of atmospheric vapor of methanol at 25 °C. The crystal was mounted on a thin polymer tip with cryoprotectant oil and was frozen via flash cooling. The diffraction analyses with synchrotron X-ray source were carried out at 100 K at the BL26B1 beamline in SPring-8 using a diffractometer equipped with a Dectris EIGER X 4M PAD detector. The diffraction data were processed with the XDS software program.[5] The structures were solved by a direct method with the SHELXT software program[6] and refined by full-matrix least-squares on F^2 using the SHELXL-2018/3 program suite[7] running with the Yadokari-XG 2009 software program.[8] In the refinements, alkyl groups were restrained by SIMU, DFIX and DANG. The non-hydrogen atoms were analyzed anisotropically and hydrogen atoms were input at the calculated positions and refined with a riding model. The electron density attributed to solvent molecules could not be modeled due to the severe disorders, and the structures were treated by using the PLATON/SQUEEZE protocol.[9,10] Crystal data and structure refinement are listed in Supplementary Table 1. We found two independent [4]CQ molecules with minor structural differences. A representative molecule was shown in Fig. 3b, and both molecules are shown in Supplementary Fig. 5. Because of an empirical formula of $C_{128}H_{136}N_8O_8$ with a high molecular weight as a small molecule, a structural biology beamline (BL26B1) at SPring-8 was used.[11] Although several crystals were examined, the high-angle data could not be located partly due to disordered structures. The crystal contained many disordered solvent molecules that were removed by SQUEEZE and disordered structures at alkyl chains repelled symmetrizations of two enantiomeric structures. Among several crystals examined for the diffraction analyses, the present data were the

best in the quality to collect 82643 reflections with 25085 independent reflections but were not fully sufficient to cover the two independent molecules of $C_{128}H_{136}N_8O_8$. Despite the presence of Alert A and Alert B from CheckCIF, structural details were chemically reasonable.

Supplementary Table 1. Crystal data and structure refinement for *rac*-**[4]CQ**

CCDC	2204308
Empirical formula	C_{128} H_{136} N_8 O_8
Formula weight	1914.44
Temperature	100(2) K
Wavelength	0.8000 Å
Crystal system	Monoclinic
Space group	*Cc*
Unit cell dimensions	a = 36.170(7) Å α = 90°.
	b = 35.220(7) Å β = 107.63(3)°.
	c = 22.980(5) Å γ = 90°.
Volume	27899(11) $Å^3$
Z	8
Density (calculated)	0.912 Mg/m^3
Absorption coefficient	0.072 mm^{-1}
F(000)	8192
Crystal size	0.100 × 0.100 × 0.050 mm^3
Theta range for data collection	0.930 to 22.391°.
Index ranges	$-34<=h<=34$, $-33<=k<=33$, $-21<=l<=21$
Reflections collected	82643
Independent reflections	25085 [R(int) = 0.1172]
Completeness to theta = 22.391°	99.8 %
Absorption correction	Semi-empirical from equivalents
Max. and min. transmission	1.000 and 0.492
Refinement method	Full-matrix least-squares on F^2
Data / restraints / parameters	25085 / 2113 / 2658
Goodness-of-fit on F^2	0.973
Final *R* indices [I>2sigma(I)]	R_1 = 0.1085, wR_2 = 0.2721
R indices (all data)	R_1 = 0.2096, wR_2 = 0.3516
Absolute structure parameter	–0.5(10)
Extinction coefficient	n/a

Largest diff. peak and hole	0.424 and –0.296 e.Å^{-3}

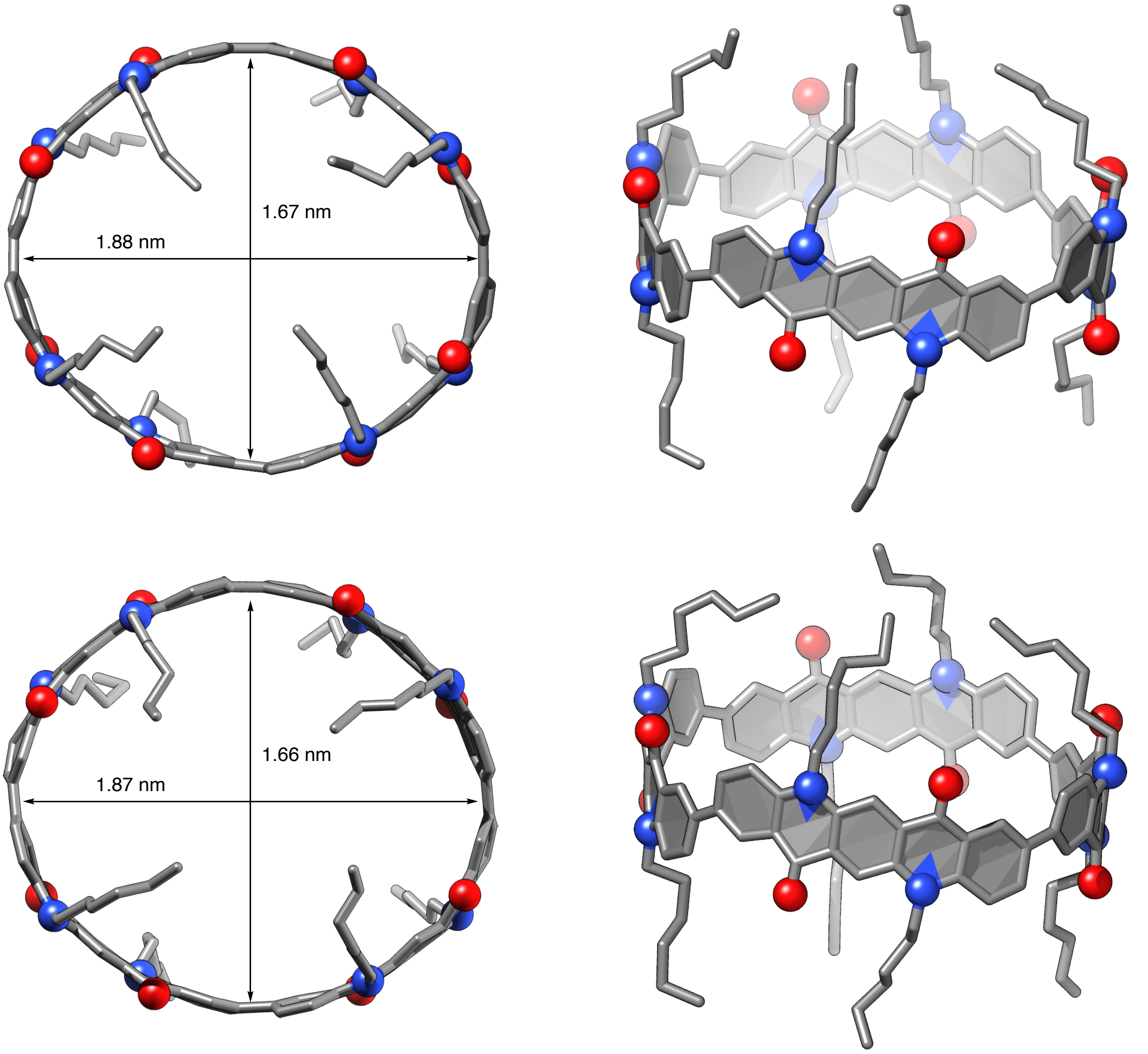

Supplementary Fig. 5. Crystal structures of *rac*-**[4]CQ**, showing two independent molecules found in the crystal.

Chiral resolution of ***[4]CQ***

The presence of enantiomers with **[4]CQ** was confirmed by analytical HPLC using a COSMOSIL Cholester column (4.6ϕ × 250 mm, Nacalai Tesque) (eluent = 50% methanol/dichloromethane, temperature = 40 °C and flow rate = 1.0 mL/min) (Fig. 4a). Two enantiomers were separated in a preparative scale by using the COSMOSIL Cholester column of 20ϕ × 250 mm (eluent: 50% methanol/dichloromethane, temperature = ambient temperature, flow rate = 18 mL/min) (Supplementary Fig. 6). By using *rac*-**[4]CQ** (6.1 mg), an enantiomer, $(+)_{275}$-**[4]CQ**, was first obtained in a pure form as the first fraction (**fr. 1-1**; 2.5 mg) of the first run from 13 min. The second fraction ($(-)_{275}$-**[4]CQ**, **fr. 2-1**; 3.6 mg) of the first run was not completely pure and contained ~1% of $(+)_{275}$-isomer. A second run of HPLC was performed with **fr. 2-1** to afford enantiopure $(-)_{275}$-

[4]CQ (**fr. 2-2**; 2.1 mg).

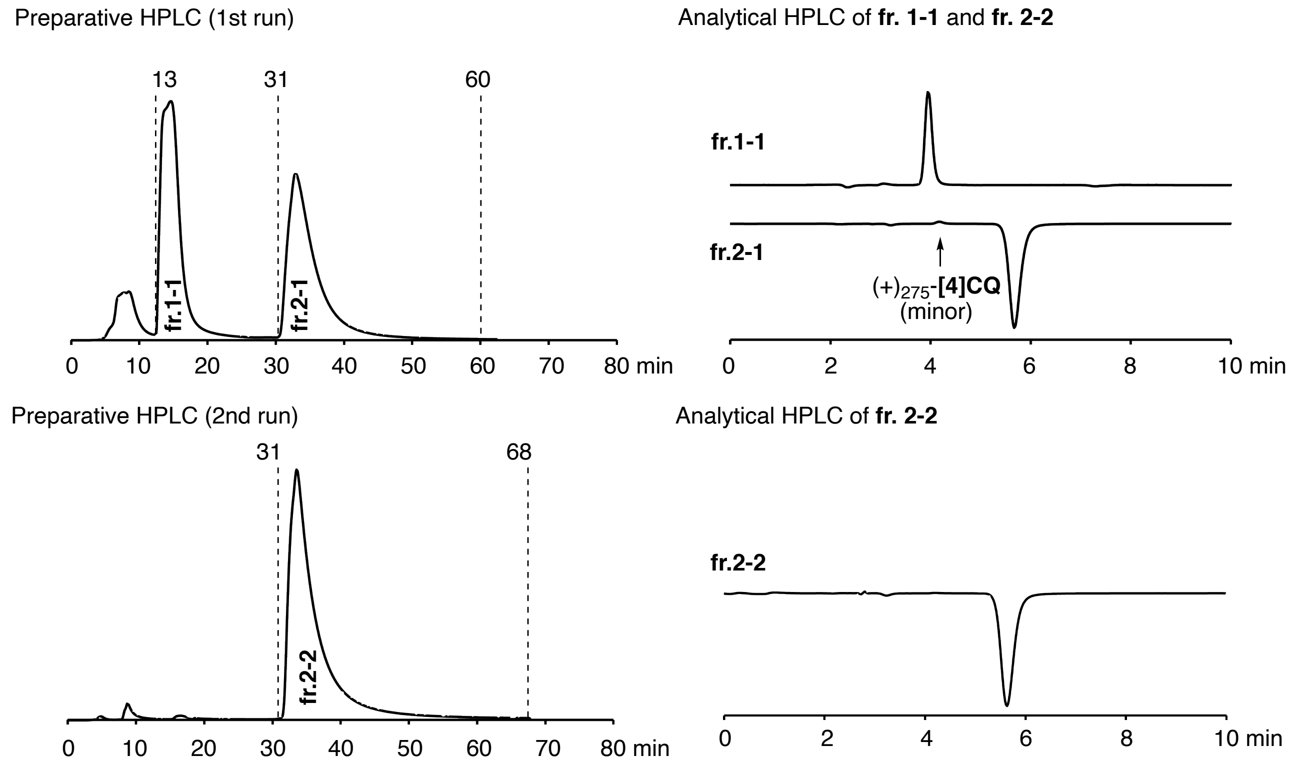

Supplementary Fig. 6. Chiral resolution of *rac*-**[4]CQ**

Theoretical calculations

The Gaussian 16 program suite[12] was used for DFT calculations of (*P*)-**[4]CQ** with methyl-substituted models at the B3LYP/6-31G(d,p) level of theory (Supplementary Table 3).[13,14,15,16,17,18,19,20] Spectra calculations were performed by TD DFT for the first 200 singlet–singlet transitions with an extra keyword of IOP(9/40 = 8) to output information on smaller contributions to each electronic transition. The theoretical CD spectrum was simulated with the peak half-width at half height of 0.15 eV by using GaussView 6.0.16. The theoretical CD spectrum of (*P*)-**[4]CQ** matched with $(+)_{275}$-**[4]CQ** as shown in Fig. 4b, which allowed us to assign $(+)_{275}$-**[4]CQ** as (*P*) for the helicity.

NMR spectra of i-DWNT: In-and-out exchange

In-and-out exchange processes of i-DWNT assembly were examined by varying the mixing ratio of **[4]CQ** and **[3]C^{db}C**. We first describe procedures for the heterohelical combinations. A specimen of (*P*)-**[4]CQ** (2.4052 mg, 1.2563 μmol) was dissolved in CD_2Cl_2 (1.26 mL), and a specimen of (*M*)-**[3]C^{db}C** (1.8989 mg, 1.2794 μmol) was dissolved in CD_2Cl_2 (1.28 mL). The solution of (*P*)-**[4]CQ**

(0.40 mL) and the solution of (*M*)-**[3]C^{db}C** (0.20 mL) were mixed to give a 2:1 mixture of (*P*)-**[4]CQ** and (*M*)-**[3]C^{db}C** to record the spectrum shown in Supplementary Fig. 7 (top). The solution of (*P*)-**[4]CQ** (0.20 mL) and the solution of (*M*)-**[3]C^{db}C** (0.40 mL) were then mixed to give a 1:2 mixture of (*P*)-**[4]CQ** and (*M*)-**[3]C^{db}C** to record the spectrum shown in Supplementary Fig. 7 (bottom).

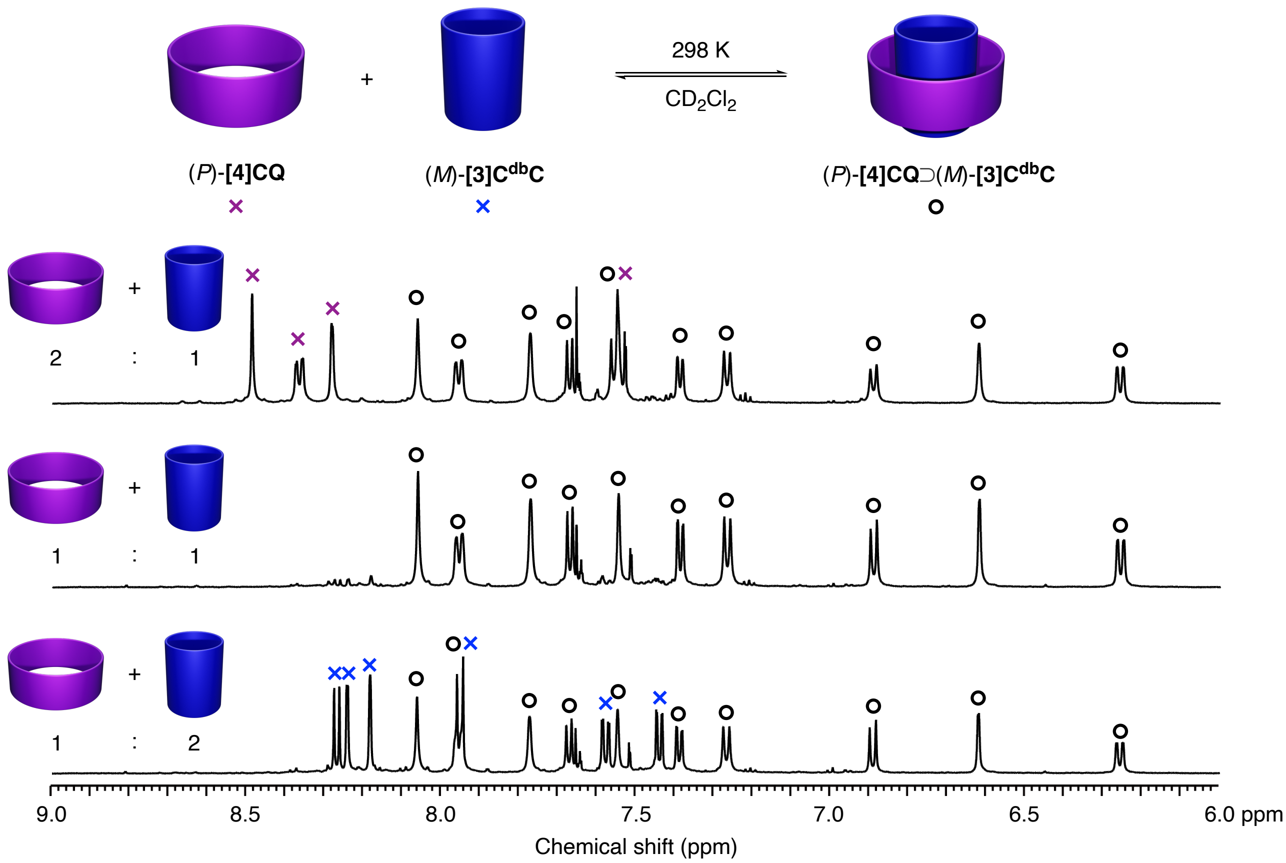

Supplementary Fig. 7. NMR spectra of the mixture of (*P*)-**[4]CQ** and (*M*)-**[3]C^{db}C** with different ratio (CD_2Cl_2, 600 MHz, 298 K)

Procedures for the homohelical combinations are as follow. A specimen of (*M*)-**[4]CQ** (1.8618 mg, 0.9725 μmol) was dissolved in CD_2Cl_2 (0.97 mL), and a specimen of (*M*)-**[3]C^{db}C** (1.4671 mg, 0.9885 μmol) was dissolved in CD_2Cl_2 (0.99 mL). The solution of (*M*)-**[4]CQ** (0.40 mL) and the solution of (*M*)-**[3]C^{db}C** (0.20 mL) were mixed to give a 2:1 mixture of (*M*)-**[4]CQ** and (*M*)-**[3]C^{db}C** to record the spectrum shown in Supplementary Fig. 8 (top). The solution of (*M*)-**[4]CQ** (0.20 mL) and the solution of (*M*)-**[3]C^{db}C** (0.40 mL) were then mixed to give a 1:2 mixture of (*M*)-**[4]CQ** and (*M*)-**[3]C^{db}C** to record the spectrum shown in Supplementary Fig. 8 (bottom).

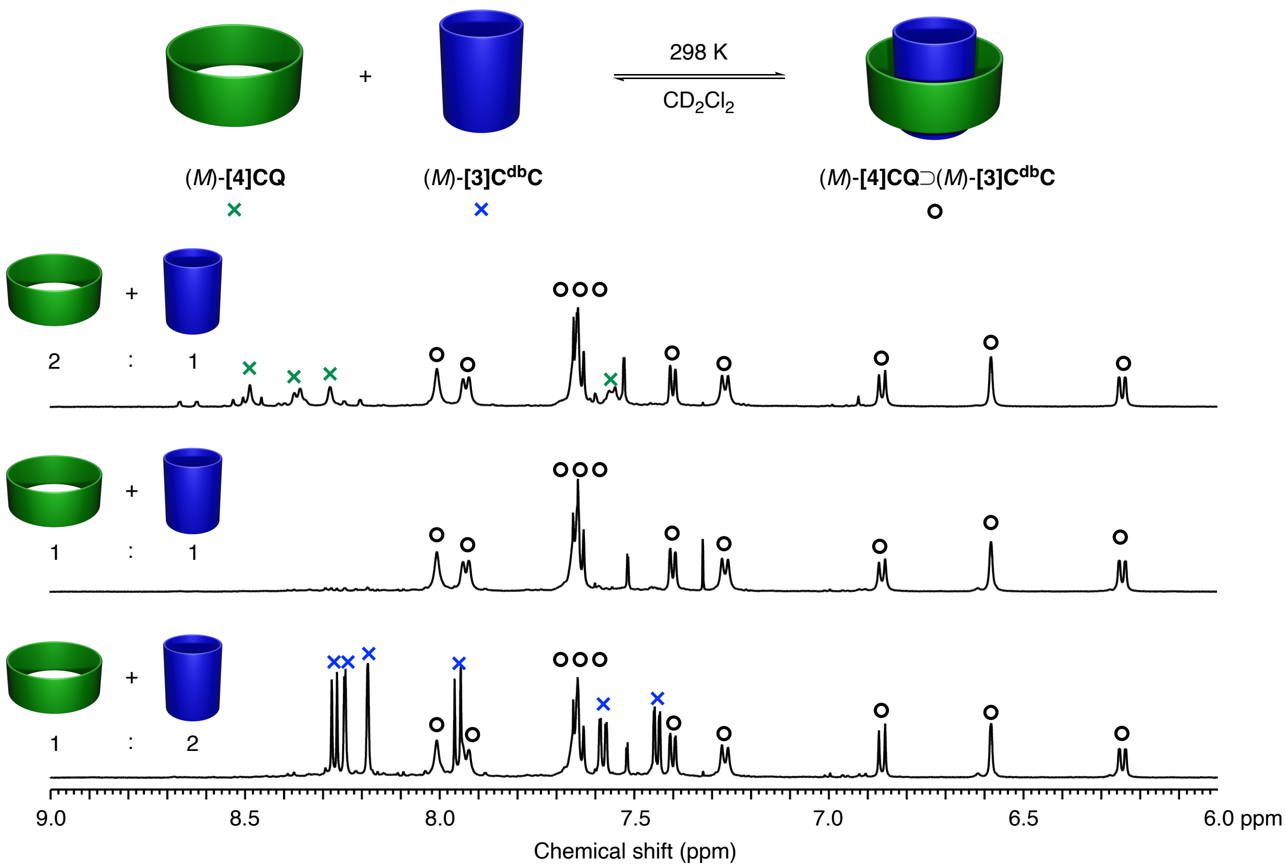

Supplementary Fig. 8. NMR spectra of the mixture of (*M*)-**[4]CQ** and (*M*)-**[3]C^{db}C** with different ratio (CD_2Cl_2, 600 MHz, 298 K)

As shown in Supplementary Figs. 7 and 8, when the stoichiometry of the inner and outer tubes deviated from an equimolar amount, an uncomplexed form of the tube was observed separately from a complexed form. The observations showed that the uncomplexed species and the complexed species existed in solution as independent species. Therefore, the in-and-out exchange processes present for the equilibrium of i-DWNT assembly were slower than the NMR time scale. In addition, the ^{1}H NMR spectra of i-DWNT complexes showed the presence of axial symmetry operations to simplify the spectra, which revealed that the rotational motions were faster than the NMR time scale (Supplementary Fig. 9).

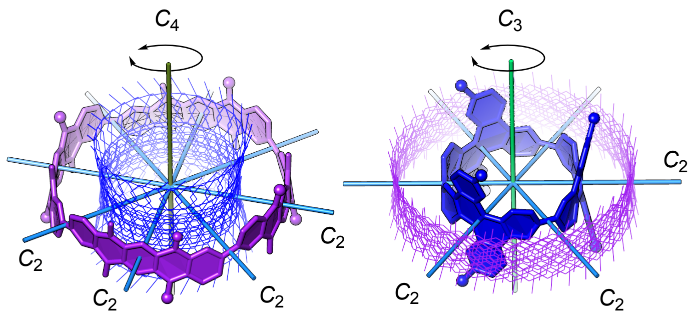

Supplementary Fig. 9. Axial rotational motions necessary to simplify the ^{1}H NMR spectra

*Crystal structure of (P)-**[4]CQ⊃**(M)-**[3]C^db^C***

A single crystal of (*P*)-**[4]CQ⊃**(*M*)-**[3]C^{db}C** was obtained from a solution in 50% methanol/dichloromethane at 5 °C. The crystal was mounted on a thin polymer tip with cryoprotectant oil and was frozen via flash cooling. The diffraction analyses with synchrotron X-ray source were carried out at 95 K at the BL17A beamline of KEK Photon Factory using a diffractometer equipped with a Dectris EIGER X 16M PAD detector. The diffraction data were processed with the XDS software program.[5] The structures were solved by a direct method with the SHELXT software program[6] and refined by full-matrix least-squares on F^2 using the SHELXL-2018/3 program suite[7] running with the Yadokari-XG 2009 software program.[8] In the refinements, alkyl groups and solvent molecules were restrained by SIMU, DELU, ISOR, DFIX and DANG. The non-hydrogen atoms were analyzed anisotropically and hydrogen atoms were input at the calculated positions and refined with a riding model. Crystal data and structure refinement are listed in Supplementary Table 2. Because of an empirical formula of $C_{244.38}H_{254.76}Cl_{4.76}N_8O_8$ with a high molecular weight as a small molecule, a beamline (BL17A) at KEK was used. The quality of diffraction data did not completely meet high requirements because of the disordered structures including 8 conformational variants of alkyl chains and 38 dichloromethane molecules. Among several crystals examined for the diffraction analyses, the present data were the best in the quality to collect 1102372 reflections with 43298 independent reflections but were not fully sufficient to cover the two independent molecules of $C_{244.38}H_{254.76}Cl_{4.76}N_8O_8$. Despite the presence of Alert B from CheckCIF, structural details were chemically reasonable.

Supplementary Table 2. Crystal data and structure refinement for (*P*)-**[4]CQ⊃**(*M*)-**[3]C^{db}C**

CCDC	2204309	
Empirical formula	$C_{244.38}$ $H_{254.76}$ $Cl_{4.76}$ N_8 O_8	
Formula weight	3600.69	
Temperature	95(2) K	
Wavelength	0.9000 Å	
Crystal system	Tetragonal	
Space group	$P4_32_12$	
Unit cell dimensions	a = 26.420(4) Å	α = 90°.
	b = 26.420(4) Å	β = 90°.
	c = 68.460(14) Å	γ = 90°.
Volume	47786(16) Å^3	
Z	8	

Density (calculated)	1.001 Mg/m^3
Absorption coefficient	0.200 mm^{-1}
F(000)	15376
Crystal size	0.300 × 0.050 × 0.040 mm^3
Theta range for data collection	1.046 to 32.819°.
Limiting indices	–31<=*h*<=31, –31<=*k*<=31, –79<=*l*<=79
Reflections collected	1102372
Independent reflections	43298 [*R*(int) = 0.0442]
Completeness to theta = 32.684°	99.0 %
Absorption correction	Semi-empirical from equivalents
Max. and min. transmission	1.000 and 0.691
Refinement method	Full-matrix least-squares on F^2
Data / restraints / parameters	43298 / 2138 / 3494
Goodness-of-fit on F^2	1.186
Final *R* indices [*I*>2sigma(*I*)]	R_1 = 0.1367, wR_2 = 0.2923
R indices (all data)	R_1 = 0.1645, wR_2 = 0.3357
Absolute structure parameter	0.451(15)
Extinction coefficient	n/a
Largest diff. peak and hole	0.619 and -0.385 e.Å^{-3}

Hirshfeld surface analyses

The Hirshfeld surface analyses were performed by using CrystalExplorer (v. 17.5)[21] with the present data of (*P*)-**[4]CQ**⊃(*M*)-**[3]C^{db}C** and reported data of (*P*)-**[4]CF**⊃(*M*)-**[3]C^{db}C**.[22] The surfaces on sp^2-hybridized atoms (C, N, O) were created at a high resolution, and surface areas of π-contacts were determined by using d_i *vs* d_e mode. The inner tube contained solvent molecules of dichloromethane but had contacts solely with H and Cl. The surface area analyses thus allowed us to derive information on the π-contacts at the interface of inner and outer tubes. For visual inspections, the Hirshfeld surfaces for each panel were separately created and were used for figures.

a
(*P*)-**[4]CQ**⊃(*M*)-**[3]C**^{db}**C**

b
(*P*)-**[4]CF**⊃(*M*)-**[3]C**^{db}**C**

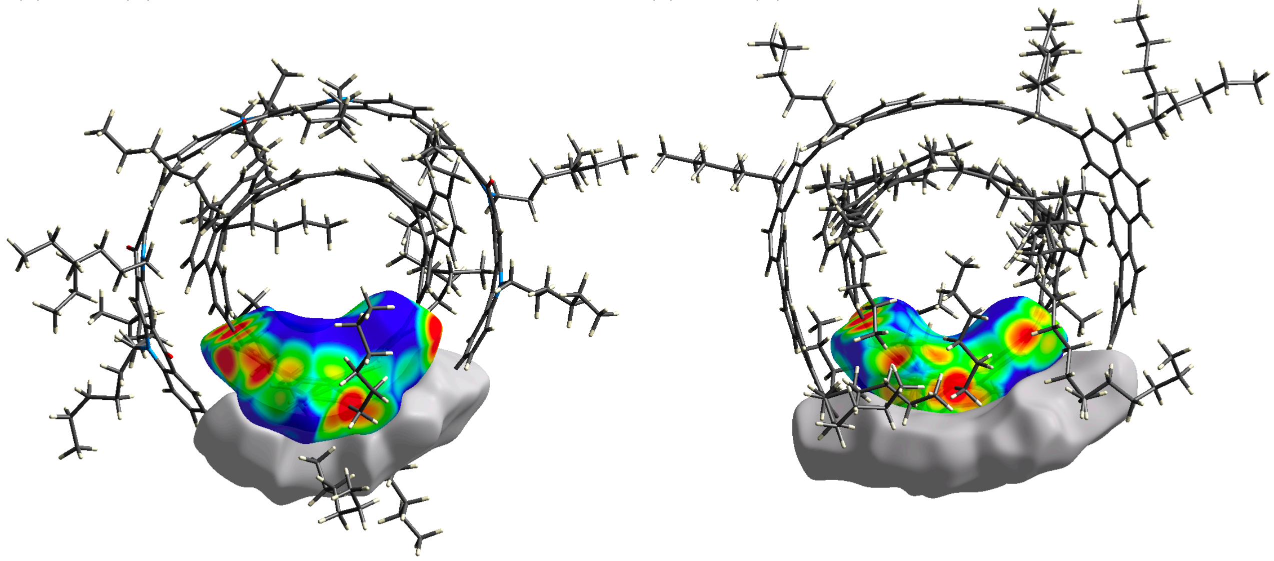

Supplementary Fig. 10. Crystal structures of i-DWNT complexes showing panel-panel overlaps. **a** (*P*)-**[4]CQ**⊃(*M*)-**[3]C**^{db}**C**. **b** (*P*)-**[4]CF**⊃(*M*)-**[3]C**^{db}**C**. See also Fig. 7.

Supplementary References

1. Onaka, Y., Tanaka, S., Kobayashi, A., Matsuno, T. & Isobe, H. A large-bore chiral cylindrical molecule prone to radial deformations. *Tetrahedron Lett.* **96**, 153774 (2022).
2. Saito, R., Dresselhaus, G. & Dresselhaus, M. S. *Physical Properties of carbon nanotubes* (Imperial College Press, 1998).
3. Komatsu, N. Stereochemistry of carbon nanotubes. *Jpn. J. Appl. Phys.* **49**, 02BC01 (2010).
4. Song, H. J., Lee, S. M., Lee, J. Y., Choi, B. H. & Moon, D. K. The synthesis and electroluminescent properties of dithienylquinacridone-based copolymers for white light-emitting diodes. *Synth. Met.* **161**, 2451-2459 (2011).
5. Kabsch, W. Automatic processing of rotation diffraction data from crystals of initially unknown symmetry and cell constants. *J. Appl. Crystallogr.* **26**, 795-800 (1993).
6. Sheldrick, G. M. *SHELXT* - Integrated space-group and crystal-structure determination. *Acta Crystallogr.* **A71**, 3-8 (2015).
7. Sheldrick, G. M. & Schneider, T. R. in *Macromolec. Crystal. B Book Series: Methods in Enzymology, Vol. 277*, 319-343 (Academic Press, 1997).
8. Kabuto, C., Akine, S., Nemoto, T. & Kwon, E. Release of software (Yadokari-XG 2009) for crystal structure analyses. *J. Crystallogr. Soc. Jpn.* **51**, 218-224 (2009).
9. Spek, A. L. Single-crystal structure validation with the program *PLATON*. *J. Appl. Crystallogr.* **36**, 7-13 (2003).

10. van der Sluis, P. & Spek, A. L. BYPASS: an effective method for the refinement of crystal structures containing disordered solvent regions. *Acta Crystallogr.* **A46**, 194-201 (1990).

11. Ueno, G., Kanda, H., Hirose, R., Ida, K., Kumasaka, T. & Yamamoto, M. RIKEN structural genomics beamlines at the SPring-8; high throughput protein crystallography with automated beamline operation. *J. Struct. Funct. Genomics* **7**, 15-22 (2006).

12. Frisch, M. J.; Trucks, G. W.; Schlegel, H. B.; Scuseria, G. E.; Robb, M. A.; Cheeseman, J. R.; Scalmani, G.; Barone, V.; Petersson, G. A.; Nakatsuji, H.; Li, X.; Caricato, M.; Marenich, A. V.; Bloino, J.; Janesko, B. G.; Gomperts, R.; Mennucci, B.; Hratchian, H. P.; Ortiz, J. V.; Izmaylov, A. F.; Sonnenberg, J. L.; Williams-Young, D.; Ding, F.; Lipparini, F.; Egidi, F.; Goings, J.; Peng, B.; Petrone, A.; Henderson, T.; Ranasinghe, D.; Zakrzewski, V. G.; Gao, J.; Rega, N.; Zheng, G.; Liang, W.; Hada, M.; Ehara, M.; Toyota, K.; Fukuda, R.; Hasegawa, J.; Ishida, M.; Nakajima, T.; Honda, Y.; Kitao, O.; Nakai, H.; Vreven, T.; Throssell, K.; Montgomery, J. A., Jr.; Peralta, J. E.; Ogliaro, F.; Bearpark, M. J.; Heyd, J. J.; Brothers, E. N.; Kudin, K. N.; Staroverov, V. N.; Keith, T. A.; Kobayashi, R.; Normand, J.; Raghavachari, K.; Rendell, A. P.; Burant, J. C.; Iyengar, S. S.; Tomasi, J.; Cossi, M.; Millam, J. M.; Klene, M.; Adamo, C.; Cammi, R.; Ochterski, J. W.; Martin, R. L.; Morokuma, K.; Farkas, O.; Foresman, J. B.; Fox, D. J. *Gaussian 16, Revision C.01*, (Gaussian, Inc., Wallingford CT, 2016).

13. Becke, A. D. Density-functional exchange-energy approximation with correct asymptotic behavior. *Phys. Rev. A* **38**, 3098-3100 (1988).

14. Becke, A. D. Density-functional thermochemistry. III. The role of exact exchange. *J. Chem. Phys.* **98**, 5648-5652 (1993).

15. Lee, C., Yang, W. & Parr, R. G. Development of the Colle-Salvetti correlation-energy formula into a functional of the electron density. *Phys. Rev. B* **37**, 785-789 (1988).

16. Ditchfield, R., Hehre, W. J. & Pople, J. A. Self-consistent molecular-orbital methods. IX. An extended Gaussian-type basis for molecular-orbital studies of organic molecules. *J. Chem. Phys.* **54**, 724-728 (1971).

17. Hehre, W. J., Ditchfield, R. & Pople, J. A. Self-consistent molecular orbital methods. XII. Further extentions of Gaussian-type basis sets for use in molecular orbital studies of organic molecules. *J. Chem. Phys.* **56**, 2257-2261 (1972).

18. Hariharan, P. C. & Pople, J. A. Accuracy of AH_n equilibrium geometries by single determinant molecular orbital theory. *Mol. Phys.* **27**, 209-214 (1974).

19. Gordon, M. S. The isomers of silacyclopropane. *Chem. Phys. Lett.* **76**, 163-168 (1980).

20. Hariharan, P. C. & Pople, J. A. The influence of polarization functions on molecular orbital hydrogenation energies. *Theor. Chim. Acta.* **28**, 213-222 (1973).

21. Spackman, P. R., Turner, M. J., McKinnon, J. J., Wolff, S. K., Grimwood, D. J., Jayatilaka, D. &

Spackman, M. A. CrystalExplorer: a program for Hirshfeld surface analysis, visualization and quantitative analysis of molecular crystals. *J. Appl. Crystallogr.* **54**, 1006-1011 (2021).
22. Matsuno, T., Ohtomo, Y., Someya, M. & Isobe, H. Stereoselectivity in spontaneous assembly of rolled incommensurate carbon bilayers. *Nat. Commun.* **12**, 1575 (2021).
