# Supplementary material for: Target-oriented design of helical nanotube molecules for rolled incommensurate bilayers
Source: Commun Chem. 2022 Nov 19;5:152. doi: 10.1038/s42004-022-00777-2 (PMC9814558; doi:10.1038/s42004-022-00777-2)
Supplement: Supplementary file 2 — Description of Additional Supplementary Files [file 42004_2022_777_MOESM2_ESM.pdf]

Description of Additional Supplementary Files

File name: Supplementary Data 1

Description: Chromatograms

File name: Supplementary Data 2

Description: NMR spectra.

File name: Supplementary Data 3

Description: UV-vis and CD spectra.

File name: Supplementary Data 4

Description: Titration data.

File name: Supplementary Data 5

Description: Data from DFT calculations.

File name: Supplementary Data 6

Description: X-ray crystallographic data of CCDC2204308 in the cif format.

File name: Supplementary Data 7

Description: X-ray crystallographic data of CCDC2204309 in the cif format.
