# Supplementary material for: Target-oriented design of helical nanotube molecules for rolled incommensurate bilayers
Source: Commun Chem. 2022 Nov 19;5:152. doi: 10.1038/s42004-022-00777-2 (PMC9814558; doi:10.1038/s42004-022-00777-2)
Supplement: Supplementary file 3 — Supplementary Data 1 [file 42004_2022_777_MOESM3_ESM.pdf]

*Chromatogram of **[4]CQ***

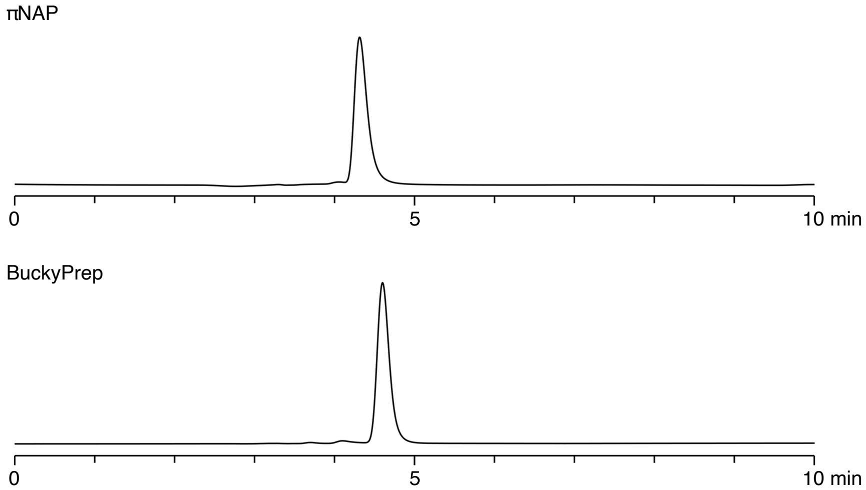

Chromatogram 1. HPLC chromatograms of *rac*-**[4]CQ**. Two different columns were used to confirm the purity (COSMOSIL πNAP, 4.6ϕ × 250 mm and COSMOSIL BuckyPrep, 4.6ϕ × 250 mm). Chromatographic conditions: flow rate = 1.0 mL min^{-1}, eluent = 40% MeOH/CH_2Cl_2 for COSMOSIL πNAP and 30% MeOH/CH_2Cl_2 for COSMOSIL BuckyPrep, UV detection = 300 nm.
