# Supplementary material for: Target-oriented design of helical nanotube molecules for rolled incommensurate bilayers
Source: Commun Chem. 2022 Nov 19;5:152. doi: 10.1038/s42004-022-00777-2 (PMC9814558; doi:10.1038/s42004-022-00777-2)
Supplement: Supplementary file 4 — Supplementary Data 2 [file 42004_2022_777_MOESM4_ESM.pdf]

NMR spectra

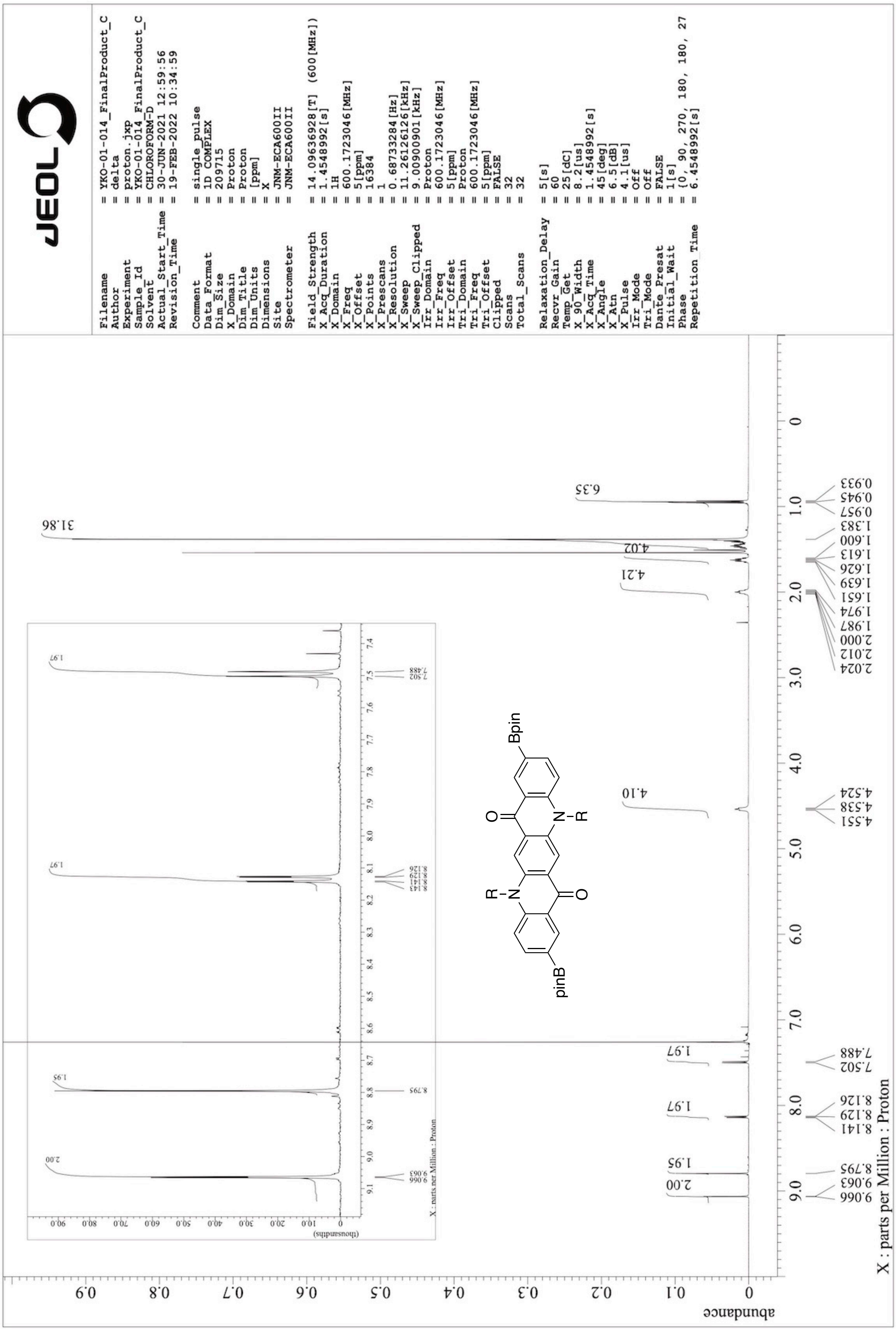

NMR spectrum 1. ^{1}H NMR spectrum of **3** ($CDCl_3$, 600 MHz, 298 K)

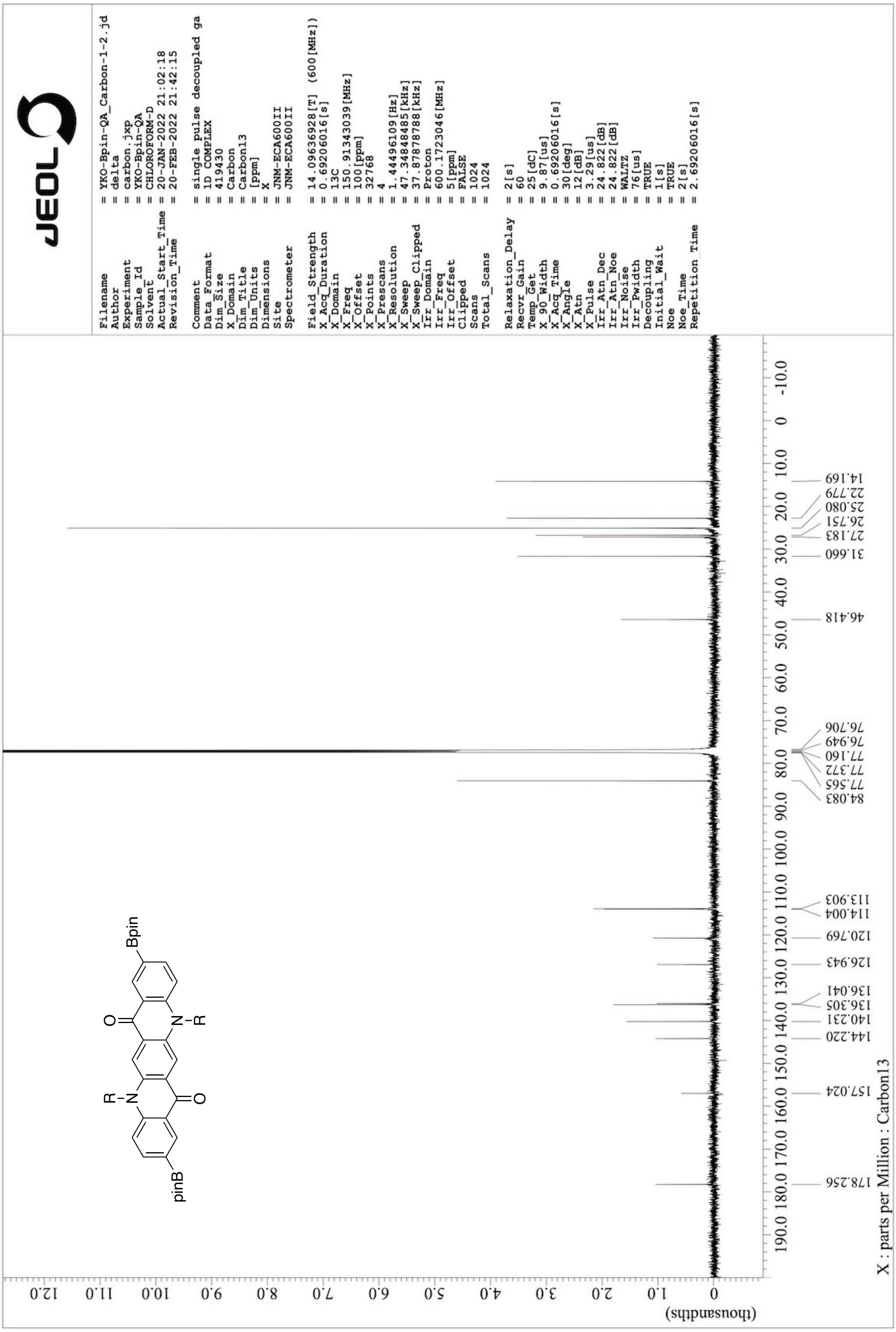

NMR spectrum 2. ^{13}C NMR spectrum of **3** ($CDCl_3$, 151 MHz, 298 K)

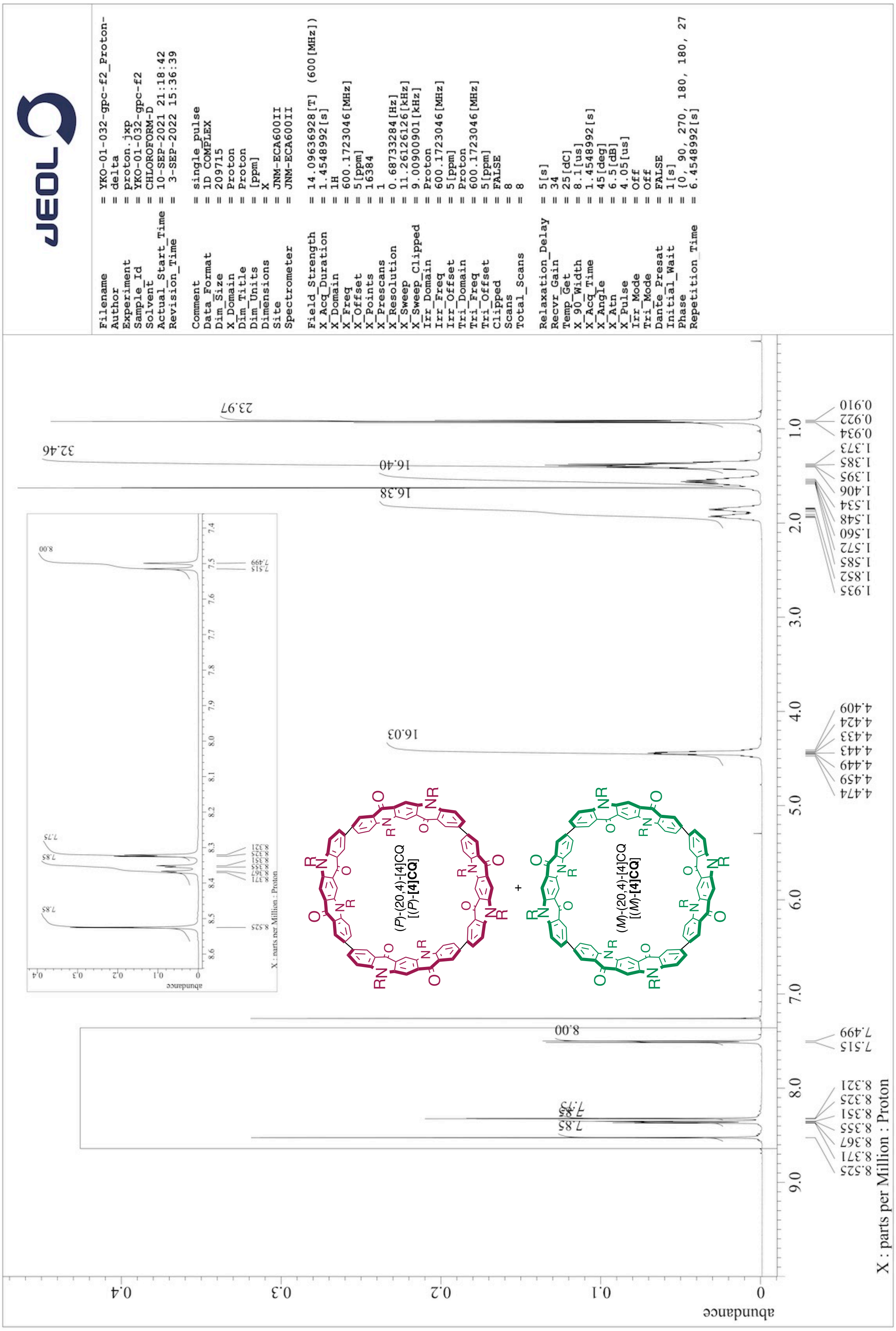

NMR spectrum 3. ^{1}H NMR spectrum of **[4]CQ** ($CDCl_3$, 600 MHz, 298 K)

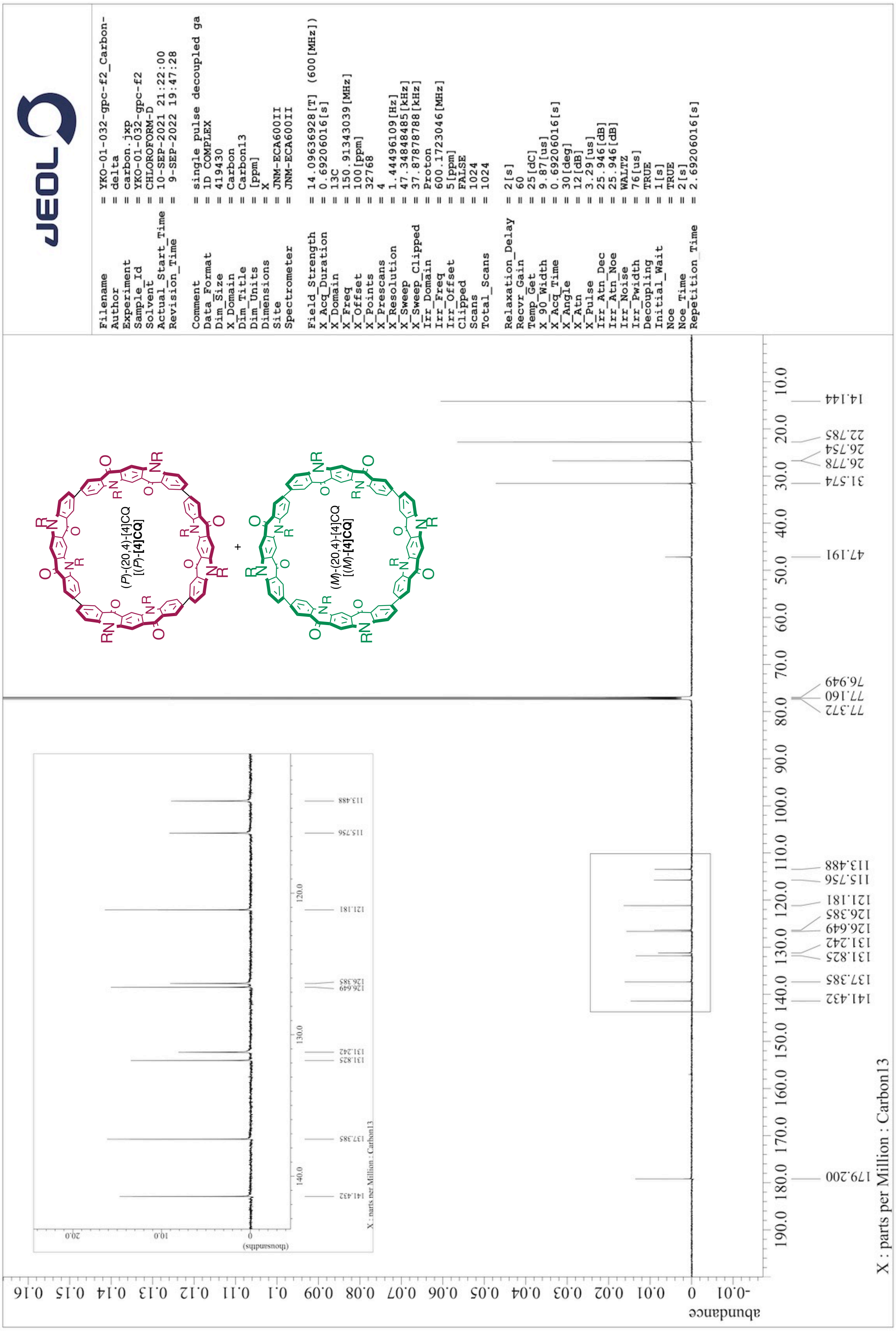

NMR spectrum 4. ^{13}C NMR spectrum of **[4]CQ** ($CDCl_3$, 151 MHz, 298 K)
