# Supplementary material for: Target-oriented design of helical nanotube molecules for rolled incommensurate bilayers
Source: Commun Chem. 2022 Nov 19;5:152. doi: 10.1038/s42004-022-00777-2 (PMC9814558; doi:10.1038/s42004-022-00777-2)
Supplement: Supplementary file 5 — Supplementary Data 3 [file 42004_2022_777_MOESM5_ESM.pdf]

UV-vis and CD spectra of ***[4]CQ***

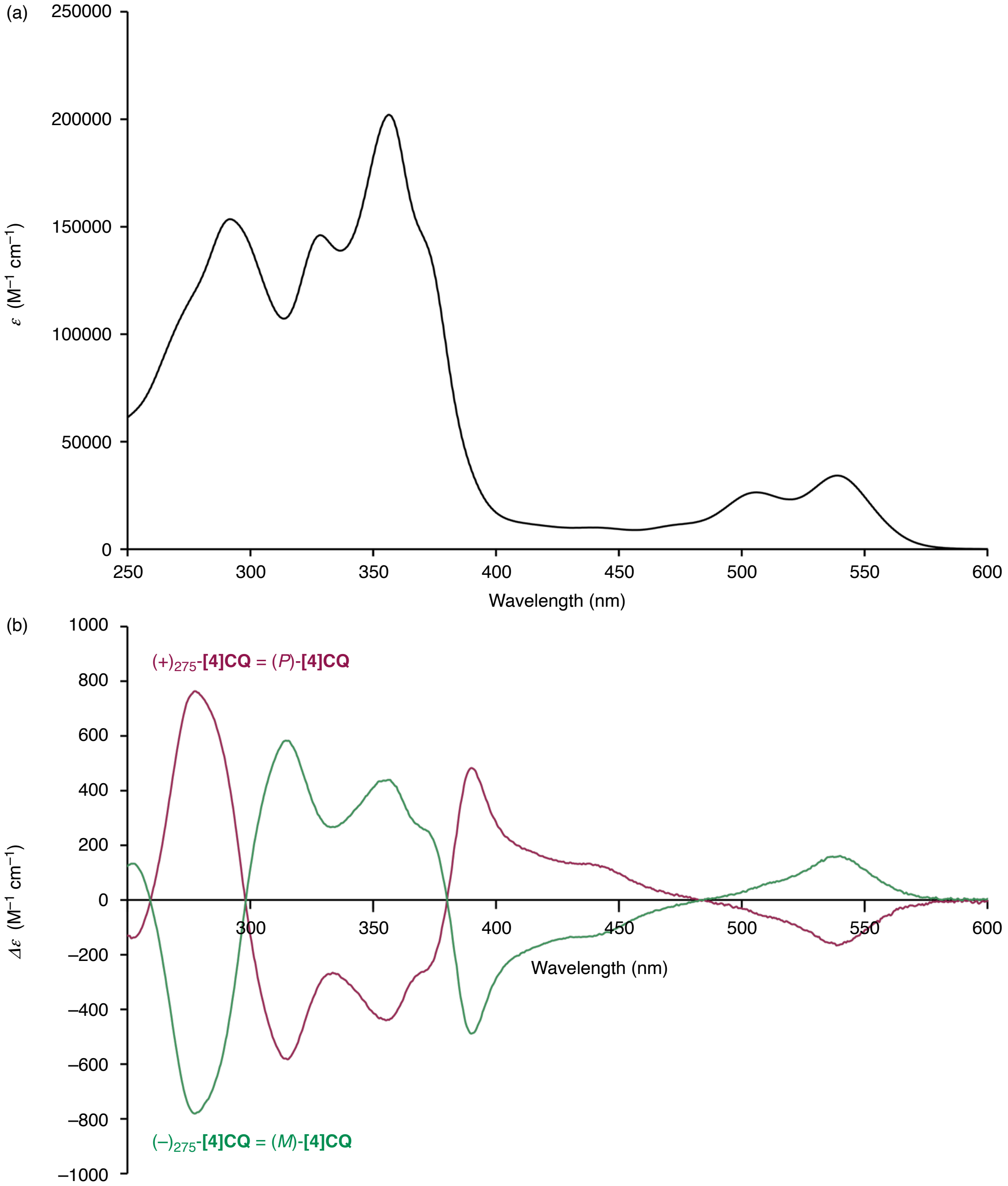

UV-vis and CD Spectra. (a) UV-vis absorption spectrum of *rac*-**[4]CQ** (CH_2Cl_2, 298 K, 4.67 × 10^{-6} M). (b) CD spectra of $(+)_{275}$-**[4]CQ** (CH_2Cl_2, 298 K, 3.02 × 10^{-6} M) and $(–)_{275}$-**[4]CQ** (CH_2Cl_2, 298 K, 3.97 × 10^{-6} M).
