# Supplementary material for: Target-oriented design of helical nanotube molecules for rolled incommensurate bilayers
Source: Commun Chem. 2022 Nov 19;5:152. doi: 10.1038/s42004-022-00777-2 (PMC9814558; doi:10.1038/s42004-022-00777-2)
Supplement: Supplementary file 6 — Supplementary Data 4 [file 42004_2022_777_MOESM6_ESM.pdf]

Titration data (UV-vis)

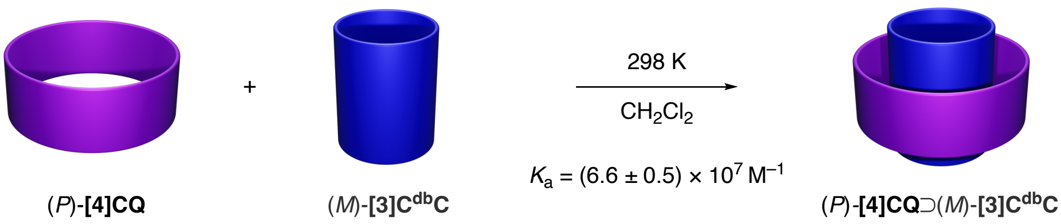

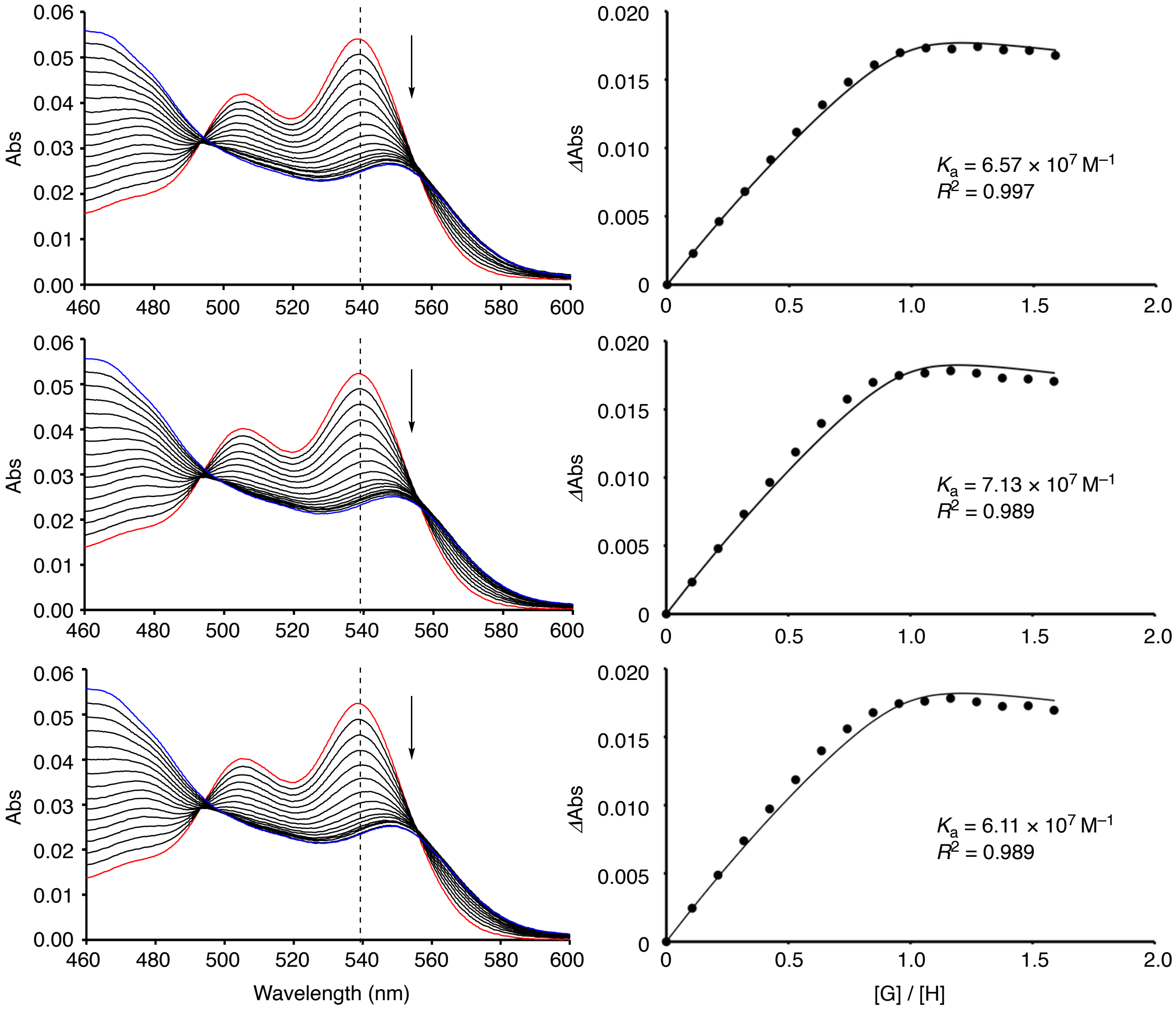

Titration data 1. UV-vis titration experiments for heterohelical complex (*P*)-**[4]CQ**⊃(*M*)-**[3]C^{db}C**

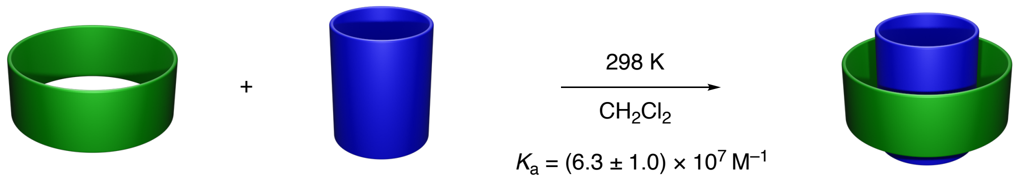

(*M*)-**[4]CQ** (*M*)-**[3]C^{db}C** (*M*)-**[4]CQ**⊃(*M*)-**[3]C^{db}C**

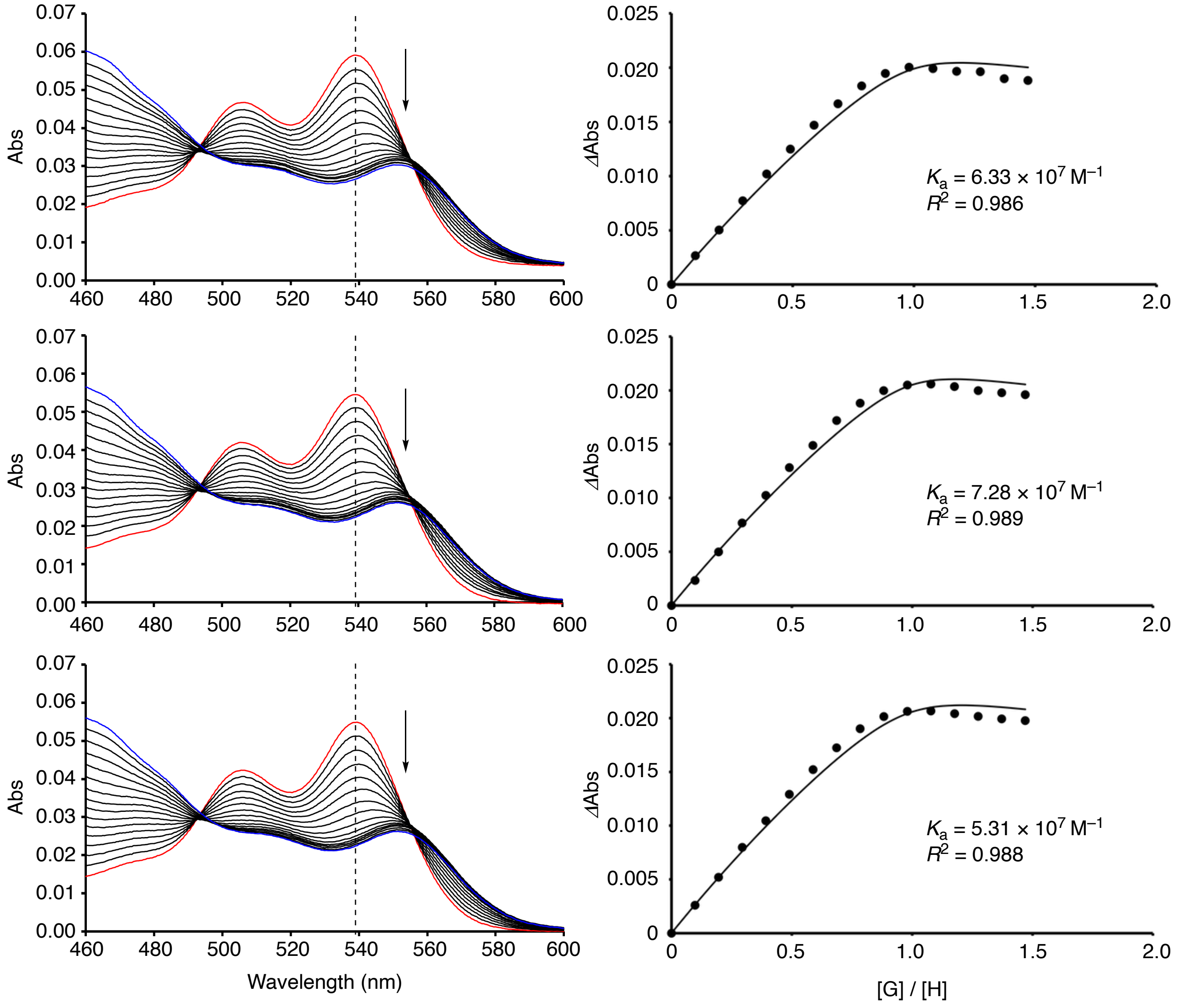

Titration data 2. UV-vis titration experiments for homohelical complex (*M*)-**[4]CQ**⊃(*M*)-**[3]C^{db}C**

Titration data (ITC)

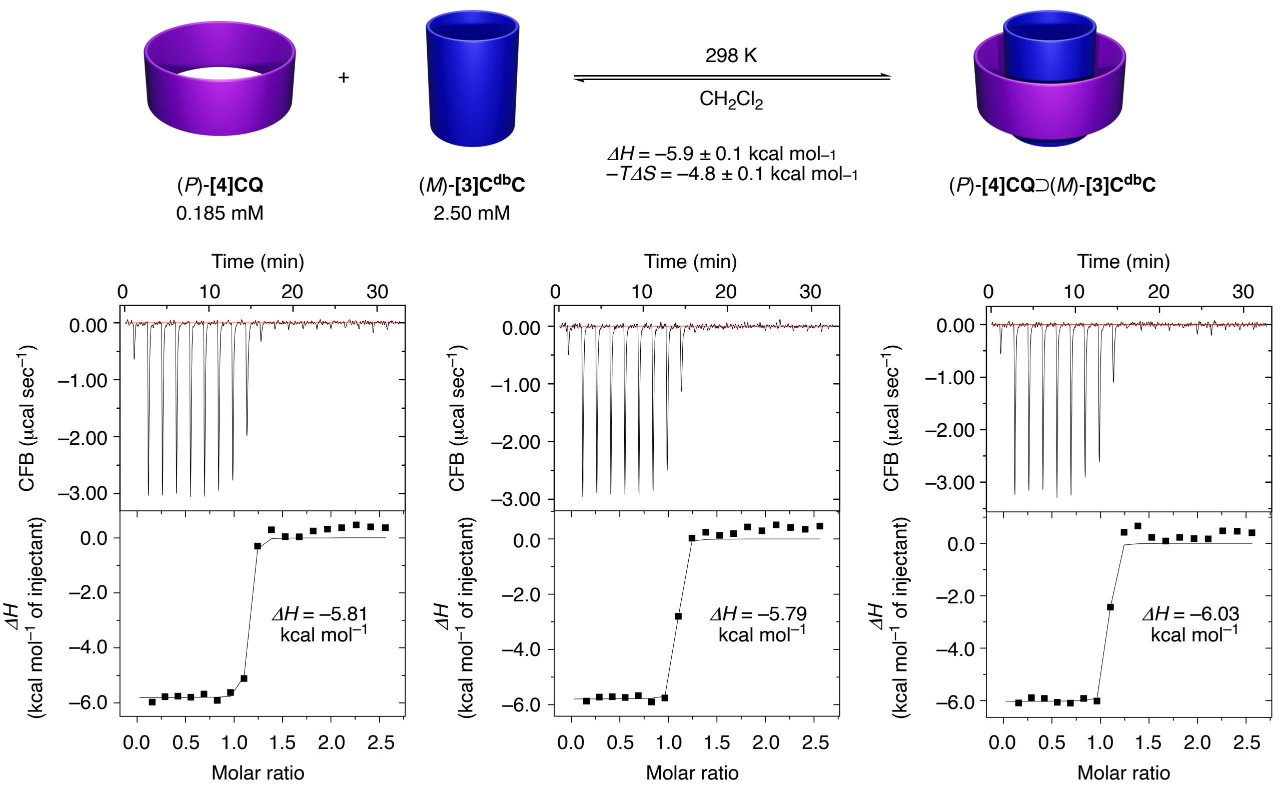

Titration data 3. ITC analyses for heterohelical complex (*P*)-**[4]CQ**⊃(*M*)-**[3]C$^{\mathbf{db}}$C**. CFB = cell feedback.

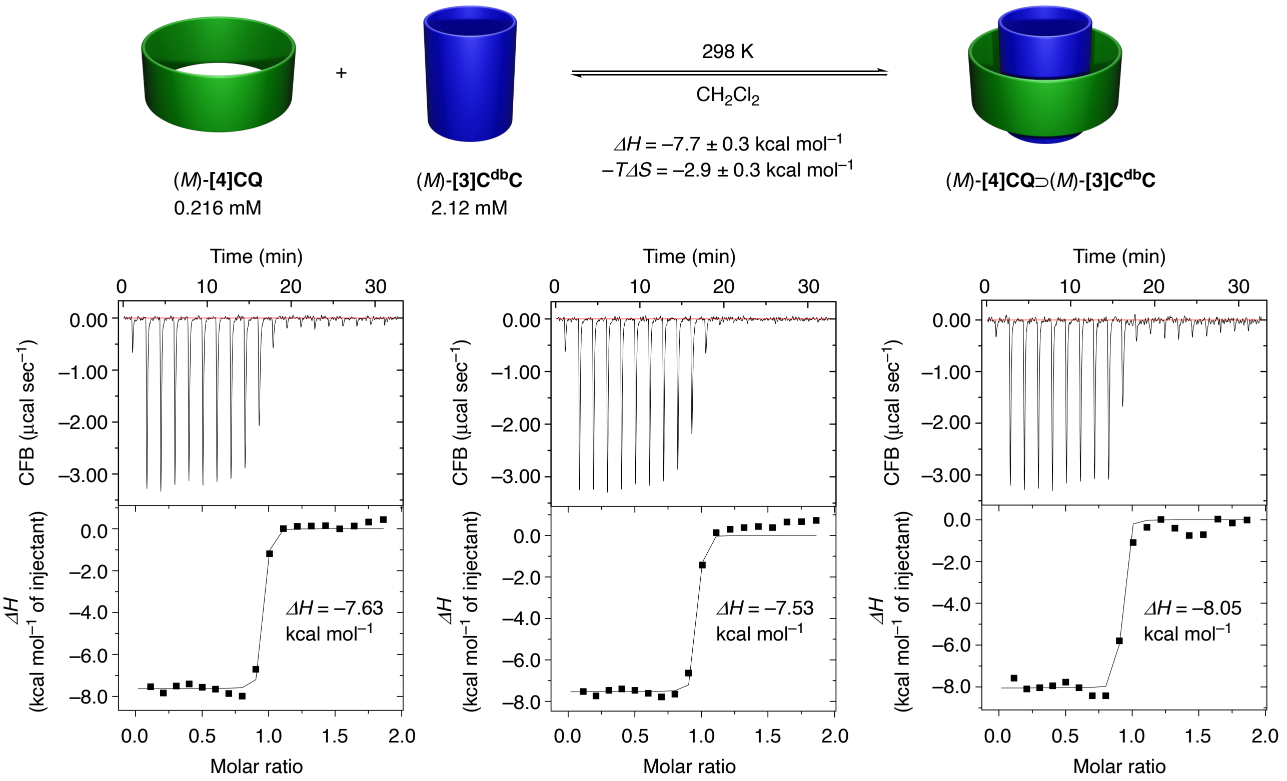

Titration data 4. ITC analyses for homohelical complex (*M*)-**[4]CQ**⊃(*M*)-**[3]C$^{\mathbf{db}}$C**.
