# Supplementary material for: Target-oriented design of helical nanotube molecules for rolled incommensurate bilayers
Source: Commun Chem. 2022 Nov 19;5:152. doi: 10.1038/s42004-022-00777-2 (PMC9814558; doi:10.1038/s42004-022-00777-2)
Supplement: Supplementary file 7 — Supplementary Data 5 [file 42004_2022_777_MOESM7_ESM.pdf]

Theoretical calculations of i-DWNT complexes

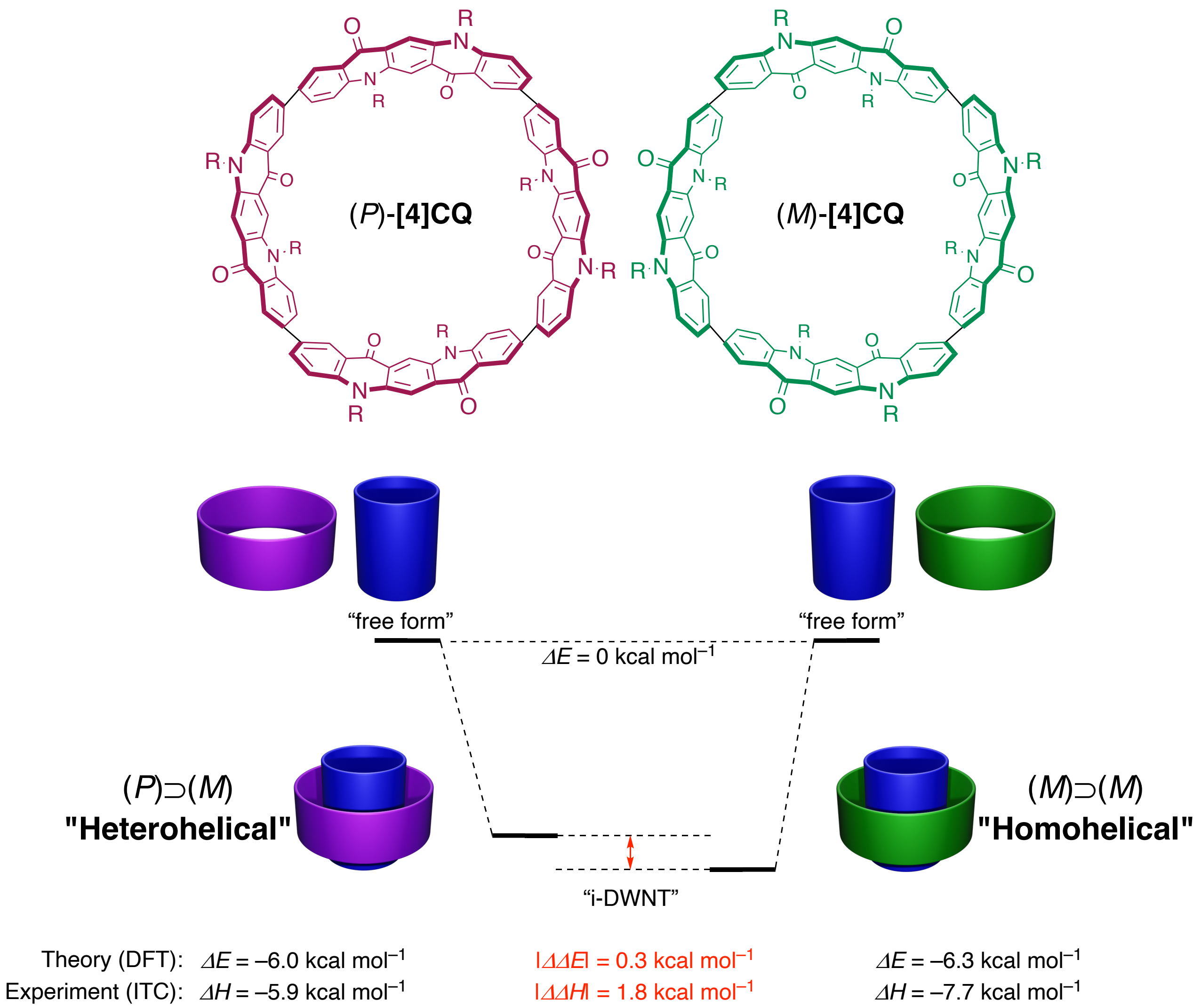

DFT data 1. Theoretical calculations of i-DWNT complexation with **[4]CQ**. DFT calculations were performed at LC-BLYP/6-311G(d) in the presence of PCM (CH_2Cl_2) solvation with BSSE corrections. As references, experimental enthalpy values from ITC analyses are shown. Calculations were performed by adopting methyl-substituted congeners as models.

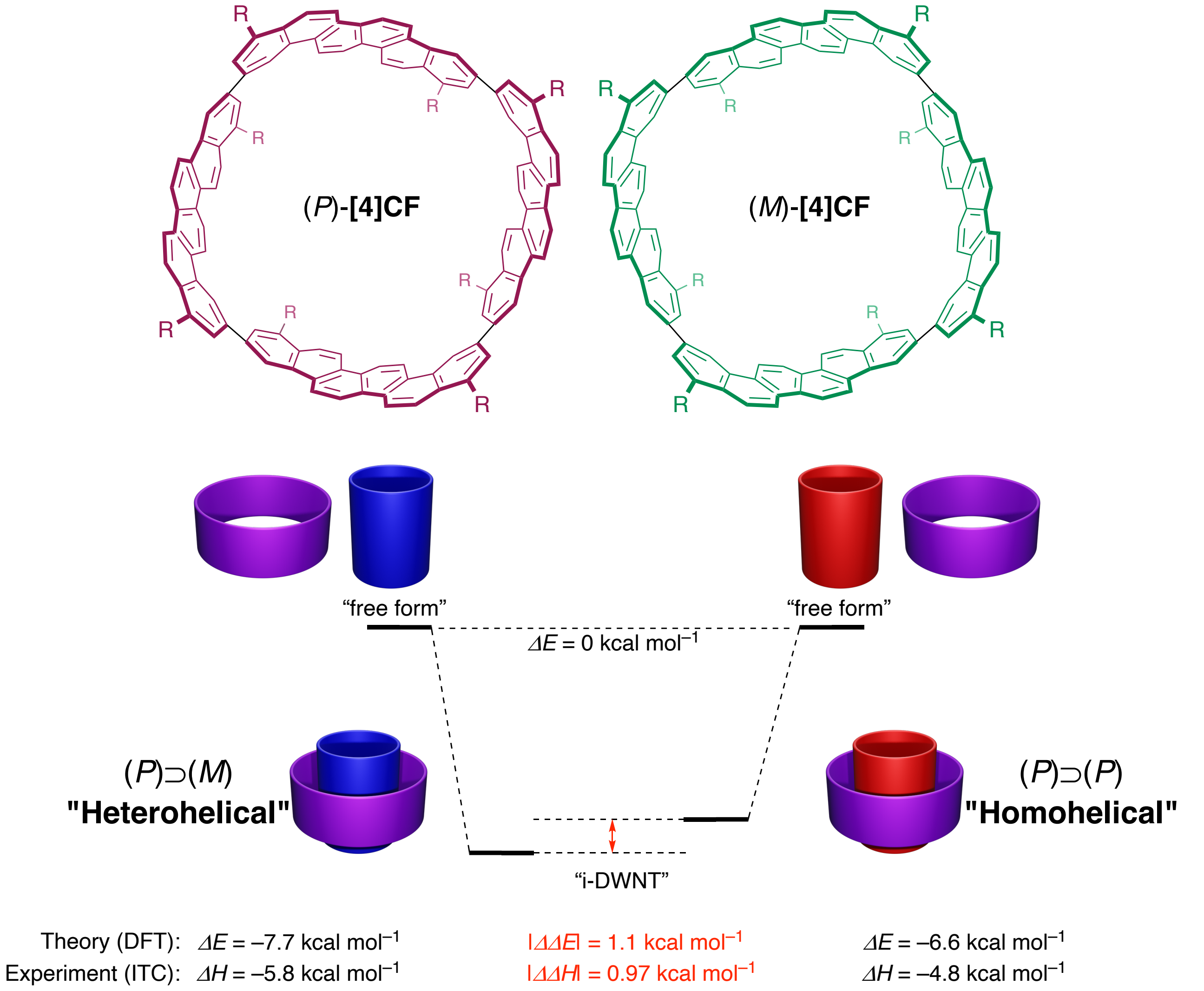

DFT data 2. Theoretical calculations of i-DWNT complexation with **[4]CF**. DFT calculations were performed at LC-BLYP/6-311G(d) in the presence of PCM (CH_2Cl_2) solvation with BSSE corrections. As references, experimental enthalpy values from ITC analyses are shown. Calculations were performed by adopting methyl-substituted congeners as models. Data taken from ref. 8 of the main text.

*Cartesian coordinates of (P)-**[4]CQ** from DFT calculations*

Supplementary Table 3. Cartesian coordinates of methyl-substituted (*P*)-**[4]CQ**

SCF Done: E(RB3LYP) = -4427.18515565 A.U. after 8 cycles

Center	Atomic	Atomic	Coordinates (Angstroms)		
Number	Number	Type	X	Y	Z
1	6	0	8.237624	2.790273	-0.542245
2	6	0	7.773540	3.802809	-1.382811

3	6	0	7.191111	4.965019	-0.867348
4	6	0	7.065296	5.070994	0.542127
5	6	0	7.618808	4.104422	1.382674
6	6	0	8.224857	2.954396	0.867202
7	6	0	6.209814	6.117404	1.143174
8	6	0	5.384888	6.856967	0.173177
9	6	0	5.664759	6.799851	-1.214283
10	6	0	4.858590	7.574623	-2.077697
11	1	0	5.055761	7.603602	-3.141753
12	6	0	3.747169	8.242837	-1.592013
13	6	0	3.371781	8.190788	-0.230483
14	6	0	4.252181	7.541542	0.633627
15	1	0	7.807269	3.590048	-2.444318
16	1	0	7.465662	4.255707	2.444215
17	1	0	3.129468	8.789273	-2.297813
18	1	0	4.053988	7.466965	1.697110
19	6	0	8.827960	0.645253	1.214433
20	6	0	8.711054	0.384267	-0.172963
21	6	0	8.590364	1.485428	-1.143130
22	6	0	8.988401	-0.461236	2.077932
23	6	0	8.607548	-0.935227	-0.633210
24	6	0	8.622595	-2.028932	0.231012
25	6	0	8.883879	-1.753944	1.592445
26	1	0	8.968408	-2.574237	2.298332
27	1	0	8.431254	-1.053011	-1.696634
28	1	0	9.127244	-0.317729	3.141919
29	7	0	8.734671	1.947550	1.694260
30	7	0	6.668843	5.965464	-1.694382
31	8	0	8.676747	1.320185	-2.361351
32	8	0	6.125813	6.283636	2.361432
33	6	0	8.961595	2.225076	3.108502
34	1	0	9.206205	3.279584	3.231095
35	1	0	8.088745	1.987642	3.729753
36	1	0	9.815750	1.645058	3.461211
37	6	0	7.026031	5.988240	-3.108764
38	1	0	6.324811	5.416250	-3.729509
39	1	0	7.051001	7.020313	-3.461719
40	1	0	8.025980	5.573723	-3.231732
41	6	0	-7.065409	-5.071154	0.542097
42	6	0	-7.618843	-4.104538	1.382652
43	6	0	-8.224789	-2.954446	0.867190
44	6	0	-8.237482	-2.790299	-0.542262
45	6	0	-7.773474	-3.802868	-1.382831
46	6	0	-7.191162	-4.965130	-0.867372

47	6	0	-8.590124	-1.485431	-1.143158
48	6	0	-8.710871	-0.384280	-0.172990
49	6	0	-8.827808	-0.645270	1.214395
50	6	0	-8.988276	0.461189	2.077919
51	1	0	-9.127124	0.317637	3.141902
52	6	0	-8.883786	1.753910	1.592459
53	6	0	-8.622486	2.028918	0.231031
54	6	0	-8.607386	0.935229	-0.633210
55	1	0	-7.465713	-4.255875	2.444187
56	1	0	-7.807161	-3.590100	-2.444339
57	1	0	-8.968369	2.574189	2.298356
58	1	0	-8.431079	1.053038	-1.696629
59	6	0	-5.664849	-6.799976	-1.214324
60	6	0	-5.385032	-6.857146	0.173149
61	6	0	-6.210039	-6.117659	1.143137
62	6	0	-4.858611	-7.574709	-2.077716
63	6	0	-4.252309	-7.541671	0.633634
64	6	0	-3.371829	-8.190832	-0.230455
65	6	0	-3.747173	-8.242869	-1.591998
66	1	0	-3.129425	-8.789261	-2.297790
67	1	0	-4.054198	-7.467136	1.697136
68	1	0	-5.055723	-7.603693	-3.141780
69	7	0	-6.668970	-5.965617	-1.694410
70	7	0	-8.734512	-1.947550	1.694243
71	8	0	-6.126170	-6.284006	2.361389
72	8	0	-8.676378	-1.320167	-2.361385
73	6	0	-7.026060	-5.988215	-3.108822
74	1	0	-8.026041	-5.573773	-3.231787
75	1	0	-6.324857	-5.416065	-3.729428
76	1	0	-7.050912	-7.020240	-3.461941
77	6	0	-8.961634	-2.224994	3.108467
78	1	0	-8.088872	-1.987516	3.729836
79	1	0	-9.815835	-1.644948	3.461007
80	1	0	-9.206273	-3.279489	3.231109
81	6	0	-5.071200	7.065269	0.542001
82	6	0	-4.104601	7.618698	1.382576
83	6	0	-2.954485	8.224620	0.867138
84	6	0	-2.790305	8.237310	-0.542310
85	6	0	-3.802858	7.773310	-1.382902
86	6	0	-4.965134	7.191008	-0.867466
87	6	0	-1.485423	8.589951	-1.143176
88	6	0	-0.384291	8.710689	-0.172984
89	6	0	-0.645312	8.827619	1.214398
90	6	0	0.461126	8.988073	2.077950

91	1	0	0.317551	9.126912	3.141931
92	6	0	1.753858	8.883581	1.592516
93	6	0	2.028897	8.622290	0.231094
94	6	0	0.935228	8.607200	-0.633174
95	1	0	-4.255972	7.465593	2.444109
96	1	0	-3.590072	7.807003	-2.444406
97	1	0	2.574123	8.968145	2.298432
98	1	0	1.053067	8.430890	-1.696590
99	6	0	-6.800010	5.664734	-1.214456
100	6	0	-6.857241	5.384950	0.173023
101	6	0	-6.117747	6.209938	1.143021
102	6	0	-7.574719	4.858485	-2.077860
103	6	0	-7.541826	4.252263	0.633508
104	6	0	-8.190988	3.371787	-0.230587
105	6	0	-8.242945	3.747087	-1.592141
106	1	0	-8.789315	3.129328	-2.297941
107	1	0	-7.467336	4.054162	1.697013
108	1	0	-7.603631	5.055554	-3.141932
109	7	0	-5.965604	6.668829	-1.694528
110	7	0	-1.947604	8.734329	1.694215
111	8	0	-6.284129	6.126087	2.361269
112	8	0	-1.320133	8.676221	-2.361398
113	6	0	-5.988132	7.025917	-3.108942
114	1	0	-5.573602	8.025864	-3.231891
115	1	0	-5.416019	6.324669	-3.729530
116	1	0	-7.020143	7.050858	-3.462094
117	6	0	-2.225084	8.961465	3.108429
118	1	0	-1.987611	8.088712	3.729813
119	1	0	-1.645054	9.815677	3.460973
120	1	0	-3.279585	9.206091	3.231042
121	6	0	5.071190	-7.065203	0.541976
122	6	0	4.104592	-7.618621	1.382570
123	6	0	2.954465	-8.224535	0.867152
124	6	0	2.790258	-8.237200	-0.542295
125	6	0	3.802801	-7.773207	-1.382901
126	6	0	4.965100	-7.190926	-0.867487
127	6	0	1.485374	-8.589854	-1.143150
128	6	0	0.384256	-8.710632	-0.172949
129	6	0	0.645290	-8.827533	1.214431
130	6	0	-0.461143	-8.987956	2.077996
131	1	0	-0.317563	-9.126737	3.141983
132	6	0	-1.753880	-8.883493	1.592568
133	6	0	-2.028934	-8.622269	0.231135
134	6	0	-0.935268	-8.607176	-0.633135

135	1	0	4.255990	-7.465491	2.444095
136	1	0	3.589989	-7.806901	-2.444400
137	1	0	-2.574134	-8.968037	2.298499
138	1	0	-1.053105	-8.430883	-1.696554
139	6	0	6.800000	-5.664679	-1.214485
140	6	0	6.857246	-5.384903	0.172993
141	6	0	6.117749	-6.209885	1.142992
142	6	0	7.574741	-4.858461	-2.077891
143	6	0	7.541871	-4.252239	0.633477
144	6	0	8.191052	-3.371781	-0.230618
145	6	0	8.243001	-3.747083	-1.592175
146	1	0	8.789402	-3.129349	-2.297971
147	1	0	7.467399	-4.054137	1.696984
148	1	0	7.603654	-5.055540	-3.141962
149	7	0	5.965562	-6.668743	-1.694562
150	7	0	1.947591	-8.734250	1.694236
151	8	0	6.284146	-6.126047	2.361240
152	8	0	1.320077	-8.676109	-2.361372
153	6	0	5.988062	-7.025784	-3.108988
154	1	0	5.573529	-8.025724	-3.231971
155	1	0	5.415943	-6.324507	-3.729539
156	1	0	7.020068	-7.050718	-3.462159
157	6	0	2.225066	-8.961379	3.108452
158	1	0	1.987570	-8.088633	3.729840
159	1	0	1.645057	-9.815606	3.460995
160	1	0	3.279568	-9.205986	3.231084

Cartesian coordinates of i-DWNT from DFT calculations

Supplementary Table 4. Cartesian coordinates of (*P*)-**[4]CQ**⊃(*M*)-**[3]C^{db}C**.

SCF Done: E(RLC-B+HF-LYP) = -3224.22385847 A.U. after 6 cycles

Center Number	Atomic Number	Atomic Type	Coordinates (Angstroms) X	Y	Z
1	6	0	8.556827	-1.986312	-0.568066
2	6	0	8.622102	-0.886468	-1.393222
3	6	0	8.615045	0.397924	-0.878486
4	6	0	8.524376	0.539349	0.506607
5	6	0	8.564382	-0.564299	1.334441
6	6	0	8.591795	-1.846001	0.818969
7	6	0	8.239138	1.857821	1.100777
8	6	0	7.909685	2.912303	0.137474
9	6	0	8.137708	2.722410	-1.222959
10	6	0	7.824480	3.780508	-2.080879
11	1	0	8.004500	3.700101	-3.143606
12	6	0	7.222227	4.913268	-1.603937
13	6	0	6.889341	5.076167	-0.259163
14	6	0	7.290156	4.070350	0.589817
15	1	0	8.575781	-1.085002	-2.455219
16	1	0	8.481954	-0.372716	2.396597
17	1	0	6.972038	5.693027	-2.313745
18	1	0	7.080994	4.107303	1.651985
19	6	0	7.987000	-4.133884	1.146723
20	6	0	7.774654	-4.300153	-0.218494
21	6	0	8.278088	-3.305033	-1.169358
22	6	0	7.535557	-5.146316	1.995826
23	6	0	6.988477	-5.344697	-0.683173
24	6	0	6.384867	-6.241295	0.167803
25	6	0	6.746405	-6.154067	1.513670
26	1	0	6.349483	-6.872432	2.221386
27	1	0	6.798726	-5.351780	-1.748758
28	1	0	7.714046	-5.091029	3.060565

29	7	0	8.564224	-2.976769	1.628576
30	7	0	8.615800	1.520060	-1.698421
31	8	0	8.359110	-3.506465	-2.358462
32	8	0	8.200690	2.029189	2.298490
33	6	0	8.970281	-2.878543	3.007290
34	1	0	9.710989	-2.089288	3.108132
35	1	0	8.139391	-2.665312	3.685057
36	1	0	9.441782	-3.808478	3.319521
37	6	0	9.004330	1.392216	-3.080759
38	1	0	8.151883	1.231414	-3.747750
39	1	0	9.533877	2.286805	-3.401211
40	1	0	9.691213	0.557973	-3.189220
41	6	0	-8.759886	-0.686650	0.546584
42	6	0	-8.659077	0.411634	1.369932
43	6	0	-8.460722	1.679888	0.852989
44	6	0	-8.349528	1.803989	-0.532164
45	6	0	-8.555262	0.717481	-1.358318
46	6	0	-8.774822	-0.544580	-0.840770
47	6	0	-7.868863	3.063585	-1.128101
48	6	0	-7.386512	4.058611	-0.166078
49	6	0	-7.642742	3.907609	1.194174
50	6	0	-7.176891	4.908669	2.051260
51	1	0	-7.369498	4.858227	3.113625
52	6	0	-6.410634	5.937238	1.574034
53	6	0	-6.053877	6.045348	0.229801
54	6	0	-6.599195	5.109819	-0.618873
55	1	0	-8.641762	0.210207	2.432278
56	1	0	-8.445807	0.892831	-2.420882
57	1	0	-6.047847	6.672356	2.282863
58	1	0	-6.384398	5.113451	-1.680636
59	6	0	-8.519383	-2.897410	-1.165359
60	6	0	-8.332630	-3.091729	0.199841
61	6	0	-8.681741	-2.031534	1.149560
62	6	0	-8.224241	-3.966502	-2.013780
63	6	0	-7.709598	-4.240846	0.665172

64	6	0	-7.247166	-5.218097	-0.185296
65	6	0	-7.593205	-5.079741	-1.531037
66	1	0	-7.308681	-5.850302	-2.237927
67	1	0	-7.521584	-4.274978	1.730576
68	1	0	-8.394055	-3.886363	-3.078372
69	7	0	-8.918489	-1.668008	-1.648265
70	7	0	-8.295066	2.790923	1.671020
71	8	0	-8.792149	-2.217163	2.338837
72	8	0	-7.803649	3.224928	-2.326103
73	6	0	-9.309168	-1.512852	-3.026143
74	1	0	-9.921624	-0.620469	-3.127387
75	1	0	-8.457716	-1.430547	-3.706800
76	1	0	-9.917598	-2.361226	-3.334107
77	6	0	-8.701154	2.725984	3.052706
78	1	0	-7.882726	2.445144	3.722470
79	1	0	-9.095453	3.689358	3.368831
80	1	0	-9.502408	2.001067	3.162030
81	6	0	-0.594179	8.855051	0.536828
82	6	0	0.515627	8.848752	1.356090
83	6	0	1.790690	8.751087	0.831343
84	6	0	1.915309	8.675294	-0.556796
85	6	0	0.816490	8.833430	-1.375675
86	6	0	-0.459657	8.919092	-0.850988
87	6	0	3.182866	8.221458	-1.155248
88	6	0	4.143790	7.670537	-0.199727
89	6	0	4.004390	7.926501	1.161590
90	6	0	4.979459	7.404467	2.017290
91	1	0	4.940204	7.603405	3.078873
92	6	0	5.942515	6.559036	1.540372
93	6	0	6.015719	6.184852	0.195876
94	6	0	5.133287	6.806786	-0.654710
95	1	0	0.333147	8.792899	2.419971
96	1	0	0.989479	8.756749	-2.439887
97	1	0	6.651466	6.143839	2.247566
98	1	0	5.123107	6.593701	-1.716147

99	6	0	-2.771628	8.432368	-1.183513
100	6	0	-2.946941	8.192535	0.176561
101	6	0	-1.913885	8.586668	1.134072
102	6	0	-3.814671	8.066932	-2.040450
103	6	0	-4.055054	7.485823	0.628755
104	6	0	-5.022378	7.009527	-0.223376
105	6	0	-4.894232	7.374230	-1.566306
106	1	0	-5.658388	7.074131	-2.274303
107	1	0	-4.075785	7.267247	1.688871
108	1	0	-3.746622	8.263210	-3.101101
109	7	0	-1.590890	8.958641	-1.655839
110	7	0	2.915113	8.620972	1.636009
111	8	0	-2.089069	8.572121	2.334385
112	8	0	3.356933	8.191636	-2.355410
113	6	0	-1.469987	9.346022	-3.040412
114	1	0	-0.629781	10.026492	-3.149502
115	1	0	-1.321873	8.488903	-3.700656
116	1	0	-2.363343	9.885956	-3.348859
117	6	0	2.850538	9.012734	3.023141
118	1	0	2.576271	8.182666	3.677502
119	1	0	3.813452	9.412570	3.335656
120	1	0	2.120103	9.809363	3.136123
121	6	0	0.641394	-8.429901	0.505802
122	6	0	-0.448482	-8.390561	1.349076
123	6	0	-1.739500	-8.313046	0.858187
124	6	0	-1.892577	-8.235155	-0.526303
125	6	0	-0.809102	-8.358946	-1.369668
126	6	0	0.478532	-8.479619	-0.878767
127	6	0	-3.189297	-7.850029	-1.108232
128	6	0	-4.208584	-7.449680	-0.139435
129	6	0	-4.018681	-7.672762	1.221737
130	6	0	-5.044121	-7.271856	2.085155
131	1	0	-4.971150	-7.450602	3.148363
132	6	0	-6.123628	-6.576275	1.613389
133	6	0	-6.265232	-6.233565	0.267150

134	6	0	-5.315504	-6.739117	-0.588023
135	1	0	-0.228011	-8.336547	2.406628
136	1	0	-1.018452	-8.269135	-2.427065
137	1	0	-6.873181	-6.252910	2.327032
138	1	0	-5.343440	-6.529587	-1.649890
139	6	0	2.827747	-8.187898	-1.241444
140	6	0	3.048827	-7.998478	0.120134
141	6	0	1.981505	-8.246717	1.088326
142	6	0	3.901412	-7.942653	-2.104394
143	6	0	4.248845	-7.460493	0.569718
144	6	0	5.263054	-7.099759	-0.284987
145	6	0	5.072269	-7.416008	-1.631652
146	1	0	5.861482	-7.206458	-2.345004
147	1	0	4.307357	-7.259303	1.632005
148	1	0	3.802719	-8.106936	-3.167880
149	7	0	1.586546	-8.560005	-1.711106
150	7	0	-2.847028	-8.226999	1.690567
151	8	0	2.157579	-8.208103	2.287367
152	8	0	-3.356041	-7.778936	-2.307205
153	6	0	1.401223	-8.848386	-3.111126
154	1	0	0.512386	-9.458577	-3.241838
155	1	0	1.298698	-7.941844	-3.715074
156	1	0	2.241765	-9.428036	-3.485238
157	6	0	-2.707380	-8.542639	3.090030
158	1	0	-2.469769	-7.662903	3.695558
159	1	0	-3.625582	-8.989957	3.463172
160	1	0	-1.920559	-9.279905	3.219547
161	6	0	-3.026297	4.013622	-1.060595
162	6	0	-1.898356	4.805976	-0.880487
163	6	0	-1.400299	5.025939	0.402861
164	6	0	-2.199734	4.598495	1.470304
165	6	0	-3.312381	3.840496	1.269111
166	6	0	-3.689480	3.430964	-0.011034
167	1	0	-3.273778	3.733837	-2.073628
168	1	0	-3.838067	3.476352	2.140999

169	6	0	0.624972	5.465405	1.881899
170	6	0	1.929313	5.018960	2.057114
171	6	0	2.518984	5.125005	3.317797
172	6	0	1.846472	5.680499	4.366844
173	6	0	0.561404	6.193411	4.192382
174	6	0	-0.021490	6.080162	2.959407
175	1	0	3.541348	4.794908	3.455512
176	1	0	2.328064	5.759533	5.336657
177	6	0	0.009603	5.345754	0.568490
178	6	0	0.823318	5.281302	-0.528829
179	6	0	2.889689	4.200130	-1.429829
180	6	0	3.871177	3.279168	-1.228533
181	6	0	4.179699	2.815683	0.051629
182	6	0	3.615294	3.494166	1.101302
183	6	0	2.624417	4.452841	0.920922
184	6	0	2.166383	4.746812	-0.362453
185	1	0	4.334455	2.838719	-2.100491
186	1	0	3.816448	3.179022	2.114325
187	6	0	-0.940238	5.896907	-4.326965
188	6	0	0.409213	6.202261	-4.153424
189	6	0	0.967905	5.998045	-2.920942
190	6	0	0.234199	5.492951	-1.842773
191	6	0	-1.123929	5.255914	-2.017309
192	6	0	-1.690521	5.452606	-3.277529
193	1	0	-1.404365	6.050783	-5.296316
194	1	0	-2.752031	5.286805	-3.414460
195	1	0	-0.987741	6.541945	2.808743
196	1	0	2.599293	4.403160	-2.447717
197	1	0	1.994822	6.301827	-2.771920
198	1	0	-1.881341	4.755000	2.488130
199	6	0	1.206293	-5.006783	0.983287
200	6	0	2.528777	-4.602535	0.851374
201	6	0	3.024663	-4.238345	-0.398003
202	6	0	2.201887	-4.475114	-1.503573
203	6	0	0.905351	-4.864554	-1.350698

204	6	0	0.342745	-5.041763	-0.085545
205	1	0	0.834924	-5.158551	1.986445
206	1	0	0.288448	-4.912079	-2.237421
207	6	0	4.704062	-2.884316	-1.737772
208	6	0	5.186226	-1.587432	-1.842934
209	6	0	5.722917	-1.164765	-3.059621
210	6	0	5.829369	-2.017531	-4.116434
211	6	0	5.411378	-3.343483	-4.005220
212	6	0	4.843582	-3.747628	-2.829505
213	1	0	6.102018	-0.153696	-3.149849
214	1	0	6.278288	-1.677684	-5.044139
215	6	0	4.154851	-3.329781	-0.470857
216	6	0	4.559401	-2.703692	0.674147
217	6	0	4.936948	-0.446077	1.678199
218	6	0	4.861852	0.904214	1.519091
219	6	0	4.790674	1.481658	0.250053
220	6	0	5.013710	0.648483	-0.817191
221	6	0	5.113715	-0.730284	-0.678923
222	6	0	4.980077	-1.314735	0.581152
223	1	0	4.758495	1.510825	2.407993
224	1	0	4.960837	1.038793	-1.822512
225	6	0	3.991446	-4.843075	4.280936
226	6	0	4.983421	-3.866824	4.226230
227	6	0	5.145898	-3.167546	3.060026
228	6	0	4.340227	-3.387774	1.940199
229	6	0	3.358533	-4.371151	2.015260
230	6	0	3.201327	-5.094774	3.196299
231	1	0	3.861152	-5.424598	5.187896
232	1	0	2.480391	-5.903153	3.235406
233	1	0	4.541662	-4.782392	-2.719470
234	1	0	4.833424	-0.835131	2.678507
235	1	0	5.960509	-2.458991	2.986207
236	1	0	2.535673	-4.212388	-2.495566
237	6	0	-1.969918	-4.755842	-0.965368
238	6	0	-3.213621	-4.151413	-0.832755

239	6	0	-3.648131	-3.718512	0.417442
240	6	0	-2.872969	-4.083342	1.522683
241	6	0	-1.652367	-4.668747	1.369139
242	6	0	-1.123143	-4.927610	0.103583
243	1	0	-1.625852	-4.960561	-1.968811
244	1	0	-1.050760	-4.813740	2.255838
245	6	0	-5.099086	-2.124503	1.760134
246	6	0	-5.373814	-0.768706	1.869621
247	6	0	-5.842888	-0.272471	3.086311
248	6	0	-6.084265	-1.102477	4.139216
249	6	0	-5.876201	-2.476679	4.023804
250	6	0	-5.373932	-2.959645	2.847930
251	1	0	-6.060466	0.784956	3.179662
252	1	0	-6.478195	-0.700512	5.067071
253	6	0	-4.623282	-2.645447	0.492293
254	6	0	-4.924346	-1.960271	-0.650816
255	6	0	-4.945698	0.332239	-1.646595
256	6	0	-4.661677	1.653732	-1.482703
257	6	0	-4.500748	2.208692	-0.211789
258	6	0	-4.850741	1.416801	0.852858
259	6	0	-5.165408	0.071036	0.709349
260	6	0	-5.123714	-0.522956	-0.552709
261	1	0	-4.466198	2.239819	-2.369831
262	1	0	-4.737200	1.790521	1.859608
263	6	0	-4.685162	-4.146966	-4.266469
264	6	0	-5.512529	-3.027962	-4.210187
265	6	0	-5.569872	-2.317850	-3.040477
266	6	0	-4.812260	-2.665721	-1.919346
267	6	0	-3.995295	-3.789693	-1.996595
268	6	0	-3.948754	-4.524136	-3.180438
269	1	0	-4.642811	-4.736946	-5.176331
270	1	0	-3.361971	-5.434615	-3.221787
271	1	0	-5.235052	-4.028300	2.734682
272	1	0	-4.904354	-0.064219	-2.648425
273	1	0	-6.264615	-1.491382	-2.965751

274	1	0	-3.163140	-3.775060	2.515328
275	6	0	1.222310	6.760002	-5.279333
276	1	0	1.515970	5.973656	-5.978206
277	1	0	2.131265	7.235374	-4.910750
278	1	0	0.661314	7.501794	-5.850463
279	6	0	-0.153889	6.870381	5.319269
280	1	0	-0.996004	7.458629	4.955063
281	1	0	0.509412	7.537957	5.872146
282	1	0	-0.540184	6.140037	6.033624
283	6	0	5.642779	-4.300157	-5.130102
284	1	0	5.332612	-3.877466	-6.086905
285	1	0	5.097190	-5.232533	-4.984313
286	1	0	6.704457	-4.543385	-5.207993
287	6	0	5.880239	-3.638401	5.402876
288	1	0	6.369317	-2.665492	5.356863
289	1	0	6.661607	-4.400772	5.460668
290	1	0	5.323541	-3.681848	6.339460
291	6	0	-6.354837	-2.655167	-5.390308
292	1	0	-7.254088	-3.274140	-5.448428
293	1	0	-5.810901	-2.795785	-6.324756
294	1	0	-6.671821	-1.613212	-5.348944
295	6	0	-6.257045	-3.390261	5.143816
296	1	0	-5.896085	-3.020044	6.104515
297	1	0	-5.854904	-4.393145	4.999844
298	1	0	-7.343737	-3.473222	5.211702

Supplementary Table 5. Cartesian coordinates of (*M*)-**[4]CQ**⊃(*M*)-**[3]C^{db}C**.

SCF Done: E(RLC-B+HF-LYP) = -3224.22414566 A.U. after 6 cycles

Center Number	Atomic Number	Atomic Type	Coordinates (Angstroms) X	Y	Z
1	6	0	0.818910	8.707174	0.195257
2	6	0	1.892201	8.472913	-0.635706

3	6	0	3.174517	8.264896	-0.145684
4	6	0	3.413728	8.270405	1.226126
5	6	0	2.368480	8.675549	2.059266
6	6	0	1.113889	8.876003	1.549289
7	6	0	4.264852	7.945677	-1.073303
8	6	0	5.427208	7.036116	0.921334
9	6	0	5.351111	7.153319	-0.465062
10	6	0	6.166593	6.404066	-1.285010
11	1	0	6.052451	6.565065	-2.348237
12	6	0	6.988406	5.417553	-0.769681
13	6	0	6.972410	5.219838	0.612365
14	6	0	6.247432	6.052252	1.440292
15	1	0	1.770704	8.358985	-1.705914
16	1	0	2.519645	8.759226	3.126972
17	1	0	0.319017	9.134190	2.239384
18	1	0	6.267944	5.825819	2.498753
19	6	0	8.106374	3.331178	-1.135197
20	6	0	8.017066	3.011008	0.216335
21	6	0	7.591220	4.015739	1.193091
22	1	0	8.656664	2.525450	-3.062156
23	6	0	8.543491	2.328052	-2.005672
24	6	0	8.248508	1.711936	0.647166
25	6	0	8.579221	0.695155	-0.216384
26	6	0	8.767200	1.057042	-1.550979
27	1	0	8.086959	1.528933	1.702393
28	1	0	9.074512	0.305012	-2.268737
29	7	0	4.622208	7.835496	1.723574
30	7	0	7.739279	4.580803	-1.587363
31	8	0	7.652979	3.834143	2.388654
32	8	0	4.232664	8.205006	-2.253647
33	6	0	4.960247	8.024820	3.111946
34	1	0	4.492320	7.284109	3.762776
35	1	0	6.039371	7.958230	3.227661
36	1	0	4.660400	9.022065	3.429435
37	6	0	7.956898	4.944258	-2.964643

38	1	0	7.187320	4.545214	-3.633077
39	1	0	7.972730	6.026554	-3.056660
40	1	0	8.927964	4.582419	-3.294420
41	6	0	8.576920	-0.722743	0.215636
42	6	0	8.243050	-1.738464	-0.647933
43	6	0	8.007334	-3.036773	-0.217094
44	6	0	8.095409	-3.357193	1.134452
45	6	0	8.535702	-2.355474	2.004957
46	6	0	8.763584	-1.085213	1.550265
47	6	0	7.578381	-4.040148	-1.193879
48	6	0	6.970752	-5.439947	0.768821
49	6	0	6.955573	-5.242201	-0.613211
50	6	0	6.227991	-6.072275	-1.441248
51	1	0	6.249372	-5.845909	-2.499706
52	6	0	5.404544	-7.053458	-0.922407
53	6	0	5.327919	-7.170433	0.463996
54	6	0	6.145679	-6.423828	1.284047
55	1	0	8.082271	-1.554978	-1.703196
56	1	0	8.648127	-2.553219	3.061456
57	1	0	9.073265	-0.334170	2.268036
58	1	0	6.030870	-6.584485	2.347252
59	6	0	3.387122	-8.281272	-1.227443
60	6	0	3.147794	-8.275021	0.144349
61	6	0	4.239070	-7.959348	1.072096
62	1	0	2.491603	-8.767007	-3.128391
63	6	0	2.340643	-8.682961	-2.060682
64	6	0	1.864804	-8.478954	0.634265
65	6	0	0.790818	-8.709733	-0.196797
66	6	0	1.085350	-8.879388	-1.550807
67	1	0	1.743612	-8.364712	1.704471
68	1	0	0.289698	-9.134913	-2.240997
69	7	0	7.724190	-4.605604	1.586602
70	7	0	4.597098	-7.850305	-1.724734
71	8	0	4.205917	-8.218617	2.252418
72	8	0	7.640940	-3.858795	-2.389439

73	6	0	7.940369	-4.969668	2.963946
74	1	0	7.172035	-4.567957	3.632213
75	1	0	7.952545	-6.051998	3.056067
76	1	0	8.912599	-4.611078	3.293848
77	6	0	4.934886	-8.040989	-3.112968
78	1	0	4.469650	-7.298977	-3.764259
79	1	0	6.014263	-7.977966	-3.228373
80	1	0	4.631880	-9.037324	-3.430326
81	6	0	-0.608574	-8.616700	0.288690
82	6	0	-1.646659	-8.257705	-0.540784
83	6	0	-2.908903	-7.930301	-0.060201
84	6	0	-3.160780	-7.930487	1.309551
85	6	0	-2.148718	-8.406531	2.148186
86	6	0	-0.920414	-8.736083	1.643858
87	6	0	-3.941070	-7.493817	-1.004377
88	6	0	-5.186161	-6.690711	0.992625
89	6	0	-5.088749	-6.794766	-0.394952
90	6	0	-5.963657	-6.115800	-1.217290
91	1	0	-5.817460	-6.244145	-2.281942
92	6	0	-6.894263	-5.228759	-0.704490
93	6	0	-6.881933	-5.011620	0.674625
94	6	0	-6.077942	-5.765286	1.501757
95	1	0	-1.507988	-8.144975	-1.608835
96	1	0	-2.300829	-8.468175	3.216531
97	1	0	-0.157626	-9.064339	2.339964
98	1	0	-6.119389	-5.523586	2.555312
99	6	0	-8.270600	-3.302788	-1.068411
100	6	0	-8.189700	-2.950464	0.275718
101	6	0	-7.607828	-3.867841	1.253050
102	1	0	-8.939160	-2.601306	-2.996617
103	6	0	-8.833217	-2.370849	-1.946373
104	6	0	-8.537292	-1.668296	0.685009
105	6	0	-8.947745	-0.700030	-0.197203
106	6	0	-9.142585	-1.109407	-1.518586
107	1	0	-8.379278	-1.438994	1.730146

108	1	0	-9.508291	-0.395939	-2.248376
109	7	0	-4.344233	-7.433562	1.810933
110	7	0	-7.755118	-4.501440	-1.515348
111	8	0	-7.624299	-3.654159	2.447364
112	8	0	-3.827174	-7.611828	-2.204045
113	6	0	-4.635807	-7.538888	3.218551
114	1	0	-4.124686	-6.775789	3.813375
115	1	0	-5.706163	-7.441673	3.374887
116	1	0	-4.347604	-8.521478	3.585431
117	6	0	-8.006881	-4.937195	-2.866739
118	1	0	-7.361111	-4.441778	-3.596018
119	1	0	-7.856395	-6.010909	-2.934292
120	1	0	-9.044333	-4.739600	-3.129609
121	6	0	-0.580756	8.618576	-0.290263
122	6	0	-1.619977	8.262977	0.539255
123	6	0	-2.883252	7.939542	0.058715
124	6	0	-3.135134	7.940362	-1.311036
125	6	0	-2.121565	8.413103	-2.149730
126	6	0	-0.892213	8.738811	-1.645438
127	6	0	-3.916811	7.506495	1.002943
128	6	0	-5.164455	6.707066	-0.993953
129	6	0	-5.066696	6.811008	0.393607
130	6	0	-5.943740	6.134932	1.216054
131	1	0	-5.797144	6.262931	2.280690
132	6	0	-6.877155	5.250788	0.703382
133	6	0	-6.865602	5.033466	-0.675712
134	6	0	-6.059222	5.784447	-1.502952
135	1	0	-1.481667	8.149901	1.607314
136	1	0	-2.273494	8.475131	-3.218077
137	1	0	-0.128377	9.064559	-2.341576
138	1	0	-6.101496	5.542779	-2.556486
139	6	0	-8.259624	3.329284	1.067645
140	6	0	-8.180161	2.976667	-0.276494
141	6	0	-7.595287	3.892009	-1.253958
142	1	0	-8.929593	2.629902	2.996111

143	6	0	-8.824885	2.399147	1.945807
144	6	0	-8.532071	1.695633	-0.685667
145	6	0	-8.945371	0.728708	0.196684
146	6	0	-9.138427	1.138699	1.518141
147	1	0	-8.375110	1.465782	-1.730841
148	1	0	-9.506104	0.426403	2.248080
149	7	0	-4.320144	7.447085	-1.812366
150	7	0	-7.740297	4.526355	1.514389
151	8	0	-7.612504	3.678240	-2.448248
152	8	0	-3.802556	7.624332	2.202592
153	6	0	-4.611373	7.553310	-3.219987
154	1	0	-4.102059	6.789059	-3.814877
155	1	0	-5.681942	7.458705	-3.376411
156	1	0	-4.320761	8.535254	-3.586726
157	6	0	-7.990504	4.963247	2.865697
158	1	0	-7.345327	4.466990	3.594930
159	1	0	-7.837930	6.036723	2.932618
160	1	0	-9.028183	4.767769	3.129183
161	6	0	-1.050210	4.962274	-0.865042
162	6	0	0.310325	5.175525	-0.685258
163	6	0	0.872989	5.063967	0.585293
164	6	0	-0.014291	4.922040	1.657450
165	6	0	-1.346314	4.722090	1.458516
166	6	0	-1.883506	4.631290	0.173917
167	1	0	-1.412693	4.921303	-1.881557
168	1	0	-1.956901	4.524626	2.328054
169	6	0	2.924127	4.533766	1.998929
170	6	0	3.933344	3.584309	2.103287
171	6	0	4.567213	3.394030	3.331046
172	6	0	4.207484	4.121505	4.427024
173	6	0	3.215335	5.096781	4.336801
174	6	0	2.606431	5.297224	3.126902
175	1	0	5.395288	2.700504	3.401782
176	1	0	4.721492	3.965070	5.369850
177	6	0	2.287972	4.747251	0.708437

178	6	0	2.977485	4.406678	-0.421965
179	6	0	4.375317	2.601012	-1.444444
180	6	0	4.883078	1.343247	-1.313136
181	6	0	4.989773	0.730317	-0.063801
182	6	0	4.780478	1.531960	1.030937
183	6	0	4.292330	2.828526	0.922240
184	6	0	3.974113	3.350099	-0.331696
185	1	0	5.098959	0.789853	-2.216886
186	1	0	4.846700	1.115974	2.025477
187	6	0	1.628627	6.094870	-4.060109
188	6	0	2.970193	5.749556	-3.934018
189	6	0	3.381980	5.163886	-2.767228
190	6	0	2.517659	4.942411	-1.694983
191	6	0	1.191246	5.343192	-1.820863
192	6	0	0.760753	5.896273	-3.024323
193	1	0	1.279525	6.556860	-4.977847
194	1	0	-0.267465	6.222786	-3.119445
195	1	0	1.888917	6.101209	3.029742
196	1	0	4.166881	2.952264	-2.442462
197	1	0	4.431430	4.935745	-2.646924
198	1	0	0.356041	4.829480	2.665597
199	6	0	-1.065851	-4.959231	0.866404
200	6	0	0.294046	-5.176569	0.686547
201	6	0	0.857025	-5.066348	-0.583976
202	6	0	-0.029801	-4.921353	-1.656094
203	6	0	-1.361182	-4.717366	-1.457095
204	6	0	-1.898106	-4.625352	-0.172459
205	1	0	-1.428248	-4.917481	1.882922
206	1	0	-1.971120	-4.517677	-2.326576
207	6	0	2.909752	-4.542351	-1.997567
208	6	0	3.922008	-3.596114	-2.101802
209	6	0	4.556487	-3.407736	-3.329519
210	6	0	4.194422	-4.133940	-4.425587
211	6	0	3.199121	-5.105988	-4.335494
212	6	0	2.589589	-5.304638	-3.125608

213	1	0	5.386794	-2.716883	-3.400217
214	1	0	4.708948	-3.979038	-5.368384
215	6	0	2.272952	-4.753985	-0.707090
216	6	0	2.963528	-4.415766	0.423357
217	6	0	4.366833	-2.614500	1.446064
218	6	0	4.878561	-1.358314	1.314879
219	6	0	4.987393	-0.745689	0.065592
220	6	0	4.775648	-1.546613	-1.029211
221	6	0	4.283371	-2.841611	-0.920645
222	6	0	3.963442	-3.362274	0.333240
223	1	0	5.095991	-0.805617	2.218687
224	1	0	4.843286	-1.130792	-2.023720
225	6	0	1.609737	-6.100968	4.061030
226	6	0	2.952332	-5.759565	3.935009
227	6	0	3.365823	-5.174770	2.768405
228	6	0	2.502111	-4.950401	1.696244
229	6	0	1.174512	-5.347253	1.822052
230	6	0	0.742421	-5.899460	3.025361
231	1	0	1.259319	-6.562237	4.978630
232	1	0	-0.286769	-6.222929	3.120412
233	1	0	1.869474	-6.106305	-3.028574
234	1	0	4.157123	-2.965134	2.444039
235	1	0	4.415947	-4.949717	2.648121
236	1	0	0.340825	-4.829602	-2.664207
237	6	0	-3.876880	-3.363776	-0.984272
238	6	0	-4.747670	-2.294584	-0.821030
239	6	0	-4.948723	-1.751262	0.446683
240	6	0	-4.372118	-2.422071	1.529966
241	6	0	-3.508954	-3.457532	1.342143
242	6	0	-3.165805	-3.896312	0.062127
243	1	0	-3.655899	-3.672687	-1.995034
244	1	0	-3.011264	-3.859560	2.213947
245	6	0	-5.603188	0.273816	1.846282
246	6	0	-5.299484	1.624057	1.969989
247	6	0	-5.520829	2.256361	3.192443

248	6	0	-6.061504	1.580795	4.247560
249	6	0	-6.413014	0.236543	4.126616
250	6	0	-6.173398	-0.394033	2.935233
251	1	0	-5.283833	3.308975	3.296101
252	1	0	-6.236211	2.095332	5.187717
253	6	0	-5.398292	-0.376731	0.563982
254	6	0	-5.397006	0.393554	-0.562581
255	6	0	-4.364394	2.435741	-1.528437
256	6	0	-3.498069	3.468544	-1.340567
257	6	0	-3.153445	3.906129	-0.060546
258	6	0	-3.866060	3.375667	0.985865
259	6	0	-4.740269	2.309272	0.822552
260	6	0	-4.943070	1.766655	-0.445198
261	1	0	-2.999273	3.869177	-2.212377
262	1	0	-3.643984	3.683715	1.996657
263	6	0	-6.065869	-1.561859	-4.246292
264	6	0	-6.412936	-0.216442	-4.125468
265	6	0	-6.171547	0.413346	-2.934034
266	6	0	-5.603823	-0.256345	-1.844908
267	6	0	-5.304563	-1.607591	-1.968528
268	6	0	-5.527731	-2.239193	-3.191018
269	1	0	-6.242024	-2.075831	-5.186489
270	1	0	-5.294251	-3.292601	-3.294592
271	1	0	-6.470567	-1.431024	2.830244
272	1	0	-4.492394	2.058497	-2.531032
273	1	0	-6.465303	1.451311	-2.829174
274	1	0	-4.498970	-2.044344	2.532532
275	6	0	3.954910	6.098650	-5.002290
276	1	0	3.493616	6.116479	-5.989931
277	1	0	4.784827	5.391548	-5.034340
278	1	0	4.368557	7.090420	-4.806131
279	6	0	2.845391	5.908305	5.539190
280	1	0	2.174574	6.727955	5.281235
281	1	0	3.725771	6.332944	6.025530
282	1	0	2.336836	5.292191	6.283169

283	6	0	2.826492	-5.916089	-5.538006
284	1	0	2.320115	-5.298154	-6.281964
285	1	0	2.152847	-6.733493	-5.280248
286	1	0	3.705448	-6.343680	-6.024320
287	6	0	3.935955	-6.111892	5.003227
288	1	0	4.768707	-5.408107	5.034548
289	1	0	4.345594	-7.105444	4.807689
290	1	0	3.474877	-6.127181	5.991018
291	6	0	-7.037505	0.513470	-5.273022
292	1	0	-7.924098	-0.006522	-5.640824
293	1	0	-6.342974	0.594716	-6.111638
294	1	0	-7.335264	1.522056	-4.987827
295	6	0	-7.040216	-0.491336	5.274027
296	1	0	-6.345755	-0.575786	6.112388
297	1	0	-7.342114	-1.498592	4.988484
298	1	0	-7.924575	0.032077	5.642341
